# Can metformin prolong pregnancy in preterm pre-eclampsia? Study protocol for a Nordic multicentre randomised placebo-controlled trial (PI4)

**DOI:** 10.1136/bmjopen-2026-120993

**Published:** 2026-10-13

**Authors:** Pia Gudmundsson, Catherine Anne Cluver, Anna Sandström, Linda Lindström, Simon Timpka, Susanne Hesselman, Caroline Lilliecreutz, Sophia Brismar Wendel, Emma Von Wowern, Karolina Linden, Ola Andersson, Hanna Gyllensten, Mårten Alkmark, Emma Norrman, Erica Wesslén, Angla Mantel, Marie Blomberg, Hugo Aronzon, Hannele Laivuori, Pia Villa, Trond Melbye Michelsen, Ingvil Krarup Sørbye, Camilla Haavaldsen, Marie-Therese Vinnars, Eleonor Tiblad, Nadine Raab, Elli Toivonen, Ann Hellström, Karin Sävman, Hanna Ohlsson, Stina Berver, Emelie Ottosson, Carina Bejlum, Merit Kullinger, Karin Stenfeldt, Susan P Walker, Stephen Tong, Henrik Imberg, Lina Bergman

**Affiliations:** 1 Department of Obstetrics and Gynaecology, University of Gothenburg, Gothenburg, Sweden; 2 Department of Obstetrics and Gynaecology, Sahlgrenska University Hospital, Gothenburg, Sweden; 3 SAMRC Preeclampsia Extramural Research Unit, Stellenbosch University and Tygerberg Hospital, Cape Town, South Africa; 4 Department of Obstetrics, Gynaecology and Newborn Health, Mercy Hospital for Women, The University of Melbourne Melbourne Medical School, Melbourne, Victoria, Australia; 5 Mercy Perinatal, Mercy Hospital for Women, Heidelberg, Victoria, Australia; 6 Clinical Epidemiology Division, Department of Medicine, Karolinska Institutet, Stockholm, Sweden; 7 Theme Women’s Health, Department of Obstetrics, Karolinska University Hospital, Stockholm, Sweden; 8 Department of Women's and Children’s Health, Uppsala University, Uppsala, Sweden; 9 Department of Obstetrics and Gynaecology, Uppsala University Hospital, Uppsala, Sweden; 10 Perinatal and Cardiovascular Epidemiology, Department of Clinical Sciences Malmö, Lund University, Lund, Sweden; 11 Department of Obstetrics and Gynaecology, Skåne University Hospital Lund, Lund, Sweden; 12 Department of Obstetrics and Gynaecology, Skåne University Hospital, Malmö, Sweden; 13 Department of Obstetrics and Gynaecology, Falu lasarett, Falun, Sweden; 14 Clinical Department of Obstetrics and Gynaecology, Linköping University Hospital, Linköping, Sweden; 15 Department of Biomedical and Clinical Sciences, Linköping University, Linköping, Sweden; 16 Department of Clinical Sciences, Danderyd Hospital, Karolinska Institutet, Stockholm, Sweden; 17 Department of Women’s Health, Danderyd Hospital, Stockholm, Sweden; 18 Institute of Health and Care Sciences, Sahlgrenska Academy, University of Gothenburg, Gothenburg, Sweden; 19 Department of Clinical Sciences, Lund, Paediatrics, Lund University Faculty of Medicine, Lund, Sweden; 20 Department of Neonatology, Skåne University Hospital Lund, Lund, Sweden; 21 Faculty of Medicine and Health Technology, Centre for Child, Adolescent, and Maternal Health Research, Tampere Universities, Tampere, Pirkanmaa, Finland; 22 Department of Obstetrics and Gynaecology, Tampere University Hospital, Tampere, Pirkanmaa, Finland; 23 Medical and Clinical Genetics, University of Helsinki and Helsinki University Hospital, HUS Helsinki University Hospital, Helsinki, Finland; 24 Institute for Molecular Medicine Finland, Helsinki Institute of Life Science, HiLIFE, University of Helsinki, Helsinki, Uusimaa, Finland; 25 Department of Obstetrics and Gynaecology, Helsinki University Hospital and University of Helsinki, HUS Helsinki University Hospital, Helsinki, Uusimaa, Finland; 26 Department of Obstetrics, Oslo University Hospital, Oslo, Norway; 27 Institute of Clinical Medicine, Faculty of Medicine, University of Oslo, Oslo, Norway; 28 Department of Obstetrics and Gynaecology, Akershus University Hospital, Lørenskog, Norway; 29 Department of Clinical Sciences, Obstetrics and Gynaecology, Umeå University, Umeå, Sweden; 30 Department of Obstetrics and Gynaecology, Norrland University Hospital, Umeå, Västerbotten County, Sweden; 31 Department of Clinical Neuroscience, University of Gothenburg Institute of Neuroscience and Physiology, Gothenburg, Sweden; 32 Department of Paediatrics, Queen Silvia Children’s Hospital, Sahlgrenska University Hospital, Gothenburg, Sweden; 33 Department of Paediatrics, University of Gothenburg, Gothenburg, Sweden; 34 Department of Neonatology, Queen Silvia Children’s Hospital, Sahlgrenska University Hospital, Gothenburg, Sweden; 35 Department of Obstetrics and Gynaecology, Södra Älvsborgs Hospital, Borås, Sweden; 36 Department of Obstetrics and Gynaecology, Skaraborg Hospital Skövde, Skövde, Sweden; 37 Department of Obstetrics and Gynaecology, Norra Älvsborg County Hospital, Trollhättan, Sweden; 38 Department of Obstetrics and Gynaecology, Västerås Central Hospital, Västerås, Sweden; 39 Preeclampsia Patient Organization, Lund, Sweden; 40 Statistiska Konsultgruppen Sweden AB, Gothenburg, Sweden; 41 Wallenberg Centre of Molecular and Translational Medicine, University of Gothenburg, Gothenburg, Sweden

**Keywords:** Randomized Controlled Trial, Pregnancy, Maternal medicine, Fetal medicine

## Abstract

**Introduction:**

Pre-eclampsia affects 2–8% of all pregnancies and remains a leading cause of maternal and perinatal morbidity and mortality. A randomised trial in South Africa suggested metformin used to treat preterm pre-eclampsia (26–32 weeks’ gestation) may prolong pregnancy by 7 days compared with standard care. However, this finding was borderline significant and requires validation in higher-resource settings and in diverse populations. Here, we present a Nordic multicentre trial protocol designed to evaluate whether extended-release metformin can prolong gestation in women with preterm pre-eclampsia.

**Methods and analysis:**

Pre-eclampsia Intervention 4 trial is a phase III, double-blind, multicentre randomised controlled trial conducted in Sweden, Norway and Finland aiming to investigate whether metformin can prolong gestation in preterm pre-eclampsia. A total of 294 women between 22+0 and 33+6 weeks of gestation with pre-eclampsia and planned to undergo expectant management will be enrolled. Participants are randomised to receive one gram of extended-release metformin or matching placebo three times daily. The primary outcome is prolongation of gestation defined as time from randomisation to birth. Secondary outcomes include length of neonatal hospital stay and neonatal birth weight. Adverse events will be reported in line with current clinical trial guidelines. All efficacy analyses will be conducted according to the intention-to-treat principle.

**Ethics and dissemination:**

The trial is conducted in accordance with Good Clinical Practice, as defined by the International Council for Harmonisation. The protocol, including amendments, was approved by the Swedish Ethical Review Authority and the Swedish Medical Products Agency in accordance with national legislation, and authorised for Finland and Norway through the Clinical Trials Information System in accordance with the EU Clinical Trials Regulation. Results will be disseminated at international conferences and published in peer-reviewed journals and communicated via popular science outlets, media, patient organisations and the trial website: https://www.preeclampsiaresearch.com/pi-trials.

**Trial registration number:**

NCT06033131.

**STRENGTHS AND LIMITATIONS OF THIS STUDY**

- This is a large multicentre trial conducted in three Nordic countries: Sweden, Norway and Finland.
- With a sample size of 294 participants, the trial is powered to detect a 50% relative increase in prolongation of gestation from randomisation to birth, corresponding to an absolute increase of 3.5 days assuming a median prolongation of 7 days in the placebo group.
- The randomised double-blind, placebo-controlled design minimises the risk of bias, while the multicentre setting enhances the generalisability of the findings.
- The long-term follow-up provides an opportunity to evaluate the child’s cognitive development and maternal health 2 years postpartum.
- This trial is not powered to detect differences in adverse neonatal clinical outcomes or other rare events, but individual participant data meta-analyses combining results from this and three other ongoing or finalised metformin vs placebo trials are planned.

## Introduction

 Pre-eclampsia is a leading cause of maternal mortality, accounting for 60 000 maternal deaths and an even higher number of fetal losses globally each year.^1^ The disease is progressive and currently, the only treatment to stop progression is birth and removal of the placenta. When pre-eclampsia is diagnosed preterm (<37 weeks’ gestation), this often results in iatrogenic preterm birth resulting in severe complications for the neonate. These include cerebral palsy, intracerebral haemorrhage, retinopathy of prematurity, chronic lung disease and death.^2^ A treatment to delay disease progression and prolong pregnancy would be important to mitigate these outcomes. There have only been a few trials regarding investigational agents for preterm pre-eclampsia. They have evaluated treatments such as phosphodiesterase inhibitors, proton pump inhibitors and statins to alter the disease progression of preterm pre-eclampsia. Still, none of those trials have identified an efficacious, safe treatment for pre-eclampsia.^3–7^

Metformin is safe to use during pregnancy for both mother and fetus.^8–15^ A preclinical study strongly suggests that metformin may be a candidate therapeutic for preeclampsia.^16^ First, in vitro and ex vivo experiments using primary human tissues showed that metformin reduces the placental release of anti-angiogenic factors, namely soluble Fms-like tyrosine kinase 1 and soluble Endoglin, that cause maternal endothelial dysfunction in preeclampsia. Second, metformin enhances vasodilation in vessels isolated from maternal omental arteries obtained at caesarean section and induces angiogenesis.^16^ These characteristics make it a potential therapeutic to treat preeclampsia. Further preclinical studies show that metformin can rescue a preeclampsia phenotype in an animal model.^17^ Although metformin is generally considered to mediate its antihyperglycaemic effects through specific cellular pathways, recent evidence indicates that it also acts through pathways in the gut. For example, a study has provided evidence that the lower bowel is a major site of action for metformin.^18^ Furthermore, recent studies in mice^19^ and humans^20 21^ suggest that gut microbial changes contribute to the effects of metformin.^21^ Members of our research team in South Africa have shown that metformin extended-release (ER) was associated with a median 7.6 day prolongation of gestation compared with placebo (geometric mean ratio 1.39, 95% CI 0.99 to 1.95; p=0.057) in women diagnosed with preterm pre-eclampsia (the Pre-eclampsia Intervention 2, PI2 trial).^8 22^ The metformin ER group also showed numerically higher birth weight, shorter neonatal intensive care unit stay and shorter duration of neonatal care, although these differences were not statistically significant.^8^

Currently, the pre-eclampsia intervention (PI) 3 trial is ongoing at the same trial site as the PI2 trial. While PI2 enrolled 180 participants, PI3 aims to recruit 500 participants, providing sufficient power to assess neonatal outcomes.^23^ However, to date, all trials investigating metformin ER for prolonging pregnancy have been conducted in one site in South Africa, a low-and middle-income country where viability is considered only from 27+0 weeks of gestation and labour is initiated at the latest by 34+0 weeks of gestation. In contrast, in the Nordic countries, pregnancies are considered viable from 22+0 or 23+0 weeks of gestation and expectant management of pre-eclampsia is continued up to 37+0 weeks of gestation. Furthermore, the Nordic countries represent a high-income setting with a different demography, and a comprehensive, free-of-charge maternal healthcare system that reaches essentially all pregnant women. Thus, nearly all pregnant women are screened for pre-eclampsia from second trimester by blood pressure measurements and urinary dipsticks, improving early detection of disease. In addition, prophylactic aspirin treatment is provided to women at high risk of pre-eclampsia by first trimester risk factor-based screening.

Here, we present a clinical trial protocol for a Nordic multicentre trial designed to assess whether metformin ER can prolong pregnancy in women with preterm pre-eclampsia. Participants and their children will be followed until 2 years postpartum with a focus on the child’s cognitive development, the mother’s mental and physical health, and the treatment’s cost-effectiveness. Results from this trial will be analysed separately but also be part of meta-analyses combining results from this and three other ongoing or finalised metformin versus placebo trials in South Africa and The Netherlands.

### Objectives

To examine whether one gram of metformin ER three times daily can prolong gestation and improve neonatal outcomes in pregnancies complicated by preterm pre-eclampsia diagnosed between 22+0 and 33+6 weeks of gestation, compared with placebo, in a Nordic multicentre trial.

## Methods and analysis

The protocol with appendices is available on the European Clinical Trials webpage (https://euclinicaltrials.eu/search-for-clinical-trials/?lang=en&EUCT=2022-502707-29-00), and the protocol without appendices is available in online supplemental appendix 1. A Gantt chart illustrating the trial timeline is provided in figure 1. The trial is supported by the Swedish Network for National Clinical Studies in Obstetrics and Gynaecology (SNAKS, https://www.snaks.se/), and the Nordic Alliance for Clinical Trials in Obstetrics and Gynaecology (NORD-ACT, https://nfog.org/nordic-research/). The authors used ChatGPT (GPT-5; OpenAI) during manuscript preparation to assist with language editing and improve readability. All AI-generated suggestions were critically reviewed, verified, and edited by the authors, who take full responsibility for the final content of the manuscript.

**Figure 1:**
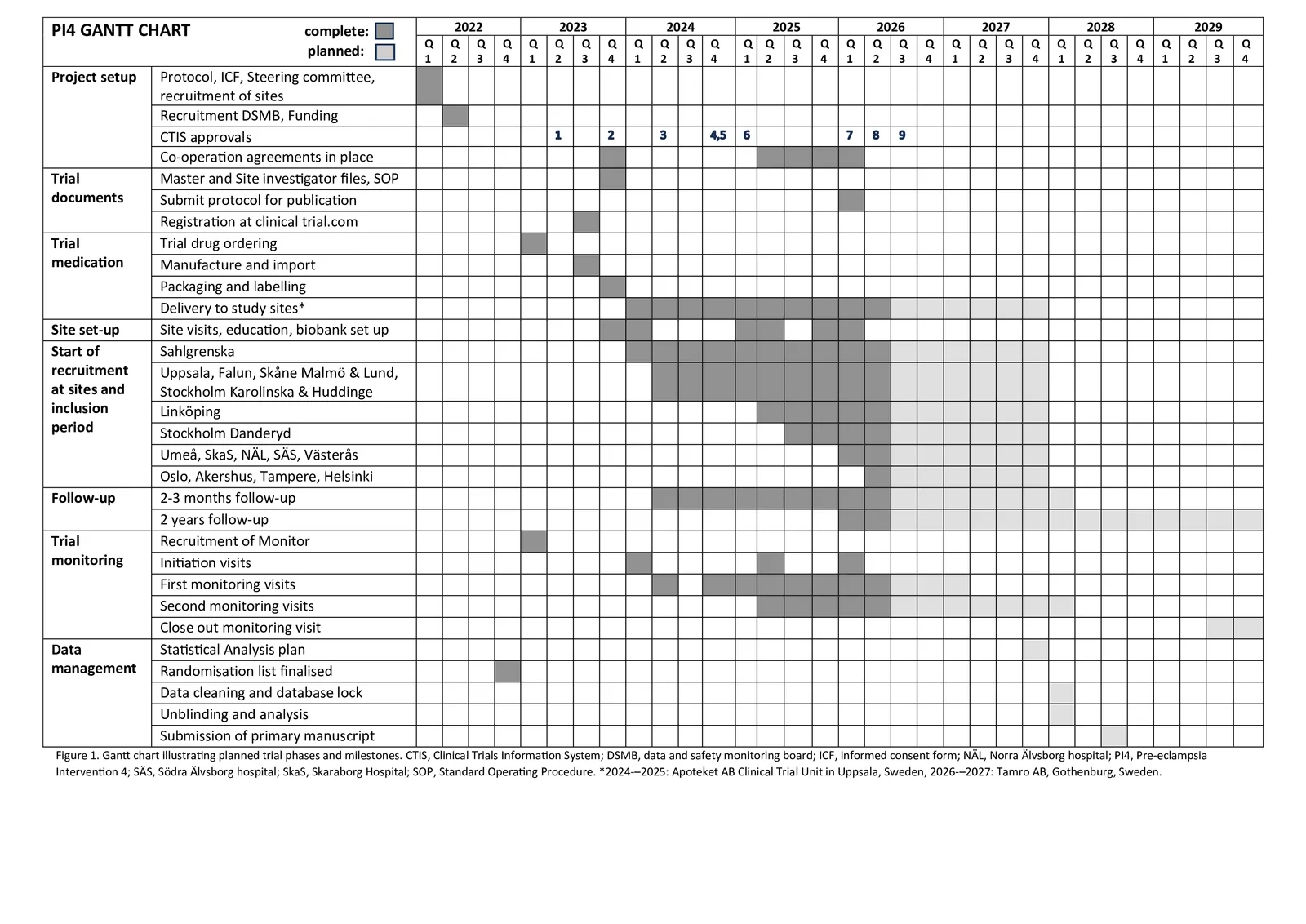
Gantt chart illustrating planned trial phases and milestones. CTIS, Clinical Trials Information System; DSMB, data and safety monitoring board; ICF, informed consent form; NÄL, Norra Älvsborg hospital; PI4, Pre-eclampsia Intervention 4; SÄS: Södra Älvsborg hospital; SkaS: Skaraborg Hospital; SOP, Standard Operating Procedure. *2024–2025: Apoteket AB Clinical Trial Unit in Uppsala, Sweden, 2026–2027: Tamro AB, Gothenburg, Sweden.

### Trial design

This is a phase III, academic-led, double-blind, Nordic multicentre randomised placebo-controlled trial that started in February 2024.

### Trial population

Pregnant women who present with preterm pre-eclampsia between 22+0 and 33+6 weeks of gestation at the included sites are invited to participate. The participating Swedish main sites are Sahlgrenska University Hospital, Falu Lasarett, Skåne University Hospital Lund, Skåne University Hospital Malmö, Karolinska University Hospital Huddinge, Karolinska University Hospital Solna, Uppsala University Hospital, Linköping University Hospital, Danderyd Hospital and Norrland University Hospital. The Norwegian main sites are Oslo University Hospital and Akershus University Hospital, and the Finnish main sites are Tampere University Hospital and Helsinki University Hospital. In addition, four satellite sites associated with Swedish main sites are participating including Södra Älvsborg Hospital, Norra Älvsborg Hospital, Skaraborg Hospital and Västmanland Hospital. See Gantt Chart in figure 1 for the chronological activation of study sites throughout the clinical trial. Additional sites may be included if recruitment targets are not achieved.

### Inclusion criteria

Eligible participants are women with a diagnosis of pre-eclampsia who are considered suitable for expectant management, have provided written informed consent, are aged 18 years or older, are between 22+0 and 33+6 weeks of gestation with a viable fetus and have a singleton pregnancy. If the gestational age is between 22+0 to 23+6 gestational weeks, the sponsor should be contacted for discussion around viability before randomisation. A complete list of inclusion criteria is provided in box 1.

Box 1 **Inclusion and exclusion criteria**

**Inclusion criteria**

- A diagnosis of pre-eclampsia (defined as hypertension in combination with significant proteinuria (albumin/creatinine ratio>8 mg/mmol, protein/creatinine ratio>30 mg/mmol or>2+ protein on a urinary dipstick) has been made by the attending clinician.
- The managing clinicians have made the assessment to proceed with expectant management.
- The woman has given written consent to participate in the study.
- The woman must be 18 years of age or older.
- The gestational age is between 22+0 weeks to 33+6 weeks with a viable fetus.
- The woman carries a singleton pregnancy.

**Exclusion criteria**

- Contraindications to treatment with metformin as outlined in Summary of Product Characteristics.
- Contraindications for expectant management of preeclampsia such as an immediate indication for delivery according to SFOG guidelines for preeclampsia (https://www.sfog.se/media/338533/pe-riktlinje-230214.pdf).
- Type 1 diabetes mellitus.
- Current use of metformin.
- Known or suspected allergies against metformin.
- Reluctance or language difficulties that result in difficulty understanding the meaning of study participation.
- Unable to understand the informed consent process.
- Previous participation in the study.
- Established fetal compromise that necessitates imminent delivery (including planned delivery after 48 hours of corticosteroid treatment). This will be decided by the clinical team before expectant management is offered to the patient.
- Suspicion of a major known fetal anomaly or malformation.
- Renal disease or dysfunction, suggested by a creatinine level greater than or equal to 125 µmol/L or rapidly declining renal function.
- Known acute or chronic metabolic acidosis, including diabetic ketoacidosis.
- Not suitable for inclusion by the opinion of the investigator.

### Exclusion criteria

Exclusion criteria include contraindications to treatment with metformin, type 1 diabetes mellitus, current use of metformin or known/suspected allergy to metformin, language difficulties or inability to understand the informed consent process, previous participation in the study, established fetal compromise that necessitates imminent delivery, suspicion of a major known fetal anomaly or malformation, renal disease or dysfunction, known acute or chronic metabolic acidosis, including diabetic ketoacidosis, or not suitable for inclusion by the opinion of the investigator. A complete list of exclusion criteria is provided in box 1.

### Participant enrolment

Research personnel identify potential participants and inform them. Inclusion and randomisation to metformin ER or placebo are performed by qualified trial physicians. Strategies for recruitment include daily screening of relevant clinical wards, providing information to ward staff, and posting trial information in waiting rooms and outpatient clinics. Information videos are also available in multiple languages. At satellite sites, research nurses at the main site handle enrolment in collaboration with appointed medical doctors at the satellite site.

### Randomisation and allocation concealment

Participants are randomised in a 1:1 ratio to receive either one gram of metformin ER (Merck Healthcare KGaA, Darmstadt, Germany), three times daily, or an identical placebo tablet, from randomisation until birth. Random permuted blocks of varying sizes are used, stratified by trial site and gestational age group at inclusion (22+0 to 28+6 and 29+0 to 33+6 weeks). The randomisation list is prepared by an independent statistician and incorporated into the web-based electronic case report form (eCRF) in the REDCap database. All treatment packs are identical and contain either an active drug or a placebo. Trial participants, investigators, clinicians, and researchers are all blinded to treatment allocation. If unblinding is required, the responsible clinician is informed about treatment allocation. In accordance with Swedish regulations, sealed envelopes with the treatment code prepared by the trial statistician are provided by the distributor and stored with the trial medication at each site. The monitor ensures allocation concealment.

### Intervention

The dose is based on pharmacokinetic studies showing that a daily dose of 3 g of metformin ER administered to women with preterm pre-eclampsia resulted in plasma concentrations similar to those observed in non-pregnant individuals taking 2 g daily.^8 24^ The results do not suggest metformin accumulation with the three-gram dose.^8^ A daily dose of 3 g was also used in the PI2 trial.^8^

Participants in Pre-eclampsia Intervention 4 (PI4) begin treatment with one 500 mg tablet three times daily. If well tolerated, the dose is increased on day two to a maximum of two tablets three times daily until birth. Each participant keeps a treatment diary (digital or paper), in which the number of tablets taken and any side effects are documented. In cases of gastroenteritis or dehydration, participants are instructed to stop treatment and contact the research nurse regarding dose adjustment. To promote adherence and detect adverse events (AEs), the research nurse contacts the participant at least twice weekly. The research nurse monitors the data entered from the treatment diary for drug adherence and after birth, the research nurse counts remaining tablets. Trial medication (both active drug and placebo) is donated by Merck Healthcare KGaA, Darmstadt, Germany. It is delivered in bulk to Apoteket Produktion & Laboratorier AB (APL) for packing and labelling in accordance with a computer-generated randomisation list. Labelling and preparation are carried out in accordance with the requirements of the Medicines for Human Use (Clinical Trials) regulations. Apoteket AB Clinical Trial Unit in Uppsala, Sweden (January 2024 to January 2026), and Tamro AB, Gothenburg, Sweden (January 2026 onwards), handle the storage and distribution of drugs to trial sites.

### Co-interventions

Participation in this trial will not alter or interfere with treatment or care routinely given for pre-eclampsia. Standard clinical care is maintained throughout the trial according to Nordic guidelines^25–27^ and are adhered to by all participating centres to ensure similar management across trial sites. Routine clinical care includes analyses of biochemical markers, blood pressure monitoring, and surveillance for indications of delivery (see online supplemental appendix 2). Ongoing management, such as the use of corticosteroids, antihypertensive agents and magnesium sulphate, is administered according to local protocols. The decision to deliver is left to the treating clinician.

### Outcomes

The primary outcome is prolongation of gestation, defined as the time in hours from randomisation to birth. Secondary outcomes include length of stay in neonatal care (hours from birth to 12 AM at day of discharge) and neonatal birth weight (g). Neonatal care includes both hospital-based and home-based care, with the latter defined as neonatal inpatient care delivered in the neonate’s home environment. Exploratory outcomes are detailed in box 2, and the overall trial schedule is presented in table 1. Validity and reliability of study instruments are summarised in online supplemental appendix 3. The eCRF is provided in online supplemental appendix 4.

Box 2 **Exploratory objectives and outcomes**

**Exploratory\ objectives are to examine whether 3 g of metformin ER daily compared with placebo can:**

- Reduce maternal and neonatal adverse outcomes including
  - Maternal death.
  - Eclampsia.
  - Cerebral vascular event.
  - Posterior reversible encephalopathy syndrome.
  - Pulmonary oedema.
  - Left ventricular failure.
  - Renal dialysis.
  - Renal failure.
  - HELLP (haemolysis, elevated liver enzymes and low platelets) syndrome.
  - Thrombocytopenia (platelets<100×10^9^/L).
  - Liver transaminases>500 IU/L.
  - Liver haematoma or rupture.
  - Admission to an intensive care unit.
  - Severe hypertension (systolic blood pressure of 160 mm Hg or a diastolic blood pressure of ≥110 mm Hg).
  - Disseminated intravascular coagulation.
  - Caesarean section.
  - Major postpartum haemorrhage.
  - Thromboembolic disease.
  - Mother’s length of stay in hospital postpartum.
  - Number of antihypertensive drugs at discharge after delivery.
  - Duration of antihypertensive medication.
  - Intrauterine fetal demise.
  - Neonatal death.
  - Growth restriction at birth (defined as less than 2 SD from median weight from Swedish birth weight growth curves).
  - Grade 3 or 4 intraventricular haemorrhage.
  - Necrotising enterocolitis.
  - Reversed a-wave in the ductus venosus.
  - Persistent reversed flow in the umbilical artery.
  - Redistribution in the middle cerebral artery.
  - Low Apgar score, defined as less than 4 at 5 min.
  - Umbilical artery pH below 7.1.
  - Invasive ventilation, intubation or mechanical ventilation or non-invasive ventilation; continuous positive airway pressure (CPAP) support or high flow nasal oxygen.
  - Grade III/IV hyaline membrane disease.
  - Surfactant use.
  - Retinopathy of prematurity.
  - Neonatal sepsis.
- Be proven tolerable and safe, and that women remain compliant by measurement of
  - Nausea.
  - Vomiting.
  - Diarrhoea.
  - Headache.
  - Lactic acidosis.
  - Drug adherence.
- Reduce circulating concentrations of the pre-eclampsia-related biomarkers
  - Soluble fms-like tyrosine kinase-1.
  - Placental growth factor.
  - Soluble endoglin.
- Affect the gut and vaginal microbiome composition and produce biologically active metabolites.
- Improve or at least not decrease patient-reported experience measures, including
  - Breastfeeding initiation.
  - Depressive symptoms.
  - Self-esteem.
  - Perceived stress.
  - Breastfeeding self-efficacy.
  - Health-related quality of life.
  - Parent-infant bonding.
- Be cost-effective
  - Short-term follow-up: Health outcomes will be compared with direct costs for prescribed drugs dispensed through outpatient pharmacies and costs for healthcare use during the mother’s initial treatment and pregnancy period, during birth, and for mother and child until discharge obtained from administrative cost data from the local hospital registers, as well as productivity loss resulting from sick leave and parental leave calculated based on questionnaire data.
  - Long-term follow-up: Health outcomes will be compared with direct costs for prescribed drugs dispensed through outpatient pharmacies and costs for healthcare use during the mother’s initial treatment and pregnancy period, up until 2 years postpartum obtained from register data, as well as register data on social insurance transactions.
- Lead to improved cognitive development of the child measured by
  - Ages and Stages Questionnaire, Third Edition (ASQ) 24-month questionnaire.
  - Modified Checklist for Autism in Toddlers, Revised (M-CHAT-R).
  - Bayley Scales of Infant Development-4 from the Swedish Neonatal Quality Register (SNQ).
  - Data related to social, behavioural, cognitive and motor development from the Swedish Neonatal Quality Register (SNQ).

**Table 1:** Trial schedule for enrolment, interventions and assessments

PI4	Screening/randomisation	After 5 days of treatment±1 day	Recurrent visits two times weekly	Birth	Discharge visit	2–3 months follow-up	2 years follow-up
In/exclusion criteria	A B C						
Informed consent	A^*^ B^*^ C^*^			A^†^ B^‡^ C^†^			
Patient information and characteristics	A B C					A B C	A B C
Questionnaires for mother	A B C					A B C	A B C
Questionnaires for child							A B C
Registry data/Medical records for child							A B C
Drug instruction	A B C						
Biological samples for the mother	A^§^ ^¶ **^ B^§,¶^ C^§^ ^¶ **^	A^**^ C^**^	A^§^ B^§^ ^††^ C^§ ††^	A^§^ ^‡‡^ B^§,^ C^§^		A^§^ ^¶^ C^§ ¶^	A^§^ ^¶^ **C^§^ ^¶ **^
Placental samples				A C			
Cord blood sample				A B C			
Weight, height and body mass index of the child							A B C
Blood analyses in clinical routine	A B C		A B C	A B C	A B C	A B C	
Tolerability, safety and adherence/drug compliance			A B C	A B C			
Incidents (AE and SAE)			A B C	A B C	A B C	A B C	
Discontinue trial medication				A B C			
Primary and secondary outcomes				A B C	A B C	A B C	
Exploratory outcomes				A B C	A B C	A B C	A B C
Cost data							A B C

A Procedures in Swedish sites.

B Procedures in Finnish sites.

C Procedures in Norwegian sites.

* Informed consent for the mother.

† Informed consent regarding the child.

‡ Finland obtains full consent at enrolment.

§ Blood sample.

¶ Urine sample.

** Rectal and vaginal swabs.

†† At one occasion.

‡‡ Cerebrospinal fluid sample.

AE, adverse event; PI4, Pre-eclampsia Intervention 4; SAE, serious adverse event.

### Sample collection

All outcome data are collected by research personnel. When possible, biological samples are obtained at inclusion, twice weekly during treatment, at birth and during post-natal follow-up. These samples include blood, urine, self-administered rectal and vaginal swabs, placenta, cord blood and cerebrospinal fluid (if collected during routinely placed regional anaesthesia for caesarean section). All biological specimens in Sweden are stored in regional biobanks for future analyses. In Finland and Norway, samples are stored locally until the end of the trial, when they are shipped to the sponsor in Sweden for storage in a local biobank hosted by the University of Gothenburg. The biological samples collected in Finland are exempt from the Finnish Biobank Act (688/2012) as they are collected exclusively for analysis within the scope of this specific trial and will be immediately destroyed following analysis.

### Sample size calculation

The assumptions for the sample size calculation are based on non-public internal data from our observational cohort of women with preterm pre-eclampsia in Sweden, the GO PROVE biobank; further details about GO PROVE are provided in the study protocol.^28^ In this cohort, the median duration of pregnancy prolongation was 7 days (mean 10 days), with a coefficient of variation of 1.35. In this population, 5% of women reached 37+0 weeks’ gestation.

A total of 294 participants will be recruited to the PI4 trial to detect a 50% relative increase in prolongation of gestation between the metformin ER and placebo arms, corresponding to an absolute increase of 3.5 days in median prolongation under the assumption of a median prolongation of 7 days in the placebo arm. This calculation assumes 90% power, a two-sided test with a 5% significance level and a log-normal distribution for the outcome. Sample size calculations were performed using SAS/STAT Software, V.9.4 (SAS Institute, Cary, North Carolina, USA).

### Quality assurance and trial safety

Data are entered by research personnel and participants into the eCRF using the web-based REDCap system provided by the Sponsor (Region Västra Götaland). Two-factor authentication is required to enter the system. All data are pseudonymised and each participant is assigned a unique participant study identification number in REDCap. The code list is kept in closed lockers within the hospital’s physically secure perimeter. The final database will be stored on a secure server at the University of Gothenburg.

Pseudonymised information may be shared with researchers at other universities and other research contexts within and outside the Nordic countries. In cases where data are transferred to a country outside the EU/EEA, the transfer is preceded by a careful assessment and supported by specific provisions under the General Data Protection Regulation and the Public Access and Secrecy Act.

The sponsor representative is responsible for maintaining quality assurance throughout the trial. This includes the design, implementation, data collection, evaluation, reporting and archiving of the trial, to ensure compliance with the protocol, Good Clinical Practice (GCP), organisational requirements, Clinical Trials Regulation EU 536/2014 and other applicable regulatory standards.

Quality assurance procedures include training trial personnel, using working manuals, holding regular meetings, central monitoring and conducting audits. No interim analyses are planned.

Agreements with external hospitals are established to collect follow-up data if a participant is referred to another healthcare facility.

### Data monitoring and audits

The trial is monitored by an independent monitor as per the trial’s monitoring plan. Monitoring ensures that the participants’ rights, safety and well-being are protected and that data in the eCRF are complete, correct and consistent with the source data. Authorised representatives of the sponsor and Competent Authorities may conduct audits or inspections at the trial site, including source data verification.

### Data and safety monitoring board

The data and safety monitoring board (DSMB) is composed of three external members, including an obstetrician, a paediatrician and a statistician, who meet every 12 months. The DSMB monitors participant safety through periodic review of AEs and serious AEs, including masked safety summaries by treatment group, and makes recommendations to the sponsor regarding the continuation, modification or termination of the trial. The trial may be prematurely terminated on DSMB recommendation if emerging safety data indicate that continuation of the trial is not in the best interests of participants. No formal interim analyses for efficacy or futility are planned, and no sample size re-estimation will be performed. The DSMB will not review unblinded efficacy data.

### Adverse events

Information on AEs is documented in REDCap and reported to Merck Healthcare KGaA, Darmstadt, Germany and the DSMB on a quarterly basis and to the Swedish Medical Products Agency annually. All serious adverse events (SAEs) are reported to Merck Healthcare KGaA, Darmstadt, Germany within one working day after they are reported in REDCap, to the DSMB quarterly (maternal deaths are reported within one working day), and to the Swedish Medical Products Agency annually. Suspected unexpected serious adverse reactions are reported to the Swedish Medical Products Agency within 7 days in case of death and within 15 days in other cases.

### Deviations and serious breaches

Any serious breaches or deviations from the trial protocol, GCP and other regulations that might affect the participant’s safety and integrity, or the reliability and robustness of the trial data, are reported to the sponsor. After assessment, the sponsor reports any breaches or deviations to the Swedish Medical Products Agency within 7 days.

### Statistical methods

Descriptive data will be presented as mean and SD, geometric mean and coefficient of variation (CV) or median and IQR, as appropriate for numeric variables. Categorical variables will be summarised as counts and percentages. All baseline variables will be tabulated and presented descriptively by randomisation group.

Statistical analyses will be conducted according to the intention-to-treat principle, with all randomised participants analysed according to their randomised treatment allocation. The primary outcome, prolongation of gestation, will be analysed using a log-normal mixed-effects censored regression model, and treatment effects will be presented as geometric mean ratios with 95% CIs. Planned induction at 37+0 weeks’ gestation without another maternal or fetal indication for delivery will be treated as right-censoring. In cases of intrauterine fetal demise, the event time will be treated as interval-censored between the last assessment confirming fetal viability and the diagnosis of fetal demise. Neonatal length of stay will be analysed using a Fine-Gray subdistribution hazards model, with stillbirth and neonatal death treated as competing events, and treatment effects will be presented as subdistribution HRs with 95% CIs. Neonatal birth weight will be analysed using a log-normal mixed-effects model, and treatment effects will be presented as geometric mean ratios with 95% CIs.

All primary and secondary efficacy analyses will be adjusted for the stratification variables (gestational age group at randomisation and trial site). Additional prognostic factors may be included as prespecified covariates in the Statistical Analysis Plan (SAP), based on findings from the observational GO PROVE study. Trial site will be included as a random effect in mixed-effects models and as a fixed effect in the Fine-Gray model. Safety analyses will be conducted on the safety population, defined as all randomised participants who received at least one dose of the trial medication. In these analyses, participants will be analysed according to treatment received. AEs will be tabulated by treatment group and summarised by severity, duration, relation to treatment and seriousness.

The overall type I error rate for the primary and secondary outcomes will be controlled using a hierarchical testing procedure. If the primary outcome is statistically significant, the full significance level (α=0.05) will be sequentially allocated to the secondary outcomes, in the following prespecified order: neonatal length of stay followed by neonatal birth weight. Statistical significance will be declared for each outcome with a significant result until the first non-significant test is encountered, at which point no further outcomes will be tested for confirmatory purposes.

Missing outcome data are expected to be limited, as the primary and key secondary outcomes are obtained from medical records. The handling of missing data will be prespecified in the SAP, and sensitivity analyses will be conducted where appropriate. Additional per-protocol analyses based on treatment adherence will also be performed.

Detailed specifications of all primary, secondary, exploratory, sensitivity and per-protocol analyses will be provided in the SAP, which will be finalised before database lock and unblinding.

### Health economic analyses

The economic evaluation will include drug use and healthcare costs for mother and child from enrolment and (i) until discharge (short-term follow-up) or (ii) until 2 years postpartum (long-term follow-up), social insurance transactions associated with caring for the child as well as productivity loss associated with sick leave and parental leave. All costs will be estimated from a societal perspective, including those to the health system and families throughout the study period. Discounting, handling of potentially skewed data and sensitivity analyses will be conducted according to state-of-the-art methods.

### Patient and public involvement

We have an established collaboration with the Swedish pre-eclampsia patient association, ‘Preeklampsiföreningen’ (www.preeklampsi.se). Representatives from the association have been involved in the design of the trial and preparation of participant information sheets and other information materials. They have been consulted regarding the patient/population, intervention, comparison and outcomes (PICO) as well as the clinical importance of the selected outcomes, which have resulted in the adjustment of some of the exploratory outcomes. Preeklampsiföreningen has conducted a survey regarding biological samples and what is and is not acceptable to women, leading to amendments in the setup of some of the sampling protocols. Preeklampsiföreningen is distributing information about the trial through their social media channels and in interviews. They will also help disseminate results from the trial in Sweden. They are represented by a co-author in this publication (KS).

### Trial status and duration

The PI4 trial recruitment began on 22 January 2024, and the first participant was enrolled on 6 February 2024 (see Gantt Chart in figure 1). The trial is projected to take 3.5 years, and the 2 year follow-up visits are expected to be completed in December 2029.

## Ethics and dissemination

The trial is conducted in accordance with GCP, as defined by the International Council for Harmonisation. The protocol, including all amendments, was approved by the Swedish Ethical Review Authority and the Swedish Medical Products Agency in accordance with the EU Clinical Trials Regulation (CTR) and Swedish national legislation, via the Clinical Trials Information System (CTIS). Participating sites in Finland and Norway have also obtained approval in CTIS in compliance with CTR and their respective national regulatory frameworks (national regulatory reference numbers: 5.1.1-2023-22720, 5.1.2-2023-81893, 5.1.2-2024-002342, 5.1.2-2024-029871, 5.1.2-2024-096141, 5.1.2-2025-010462, 5.1.2-2025-098308, 26-03-04, FIMEA/2026/001334, 5.1.2-2026-045517). Norway was approved on 22 May 2026; reference numbers are not routinely used by Norwegian authorities). The protocol is registered at ClinicalTrials.gov (NCT06033131) where any protocol modifications will be updated. The current protocol is V.8.0, 29 May 2026.

The principal investigator at each trial site ensures that the participant is given full and adequate oral and written information about the trial, its purpose, any risks and benefits, as well as inclusion and exclusion criteria. Informed consent from the participant before birth and from the legal guardians after birth are obtained by appointed medical trial doctors according to GCP. The second consent concerns follow-up of children and analyses of cord blood and placental samples. If this consent is not granted, cord blood and placental samples will be destroyed and follow-up data for the child will not be analysed. Any possible harm due to participation in the trial is covered by Patient Insurances in respective country.

Results will be disseminated at international conferences and published in peer-reviewed journals. Findings will also be communicated via popular science outlets, media, a patient organisation and the trial website: https://www.preeclampsiaresearch.com/pi-trials. Letters with information on the trial results will be sent to all participants.

## Discussion

Pre-eclampsia is a severe pregnancy-specific disorder associated with substantial maternal and neonatal morbidity and mortality. Identification of an affordable, safe and effective treatment that can slow disease progression has the potential to prolong gestation and mitigate the adverse consequences of preterm birth. Such an intervention could reduce the burden on neonatal intensive care services and lower morbidity and mortality associated with iatrogenic preterm birth.^29^

Several potential pharmacological interventions for the treatment of pre-eclampsia have previously been evaluated in clinical trials.^3–8^ Studies investigating esomeprazole,^3^ pravastatin,^4^ tadalafil^5^ and antithrombin^6^ demonstrated no significant prolongation of gestation among women with pre-eclampsia. One trial showed that sildenafil citrate prolonged pregnancy by approximately 4 days compared with placebo^7^; however, subsequent evidence indicated an increased risk of pulmonary hypertension and neonatal mortality associated with sildenafil exposure.^30^ Consequently, sildenafil is unlikely to remain a viable therapeutic candidate for the treatment of pre-eclampsia.

Since the only promising results from clinical trials related to potential treatment for pre-eclampsia are reported on metformin from the PI2 trial,^8^ confirmation of these findings in high-resource settings and in larger, more diverse populations is of great importance.

In addition to the PI2 trial, there is also evidence of the efficacy of metformin against the development of pre-eclampsia from meta-analyses focusing on diabetes or obesity.^31 32^ In these reports, metformin intake was associated with a reduction in pregnancy-induced hypertension,^31 32^ and a tendency towards a reduced risk of developing pre-eclampsia.^32^ However, this was not confirmed in a Swedish observational register-based study.^33^

There are also pre-clinical studies of metformin in the context of pre-eclampsia, exploring potential mechanisms of action.^16 17 34 35^ Metformin seems to operate through various processes including vasodilation, angiogenesis,^16^ inhibition of inflammatory damage and oxidative stress responses.^34^ In addition, it suppresses inflammation of trophoblast cells in vitro.^35^ Recent studies also indicate that metformin acts through pathways in the gut,^18^ which suggest that gut microbial changes might contribute to the effects of metformin.^19–21^ Importantly, we collect an extended biobank in PI4 to investigate these hypotheses further.

Metformin has been shown to be safe for the fetus in numerous human epidemiological studies when administered in the first, second and third trimesters of pregnancy.^9–15^ Reassuringly, no SAEs related to the trial medications were observed in the PI2 trial.^8^

The PI4 trial is an academic-led multicentre clinical trial and will complement the PI2 and PI3 trials with a high-resource setting and a multicentre approach, including 14 main sites and 4 satellite sites. The primary endpoint, prolongation of gestation, was chosen after consensus in the steering committee including input from the Swedish Preeclampsia Patient Association as a clinically relevant and pragmatic outcome. This outcome enables a well-powered trial in this relatively rare condition while being an important determinant of neonatal outcomes. The trial is powered to detect a 50% relative increase in prolongation of gestation, corresponding to an absolute increase of 3.5 days assuming a median prolongation of seven days in the placebo arm. Previous studies demonstrate a continuous improvement in neonatal outcomes with advancing gestational age, suggesting that even modest prolongation of pregnancy of one or a few days may translate into clinically meaningful benefits in terms of survival, neonatal morbidity and duration of hospitalisation.^36 37^

The trial design with an intention-to-treat approach will generate high internal validity and high-quality evidence for the potential effect of the investigational drug. The multicentre approach was chosen due to the disorder’s rarity and the generalisability of the results. The included trial sites vary in size and geographical location, increasing generalisability. In contrast to PI2 and PI3 where participants are followed up 6 weeks postpartum, in PI4 mothers and children are followed up both at 2-3 months postpartum and 2 years postpartum corrected age with focus on the cognitive development of children.

In the ongoing PI3 trial, 500 women from a South African population will be enrolled to confirm the findings from PI2 and to investigate the effects on birth weight and length of the neonatal hospital stay.^23^ A fourth multicentre trial for metformin in preterm pre-eclampsia, PI NL, started in September 2025 in the Netherlands (Clinicaltrials.gov: NCT06452498). Women in pregnancies in gestational weeks 23+0 to 31+6 will be included, and the primary endpoint is the same as in this trial: prolongation of gestation. Outcomes in all these studies have been kept similar to generate a higher level of evidence.

Together, these four metformin trials (PI2, PI3, PI4 and PI-NL) will provide an opportunity to evaluate the effects of metformin on the primary, secondary and selected exploratory outcomes in individual participant data meta-analyses. This collaborative approach will increase the precision of treatment effect estimates and strengthen the evidence base for maternal and neonatal outcomes, particularly for outcomes that are not adequately powered within individual trials. Results from these analyses may provide important evidence to inform future clinical guidelines for the management of preterm pre-eclampsia.

Preterm pre-eclampsia is a serious condition associated with substantial maternal and neonatal morbidity and mortality. A therapeutic intervention capable of slowing disease progression has the potential to improve survival and reduce short- and long-term complications for both mothers and their infants. Metformin is considered safe for use during pregnancy and accumulating preclinical and clinical evidence suggests it may represent a promising therapeutic option for the management of preterm pre-eclampsia. The clinical relevance of the PI4 trial is therefore considerable. If metformin is shown to effectively delay disease progression, its implementation could lead to meaningful improvements in maternal and offspring health outcomes, ultimately benefiting thousands of families.

## Supplementary material

10.1136/bmjopen-2026-120993 online supplemental file 1

10.1136/bmjopen-2026-120993 online supplemental file 2

10.1136/bmjopen-2026-120993 online supplemental file 3

10.1136/bmjopen-2026-120993 online supplemental file 4

## Data Availability

No data are available.
