# Supplementary material for: Can metformin prolong pregnancy in preterm pre-eclampsia? Study protocol for a Nordic multicentre randomised placebo-controlled trial (PI4)
Source: BMJ Open. 2026 Oct 13;16(10):e120993. doi: 10.1136/bmjopen-2026-120993 (PMC13663727; doi:10.1136/bmjopen-2026-120993)
Supplement: Supplementary file 1 [file bmjopen-16-10-s001.pdf]

CLINICAL TRIAL PROTOCOL

Preeclampsia Intervention 4 – A double blind phase III randomised controlled trial assessing metformin to prolong gestation in preterm preeclampsia

Preeclampsia Intervention 4 (PI 4)

Trial ID:	PI 4
EU Trial number:	2022-502707-29-00
Version number:	Version 8.0
Date:	2026-05-29
Sponsor:	Västra Götalandsregionen
Sponsor representative:	[redacted]
Co-sponsor:	University of Gothenburg
Co-sponsor representative:	[redacted]

Table Of Contents

Revision history 5
Signature page 14
Contact information 27
List of used acronyms and abbreviations 31
1. Synopsis 33
2. Background and rationale 36
3. Benefit-risk evaluation 39
4. Trial objectives 44
4.1. Primary objective 44
4.2. Secondary objectives 44
4.3. Exploratory objectives 44
4.4. Primary endpoint 46
4.5. Secondary endpoints 46
4.6. Exploratory endpoints 46
5. Trial design and procedures 46
5.1. Overall trial design 46
5.2. Procedures and flow chart 48
5.3. Biological sampling procedures 52
5.3.1. Handling, storage, and destruction of biological samples 52
5.3.2. Total volume of blood per subject 52
5.3.3. Biobank 53
5.4. Procedures for obtaining patient reported experience measures 53
5.5. Follow up in Nordic registries up to two years post partum (Part B) 54
5.5.1. Procedures for obtaining cost data 54
5.6. Start, end, temporary halt and early termination 55
5.6.1 Start of the clinical trial 55
5.6.2 Temporary halt or early termination 55
5.6.3 End of the clinical trial 56
6. Subject selection 56
6.1. Inclusion criteria 56
6.2. Exclusion criteria 56
6.3. Screening and inclusion 57

6.4. Withdrawal criteria 57
7. Trial treatments 58
7.1. Description of the investigational medicinal product 58
7.2. Storage of trial medications 58
7.3. Dose and administration 58
7.4. Packaging, labelling, and handling of investigational medicinal products(s) 58
7.5. Drug accountability and treatment compliance 59
7.6. Randomisation 59
7.7. Blinding 59
7.8. Code breaking 59
7.9. Auxiliary medicinal products 60
7.10. Concomitant use of other medicinal products and treatments 60
7.10.1. Recommendations and precautions related to Metformin 60
7.10.2. Combinations requiring precautions 60
7.11. Destruction 60
7.12. Treatment after trial end 61
8. Methods for measurement of endpoints for clinical efficacy and safety 61
8.1. Primary endpoint 61
8.2. Secondary endpoints 61
8.3. Exploratory endpoints 61
9. Handling of Adverse Events 63
9.1. Definitions 63
9.1.1. Adverse Event (AE) 63
9.1.2. Serious Adverse Event (SAE) 64
9.1.3. Suspected Unexpected Serious Adverse Reaction (SUSAR) 65
9.1.5. Adverse Evenst of Special Interest (AESI) 65
9.2. Assessment of Adverse Events (AE) 65
9.2.1. Assessment of causal relationship 65
9.2.2. Assessment of intensity 66
9.2.3. Assessment of seriousness 66
9.3. Reporting and registration of Adverse Events 66
9.3.1. Reporting of Adverse Events (AE) 67
9.3.2. Reporting of Serious Adverse Events (SAE) 67
9.3.3. Reporting of Suspected Unexpected Serious Adverse Reactions (SUSAR) 67

9.3.4 Reporting of Adverse Event of Special Interest (AESI) 67
9.4. Follow-up of Adverse Events 68
9.5. Independent Data Monitoring Committee 68
9.6. Annual Safety Report (ASR) 68
9.7. Procedures in case of emergencies or overdose 68
9.8. Reference Safety Information 68
10. Statistics 68
10.1. Analysis populations 68
10.2. Statistical methods 69
10.3. Adjustment of significance and confidence interval 71
10.4. Sample size calculations 72
10.5. Interim analysis (if relevant) 72
11. Quality Control and Quality Assurance 72
11.1. Quality Assurance and Sponsor oversight 72
11.2. Monitoring 72
11.3. Source data 73
11.4. Deviations, serious breaches and other reporting obligations 73
11.5. Audits and inspections 73
12. Ethics 74
12.1. Compliance to the protocol, ICH-GCP and regulations 74
12.2. Ethical review of the trial 74
12.3. Procedure for obtaining informed consent 74
12.4. Data protection 74
12.5. Insurances 75
13. Substantial changes to the trial 75
14. Data governance 75
14.1. Case Report Form 76
15. Notification of trial completion, reporting, and publication 76
16. References 77
17. Attachments 79

Revision history

**Protocol version**	**Summary of changes (pages in version without track changes)**<br>*Describe all changes since the first final protocol.*
Version 3.0	*Front page and Header*<br>Version (3.0) and date (2023-09-11) updated
Version 3.0	*Table of Contents*<br>Updated
Version 3.0	*Contact information*<br>Page 12: Contact information to Monitor added<br>Page 14: A local researcher is added
Version 3.0	*1. Synopsis, page 16*<br>EU Trail number added and version and date updated
Version 3.0	*2. Background and rationale*<br>*Biological role of metformin as possible therapy, page 21*<br>• Text added related to the effect of Metformin on gut microbiome
Version 3.0	*4. Study objectives*<br>*4.3 Exploratory objectives, page 30*<br>• Added objective related to gut and vaginal microbiome composition<br>• Changed wording for clarification: “Improve or at least not decrease patient-reported experience measures including”<br>• Word added for clarification:Breastfeeding “initiation”<br>• Added patient-reported experience measures:<br>- “Depressive symptoms”<br>- “Self-esteem”<br>- “Perceived stress”<br>- “Breastfeeding self-efficacy”<br>- “Health-related quality of life”<br>- “Parental-infant bonding”
Version 3.0	*5.2 Procedures and flow chart*<br>*Inclusion*<br>• Page 31: Inclusion value for albumin/creatinine ratio changed to ≥8<br>• Page 32: Number of questionnaires are changed from three to “four” and text regarding the treatment diary is changed due to inclusion of the online version MyCap<br>*Table 1. Flowchart of the trial*<br>• Page 33: Column namned “After 5 days of treatment +- 1 day” added between “Randomisation visit” and “Recurrent visits two times weekly +- 1 day”<br>• Page 34:“Rectal and vaginal swabs” added in biological samples for the mother in the columns “Randomisation visit” and “After 5 days of treatment +- 1 day”
Version 3.0	*5.3 Biological sampling procedures*<br>*5.3.1 Handling, storage, and destruction of biological samples, page 35*<br>• Text added regarding administration of rectal and vaginal swabs
Version 3.0	*5.4 Procedures for obtaining patient reported experience measures, page 36*<br>• A sentence regarding administration and translation of questionnaires is added

	• Experience measure questionnaires “health-related quality of life (EQ-5D)” and “Postpartum Bonding Questionnaire (PBQ)” are added in the text<br>• Change of wording in the sentence “At the first follow up (2-3 months postpartum)…” (“the first” is deleted)<br>• A sentence regarding question about what group women think they belonged to is added
Version 3.0	*7. Study treatments*<br>*7.3. Dose and administration, page 40*<br>• Text added regarding treatment diary alternatives<br>*7.5. Drug accountability and treatment compliance, page 40*<br>• Sentence added regarding treatment diary alternatives
Version 3.0	*8. Trial endpoints*<br>*8.3 Exploratory endpoints, page 44*<br>• Endpoint related to effects of Metformin on gut and vaginal microbiome composition added<br>*8.3 Exploratory endpoints, page 43*<br>*Patient-reported experience measures, including*<br>• The word “self-efficacy” is added after breastfeeding<br>• “EQ-5D (numeric) at inclusion and 2-3 months follow-up” and “Postpartum Bonding Questionnaire (numeric) at 2-3 months follow-up” are added
Version 3.0	*12. Ethics*<br>*12.4 Data protection, page 56*<br>• *A sentence regarding the MyCap application is added and text regarding REDCap is updated*
Version 3.0	*16. References*<br>• Page 57-59: References are updated in the protocol text and in the reference list
Version 3.0	*17. Attachments*<br>*Appendix D, page 59*<br>• “Health-related quality of life (EQ-5D)” and “Postpartum Bonding Questionnaire (PBQ)” are added
Version 4.0	*Front page and Header*<br>Version (4.0) and date (2023-12-20) are updated
Version 4.0	*Table of Contents*<br>Updated
Version 4.0	*Signature page*<br>Page 10: Local PI is changed
Version 4.0	*Contact information*<br>Page 13: E-mail address for [redacted] is changed<br>Page 14: Local Principal Investigators: Local PI for Uppsala University hospital is changed<br>Page 15-16: Local Researchers are updated
Version 4.0	*Synopsis*<br>*Exclusion criteria*<br>• Page 20: List of Concomitant medications that are not compatible with metformin is deleted due to updated information from the Swedish Medical Products Agency.
Version 4.0	*4. Study objectives*<br>*4.3 Exploratory objectives*<br>Page 31: Objectives related to cognitive development of the child and longterm reproductive maternal health are added

Version 4.0	*5. Study design and procedures*<br>*5.1.Overall study design*<br>• Page 31-32: The following sentence is added regarding the structure of the study: "The study is divided in Part A (from start to two-three months follow up and Code breaking) and Part B (Follow up beyond primary and secondary outcomes including two year follow up of participant and child and follow up in Swedish registries up to sixteen years post partum)."<br>• Figure 1, page 32: Trial diagram is updated<br>• Paragraph numbers are updated<br><br>*5.2 Procedures and flow chart*<br>*2-3 months follow up visit postpartum, page 34*<br>• "2-3" is changed to "two-three" in the headline and the text<br>• A paragraph named "Two year follow up visit postpartum (Part B)" is added<br>*Table 1, page 35:* Flowchart is updated<br><br>*5.3.Biological sampling procedures*<br>• Page 36-37: "Part B" is added in parentheses where suitable<br><br>*5.4 Procedures for obtaining patient reported experience measures*<br>• Page 37-38: "Part B" and information regarding included questionnaires are added where suitable<br><br>*Page 38*<br>• *Paragraphs "5.5.Follow up in Swedish registries up to 16 years postpartum (Part B)," and "5.5.1 Reproductive maternal health at long-term" are added*<br>• *5.5.2. Procedures for obtaining cost data*<br>○ *Page 38-39: The section is updated with information regarding long term follow up of women and children*
Version 4.0	*6.0 Subject selection, page 40*<br>*6.2 Exclusion criteria*<br>• List of Concomitant medications that are not compatible with metformin is deleted due to updated information from the Swedish Medical Products Agency.
Version 4.0	*7. Study treatments*<br>*7.10. Concomitant use of other medicinal products and treatments, page 50*<br>• The sections "7.10.1. Recommendations and precautions related to Metformin" and "7.10.2. Combinations requiring precautions" are added due to updated information from the Swedish Medical Products Agency
Version 4.0	*9. Handling of Adverse Events*<br>*9.1.Definitions*<br>• Page 48: The section "9.1.5.Adverse Evenst of Special Interest (AESI)" is added<br>9.1.1. Adverse Event (AE)<br>• Page 47: A section regarding exceptions that are not considered AEs is added<br>*9.3. Reporting and registration of Adverse Events*<br>• Page 51: The section "9.3.4. Reporting of Adverse Event of Special Interest (AESI)" is added
Version 4.0	*10. Statistics*<br>*10.2. Statistical methods, page 52-56*<br>• The section is updated with information regarding analyses related to stillborn babies and babies that die during neonatal care<br>Minor changes related to rewording has been done in the section
Version 5.0	*Front page and Header*<br>Version (5.0) and date (2024-03-04) are updated

Version 5.0	*Table of Contents*<br>Updated
Version 5.0	*Synopsis*<br>*Trial ID, page 18*<br>• Version (5.0) and date (2024-03-04) are updated<br>*Trial design, page 20*<br>• The following senctence is added regarding follow up:"The study also include follow up of the child's neurocogitive function and follow up in Swedish registries up to two years post partum."<br>*Trial period, page 22*<br>• Period is updated to: Q1 2024 – Q3 2029
Version 5.0	*4. Study objectives*<br>*4.3 Exploratory objectives*<br>• Page 32: Objective related longterm reproductive maternal health is deleted
Version 5.0	*5. Study design and procedures*<br>*5.1.Overall study design*<br>• Page 32: The following sentence is updated regarding the the time line of the study: "The study is divided in Part A (from start to two-three months follow up and Code breaking) and Part B (Follow up beyond primary and secondary outcomes including two year follow up of participant and child and follow up in Swedish registries up to two years post partum)."<br>• Figure 1, page 33: Trial diagram is updated<br>• Paragraph numbers are updated<br>*5.2 Procedures and flow chart*<br>*Inclusion, page 35*<br>• A sentence regarding timing of consent is updated: "A qualified trial physician, with GCP training will get informed consent post partum, after answering all questions the legal guardian or gardians have."<br>• The following sentence is deleted: "Primarily, this consent will be obtained simultaneously as the consent for the trial."<br>• *Table 1, page 36: Flowchart is updated*<br>*5.5."Follow up in Swedish registries up to 16 years postpartum (Part B)",* page 39 is changed to *"Follow up in Swedish registries up to two years postpartum (Part B)"*<br>• *"5.5.1 Reproductive maternal health at long-term",* page 39, is deleted<br>• *5.5.1. Procedures for obtaining cost data, page 39-40:* The section is updated regarding the time line for long term follow up of women and children<br>*5.6 End of Study, page 40*<br>• The section is updated according to the new definition of end of study: "after the two-year follow up visits of all participants are completed"
Version 5.0	*12. Ethics*<br>*12.3 Procedures for obtaining informed consent, page 59*<br>• The following sentence is added at the end of the section: "Informed consent from the legal guardian or guardians regarding follow up data of the child and potential analyses of the umbilical cord sample taken at delivery will be obtained after the child is born."
Version 5.1<br>(no substantial modifications)	*Front page and Header*<br>• Version (5.1) and date (2024-04-10) are updated
Version 5.1<br>(no substantial modifications)	*Table of Contents*<br>• *Updated*
Version 5.1<br>(no substantial modifications)	*Synopsis*<br>*Trial ID, page 23*<br>• Version (5.1) and date (2024-04-10) are updated
Version 5.1<br>(no substantial modifications)	*9. Handling of Adverse Events*<br>*9.1.2. Adverse reaction (AR), page 48*<br>• The paragraph is deleted

	9.3 Reporting and registration of Adverse Events, page 51<br>• The sentence is deleted "Adverse drug reactions will be reported to Merck as described in the agreement between Merck and the sponsor"
Version 6.0	*Front page and Header*<br>• Version (6.0) and date (2025-01-23) are updated
Version 6.0	*Table of Contents*<br>• *Updated*
Version 6.0	*Signature page, page 15 and 16*<br>• Signature pages for Danderyd and Linköping are added
Version 6.0	*Contact information*<br>*Local Principal Investigators, page 18*<br>• [redacted] are added<br>*Steering committee, page 19*<br>• [redacted] is deleted<br>*Responsible researcher for follow up of the children, page 19-20*<br>• [redacted] are added<br>*Local researchers, page 20*<br>• Affiliation for [redacted] is added<br>• [redacted] is deleted<br>• [redacted] are added
Version 6.0	*Synopsis*<br>*Trial ID, page 23*<br>Version (6.0) and date (2025-01-23) are updated
Version 6.0	*5.0 Study design and procedures*<br>*5.2 Procedures and flow chart*<br>*Two year follow up visit postpartum (Part B), page 39*<br>• The following sentences are updated or added: "The follow up visit will take place at 2 years corrected age", "Biological samples will also be obtained, as well as blood pressure.The participant will also be asked questions about her current health and any prescribed medication.", and "To assess the risk of autism spectrum disorders, the Modified Checklist for Autism in Toddlers, Revised (M-CHAT-R) will be used. In addition, data two years post partum from the Swedish Neonatal Quality Register (SNQ) on Bayley Scales of Infant Development-4 and other variables related to social, behavioral, cognitive and motor development will be analyzed for children born before gestational week 28 or with a birthweight of ≤ 3 SD. Retrieved data will be validated in hospital records."<br>*5.4 Procedures for obtaining patient reported experience measures, page 42*<br>• The following sentence is added: "Questions regarding feeding the baby, breast milk /formula, will also be included."<br>• The following sentence is a deleted: "Individual qualitative interviews about women's experiences participating in the trial will be conducted approximately at two months after childbirth with a strategic sample of participants."
Version 6.0	*8.0 Trial endpoints*<br>*8.3 Exploratory endpoints, page 52*<br>• The following sentence is deleted: "Qualitative open-question individual interviews with 15-25 participants conducted approximately 2-3 months after discharge from hospital".
Version 6.0	*9. Handling of Adverse Events*<br>*9.1.3. Serious Adverse Event (SAE) , page 53*<br>• The following sentence is deleted under exceptions to SAE: "Intrauterine fetal demise which, according to the Swedish Pregnancy Register, is expected to occur in 1.6% of pregnancies in preterm preeclampsia."
Version 6.0	*17.0 Attachments*<br>*Attachment D, page 67*<br>• The forms "Breastfeeding Questions" and M-CHAT-R are added

Version 7.0	The complete document is updated according to the ICH GCP E6(R3) protocol template and in line with the Nordic setting ("Swedish" is changed to "Nordic, and Norway/Norwegian and Finland/Finnish is added when appropriate")
Version 7.0	*Front page and Header*<br>Version (7.0) and date (2025-11-01) are updated
Version 7.0	*Signature pages*<br>• "Sweden" is added to PI affiliations, page 13-19<br>• "Västra Götalandregionen" is changed to "Region Västra Götaland", page 13<br>• PIs for Umeå, Norway and Finland are added, page 20-25
Version 7.0	*Contact information*<br>*Local Principal Investigators, page 27-28*<br>• [redacted] are added<br>*Local researchers, page 29-30*<br>• [redacted] are added
Version 7.0	*List of used acronyms and abbreviations*<br>• The list is updated in line with the Nordic setting, page 30-31
Version 7.0	*Synopsis*<br>*Trial ID*<br>• Version (7.0) and date (2025-11-01) are updated, page 32<br>*Trial design*<br>• The following sentence is added: "Follow up data up to two years post partum will be retrieved from Swedish and Norwegian registries and from Swedish, Norwegian and Finnish medical records.", page 33<br>*Exclusion criteria*<br>• Links to Finnish and Norwegian guidelines are added, page 34<br>*Intervention*<br>• Links to Finnish and Norwegian guidelines are added, page 34-35<br>*Trial period*<br>• "Q3 2029" is changed to "Q4 2029", page 35
Version 7.0	*3. Benefit-risk evaluation*<br>*Pharmacokinetics in preterm preeclampsia*<br>• The following text is deleted "…and pregnant women taking a daily dose of 2 grams", page 42
Version 7.0	*5. Trial design and procedures*<br>*5.1 Overall trial design*<br>• The following sentences are added: "Fourteen Nordic main sites (predominately University Hospitals) participate in the trial. Norway will have a National PI according to local regulations, which is not required in Sweden and Finland. To facilitate management of participants that are moved to neighbouring hospitals after inclusion and to increase inclusion rates, a decentralised approach will also be applied. Four smaller hospitals in Sweden within the catchment area of their respective main centre will be included as satellite sites in the trial (see Attachment G). Site agreements are established between sponsor and every site.", "Follow up beyond primary and secondary outcomes including two year follow up of participant and child in Swedish and Norwegian registries and Swedish, Norwegian and Finnish medical record", and "Type of biological samples taken differ between countries (see Table 1).", page 45-46<br>• Figure 1, Trial diagram, is updated: "Swedish" is deleted under Part B, and "End of Study" is changed to "Q4 2029", page 47<br>*5.2 Procedures and Flow chart*<br>Inclusion<br>• The following text is added: "…main centres or at satellite centres within the catchment area", page 47<br>• Links to Finnish and Norwegian guidelines are added, page 48<br>Two year follow up visit postpartum (Part B)<br>• Norwegian registers are added, page 49

	• The following sentence is added: "Follow up data for children up to two years post partum will also be retrieved from Swedish, Norwegian and Finnish medical records.", page 49<br>Table 1. Flowchart of the trial<br>• The chart is updated in line with the Nordic setting, page 50<br>*5.3 Biological sampling procedures*<br>5.3.1 Handling, storage, and destruction of biological samples<br>• The following sentence is added: "The biological samples collected in Finland are exempt from the Finnish Biobank Act (688/2012) as they are collected exclusively for analysis within the scope of this specific trial, and will be immediately destroyed following analysis. Consequently, the samples will not be stored for future, unspecified biobank research.", page 51<br>5.3.3 Biobank<br>• The following sentence is added "Biological samples taken in Finnish sites will be handled and stored in Hospital laboratories and shipped to the sponsor in Sweden at the end of the trial according to Material Transfer Agreements (MTAs). Biological samples taken in Norwegian sites will be handled and stored in a project specific biobank at Oslo University Hospital Rikshospitalet ([redacted]) and shipped to the sponsor in Sweden at the end of the trial according to Material Transfer Agreements (MTAs). The samples will be stored in a freezer at the University of Gothenburg run by the sponsor. After finalization of the trial, samples will be analysed for biomarkers related to preeclampsia. After all analyses are performed, samples will be destroyed.", page 51<br>*5.5 Follow up in Nordic registries up to two years post partum (Part B)*<br>5.5.1 Procedures in obtaining cost data<br>• The following sentence is added: "Cost data will be retrieved for the Swedish and Norwegian populations from registers and medical records and for the Finnish population from medical records only.", page 53<br>• Norwegian registers are added, page 53-54<br>*5.6 Start, end, temporary halt and early termination*<br>5.6.1 Start of the clinical trial<br>• This section and the following text is added according to the updated protocol template: " The start of the trial is defined as recruitment of the first participant, i.e. February 6 2024. Sponsor will report the start of the trial in each participating country through notification in the CTIS within 15 days.", page 54<br>5.6.3 End of the clinical trial<br>• This section and the following text is added according to the updated protocol template: "The study ends after the two-year follow up visits of all participants are completed.", page 55
Version 7.0	*7. Trial treatments*<br>*7.2 Storage and trial medications*<br>• Distributor is changed from "Apoteket AB Clinical Trial Unit" to "Tamro AB", page 57<br>*7.4 Packaging, labelling, and handling of investigational medicinal products(s)*<br>• Distributor is changed from "Apoteket AB Clinical Trial Unit" to "Tamro AB", page 57<br>*7.7 Blinding*<br>• The following sentence is added: "Folded leaflet with information in Swedish, Finnish, Norwegian and Danish will be used for labelling on cans and boxes", page 58<br>*7.8 Code breaking*<br>• "Apoteket" is changed to "Tamro", page 58<br>*7.11 Destruction*<br>• The following sentence is updated "All medication including unused trial medications from Swedish sites will be destroyed by Tamro AB, including unused trial medications, and from Norwegian and Finnish sites by Hospital pharmacies", page 59
Version 7.0	*9. Handling of Adverse Events*<br>*9.3.3 Reporting of Suspected Unexpected Serious Adverse Reactions (SUSAR)*

	• The following sentence is deleted: "Those SAE which are assessed by sponsor to be SUSAR are reported via a CIOMS form to the EudraVigilance database / Swedish Medical Products Agency according to the specified time frames.", page 66<br>• The following text is deleted: "…we therefore ask the Swedish Medical Product Agency for help. SUSARs will be reported via the which will be sent to registrator@lakemedelsverket.se by Eudralink (not email since it contains personal data) or by regular mail.", page 66<br>• The following text is added: "…reporting of SUSARs for all Nordic sites will be handled by Department of Clinical Pharmacology at Sahlgrenska University hospital, Sweden." Page 66<br>*9.3.4 Reporting of Adverse Event of Special Interest (AESI)*<br>• The following text is deleted: "…from local PIs to sponsor within 24 hours. If the AESI is fatal or life-threatening it will be reported by the sponsor to Merck and the DSMB within 1 working day and to the EudraVigilance database / Swedish Medical Products Agency as soon as possible and no later than 7 days after the adverse event has become known to the sponsor. Other AESI are reported as soon as possible and no later than 15 days after they have come to the sponsor's knowledge.", page 66<br>• The following text is added: "…as a SUSAR according to 9.3.3", page 66
Version 7.0	*9.4-9.8:* numbers are updated, page 66-67
Version 7.0	*12 Ethics*<br>*12.1 Compliance to the protocol, ICH-GCP and regulations*<br>• The version of GCP is updated to "ICH-GCP E6(R3)", page 73<br>*12.5 Insurances*<br>• The following sentence is added: "In Finland trial participants are insured via patient insurance and regarding metformin via Finnish pharmaceutical insurance. In Norway trial participants are insured through the Norwegian Drug Liability Insurance Association (LAF).", page 74
Version 7.0	*17 Attachments*<br>*Attachment C*<br>• "Norwegian" is added, page 78<br>*Attachment G*<br>• Added attachement (Satellite sites), page 78
Version 7.1<br>(no substantial modifications)	*4 Trial Objectives*<br>*4.5 Secondary endpoints*<br>• The definition of length of neonatal care is updated in the following sentence: Length of stay in neonatal care, defined as hours from birth to 12 AM at day of discharge from neonatal care, including both hospital and home-based care, page 46
Version 7.1<br>(no substantial modifications)	*5 Trial design and procedures*<br>*5.2 Procedures and flow chart*<br>Follow up visits during pregnancy<br>The following sentence is clarified regarding that PHASE 20 only will be used at Swedish sites: "In Swedish sites, safety and tolerability will be measured by assessing tolerability according to the PHASE-20 questionnaire (Attachment B)", page 49<br><br>Two year follow up visit postpartum (Part B)<br>The following sentence is added to clarify the timing for the two year follow up visit: "Participants will be invited to the two year follow up visit between 23+0 and 25.5 months post birth corrected age. However, the follow up visits might occur after this timeframe if needed", page 50
Version 7.1<br>(no substantial modifications)	*6 Subject selection*<br>*6.1 Inclusion criteria*<br>To promote consistency across sites, a standardized definition of viability is added at page: 56<br>• "There is a live fetus when assessed with ultrasound where the prognosis is considered such that the fetus can survive a birth with interventions from neonatal care

	• If the pregnancy is over 23 weeks of gestation, it has been decided to deliver by caesarean section if there is an indication for delivery (except for spontaneous start of labour)<br>• There must be a decision for active fetal monitoring, up to 24+0 weeks with ausculation of fetal heartbeat and over 24+0 weeks with CTG.<br>• All eligible women in PI4 with pregnancies 22+0-24+0 ,or in case of uncertainty about viability regardless of gestational week, must be discussed with the sponsor ([redacted]) before inclusion*”*
Version 7.1<br>(no substantial modifications)	*7 Trial treatments*<br>*7.1 Description of the investigational medicinal product*<br>Attachment D is changed to E, page 58<br><br>*7.3 Dose and administration*<br>The following sentence is added: “Dosage procedures and treatment responsibility for Finland are outlined in Attachment H”, page 58
Version 7.1<br>(no substantial modifications)	*8 Methods for measurement of endpoints for clinical efficacy and safety*<br>*8.2 Secondary endpoints*<br>The definition of length of neonatal care is updated in the following sentence: “Length of neonatal care, defined as hours from birth to 12 AM at day of discharge from neonatal care, including both hospital and home-based care”, page 61
Version 7.1<br>(no substantial modifications)	*17. Attachements*<br>*Attachement D*<br>The questionnaire “The Ages and Stages Questionnaire, Third Edition (ASQ) 24-month questionnaire” is added, page 79<br>*Attachment H*<br>In this new attachment, dosage procedures and treatment responsibility for Finland are outlined, page 79
Version 8.0	*Front page and Header*<br>Version (8.0) and date (2025-05-29) are updated
Version 8.0	*Signature page*<br>[redacted], PI for Danderyd has been changed to [redacted], page 19
Version 8.0	*Contact information*<br>*Local Principal Investigators, page 28*<br>• [redacted] as PI for Danderyd<br>*Responsible researcher for follow up of the children, page 30*<br>• “PhD”, has been added to titles for [redacted]<br>*Local researchers*<br>• [redacted] has been deleted, page 30<br>• “PhD”, has been added to titles for [redacted], page 31

Signature page

Sponsor

I am responsible for ensuring that this protocol includes all essential information to be able to conduct this trial. I will submit the protocol and all other important trial-related information to the responsible investigator(s) so that they can conduct the trial correctly. I am aware that it is my responsibility to hold the staff members who work with this trial informed and trained.

Signature of sponsor´s representative Date

[redacted] PI and local PI Sahlgrenska University Hospital, Region Västra Götaland, Sweden

Principal Investigator

I have read this protocol and agree that it includes all essential information to be able to conduct the trial. By signing my name below, I agree to conduct the trial in compliance with this clinical trial protocol, the EU Regulation on clinical trials of medicinal products for human use (EU 536/2014), the Declaration of Helsinki, ICH-GCP (Good Clinical Practice) guidelines and the current national regulations governing the conduct of this clinical trial.

I will submit this protocol and all other important trial-related information to the staff members and investigators who participate in this trial, so that they can conduct the trial correctly. I am aware of my responsibility to continuously keep the staff members and investigators who work with this trial informed and trained.

I am aware that quality control of this trial will be performed in the form of monitoring and eventual audit and inspection.

____

Principal Investigator's signature Date

____

[redacted], local PI, Falu Lasarett, Dalarna Region, Sweden

Principal Investigator

I have read this protocol and agree that it includes all essential information to be able to conduct the trial. By signing my name below, I agree to conduct the trial in compliance with this clinical trial protocol, the EU Regulation on clinical trials of medicinal products for human use (EU 536/2014), the Declaration of Helsinki, ICH-GCP (Good Clinical Practice) guidelines and the current national regulations governing the conduct of this clinical trial.

I will submit this protocol and all other important trial-related information to the staff members and investigators who participate in this trial, so that they can conduct the trial correctly. I am aware of my responsibility to continuously keep the staff members and investigators who work with this trial informed and trained.

I am aware that quality control of this trial will be performed in the form of monitoring and eventual audit and inspection.

____
Principal Investigator’s signature Date

____
[redacted], local PI, Uppsala University Hospital, Uppsala Region, Sweden

Principal Investigator

I have read this protocol and agree that it includes all essential information to be able to conduct the trial. By signing my name below, I agree to conduct the trial in compliance with this clinical trial protocol, the EU Regulation on clinical trials of medicinal products for human use (EU 536/2014), the Declaration of Helsinki, ICH-GCP (Good Clinical Practice) guidelines and the current national regulations governing the conduct of this clinical trial.

I will submit this protocol and all other important trial-related information to the staff members and investigators who participate in this trial, so that they can conduct the trial correctly. I am aware of my responsibility to continuously keep the staff members and investigators who work with this trial informed and trained.

I am aware that quality control of this trial will be performed in the form of monitoring and eventual audit and inspection.

____
Principal Investigator's signature Date

____
[redacted], local PI, Karolinska Huddinge and Karolinska Solna, Stockholm Region, Sweden

Principal Investigator

I have read this protocol and agree that it includes all essential information to be able to conduct the trial. By signing my name below, I agree to conduct the trial in compliance with this clinical trial protocol, the EU Regulation on clinical trials of medicinal products for human use (EU 536/2014), the Declaration of Helsinki, ICH-GCP (Good Clinical Practice) guidelines and the current national regulations governing the conduct of this clinical trial.

I will submit this protocol and all other important trial-related information to the staff members and investigators who participate in this trial, so that they can conduct the trial correctly. I am aware of my responsibility to continuously keep the staff members and investigators who work with this trial informed and trained.

I am aware that quality control of this trial will be performed in the form of monitoring and eventual audit and inspection.

____

Principal Investigator's signature Date

____

[redacted], local PI, Malmö University Hospital and Lund University hospital, Skåne Region, Sweden

Principal Investigator

I have read this protocol and agree that it includes all essential information to be able to conduct the trial. By signing my name below, I agree to conduct the trial in compliance with this clinical trial protocol, the EU Regulation on clinical trials of medicinal products for human use (EU 536/2014), the Declaration of Helsinki, ICH-GCP (Good Clinical Practice) guidelines and the current national regulations governing the conduct of this clinical trial.

I will submit this protocol and all other important trial-related information to the staff members and investigators who participate in this trial, so that they can conduct the trial correctly. I am aware of my responsibility to continuously keep the staff members and investigators who work with this trial informed and trained.

I am aware that quality control of this trial will be performed in the form of monitoring and eventual audit and inspection.

Principal Investigator's signature Date

[redacted], local PI, Danderyd Hospital, Stockholm Region, Sweden

Principal Investigator

I have read this protocol and agree that it includes all essential information to be able to conduct the trial. By signing my name below, I agree to conduct the trial in compliance with this clinical trial protocol, the EU Regulation on clinical trials of medicinal products for human use (EU 536/2014), the Declaration of Helsinki, ICH-GCP (Good Clinical Practice) guidelines and the current national regulations governing the conduct of this clinical trial.

I will submit this protocol and all other important trial-related information to the staff members and investigators who participate in this trial, so that they can conduct the trial correctly. I am aware of my responsibility to continuously keep the staff members and investigators who work with this trial informed and trained.

I am aware that quality control of this trial will be performed in the form of monitoring and eventual audit and inspection.

____

Principal Investigator's signature Date

____

[redacted], local PI, University Hospital Linköping, Östergötland Region, Sweden

Principal Investigator

I have read this protocol and agree that it includes all essential information to be able to conduct the trial. By signing my name below, I agree to conduct the trial in compliance with this clinical trial protocol, the EU Regulation on clinical trials of medicinal products for human use (EU 536/2014), the Declaration of Helsinki, ICH-GCP (Good Clinical Practice) guidelines and the current national regulations governing the conduct of this clinical trial.

I will submit this protocol and all other important trial-related information to the staff members and investigators who participate in this trial, so that they can conduct the trial correctly. I am aware of my responsibility to continuously keep the staff members and investigators who work with this trial informed and trained.

I am aware that quality control of this trial will be performed in the form of monitoring and eventual audit and inspection.

Principal Investigator's signature Date

[redacted], local PI, University Hospital of Umeå, Västerbotten Region, Sweden

Principal Investigator

I have read this protocol and agree that it includes all essential information to be able to conduct the trial. By signing my name below, I agree to conduct the trial in compliance with this clinical trial protocol, the EU Regulation on clinical trials of medicinal products for human use (EU 536/2014), the Declaration of Helsinki, ICH-GCP (Good Clinical Practice) guidelines and the current national regulations governing the conduct of this clinical trial.

I will submit this protocol and all other important trial-related information to the staff members and investigators who participate in this trial, so that they can conduct the trial correctly. I am aware of my responsibility to continuously keep the staff members and investigators who work with this trial informed and trained.

I am aware that quality control of this trial will be performed in the form of monitoring and eventual audit and inspection.

Principal Investigator's signature   Date

[redacted], local PI, Tampere University Hospital, Wellbeing Services County of Pirkanmaa, Finland

Principal Investigator

I have read this protocol and agree that it includes all essential information to be able to conduct the trial. By signing my name below, I agree to conduct the trial in compliance with this clinical trial protocol, the EU Regulation on clinical trials of medicinal products for human use (EU 536/2014), the Declaration of Helsinki, ICH-GCP (Good Clinical Practice) guidelines and the current national regulations governing the conduct of this clinical trial.

I will submit this protocol and all other important trial-related information to the staff members and investigators who participate in this trial, so that they can conduct the trial correctly. I am aware of my responsibility to continuously keep the staff members and investigators who work with this trial informed and trained.

I am aware that quality control of this trial will be performed in the form of monitoring and eventual audit and inspection.

____

Principal Investigator’s signature Date

____

[redacted], local PI, Helsinki University Hospital, HUS region, Finland

Principal Investigator

I have read this protocol and agree that it includes all essential information to be able to conduct the trial. By signing my name below, I agree to conduct the trial in compliance with this clinical trial protocol, the EU Regulation on clinical trials of medicinal products for human use (EU 536/2014), the Declaration of Helsinki, ICH-GCP (Good Clinical Practice) guidelines and the current national regulations governing the conduct of this clinical trial.

I will submit this protocol and all other important trial-related information to the staff members and investigators who participate in this trial, so that they can conduct the trial correctly. I am aware of my responsibility to continuously keep the staff members and investigators who work with this trial informed and trained.

I am aware that quality control of this trial will be performed in the form of monitoring and eventual audit and inspection.

____

Principal Investigator's signature Date

____

[redacted], National PI Norway

Principal Investigator

I have read this protocol and agree that it includes all essential information to be able to conduct the trial. By signing my name below, I agree to conduct the trial in compliance with this clinical trial protocol, the EU Regulation on clinical trials of medicinal products for human use (EU 536/2014), the Declaration of Helsinki, ICH-GCP (Good Clinical Practice) guidelines and the current national regulations governing the conduct of this clinical trial.

I will submit this protocol and all other important trial-related information to the staff members and investigators who participate in this trial, so that they can conduct the trial correctly. I am aware of my responsibility to continuously keep the staff members and investigators who work with this trial informed and trained.

I am aware that quality control of this trial will be performed in the form of monitoring and eventual audit and inspection.

____

Principal Investigator's signature Date

____

[redacted], local PI Oslo University Hospital, Norway

Principal Investigator

I have read this protocol and agree that it includes all essential information to be able to conduct the trial. By signing my name below, I agree to conduct the trial in compliance with this clinical trial protocol, the EU Regulation on clinical trials of medicinal products for human use (EU 536/2014), the Declaration of Helsinki, ICH-GCP (Good Clinical Practice) guidelines and the current national regulations governing the conduct of this clinical trial.

I will submit this protocol and all other important trial-related information to the staff members and investigators who participate in this trial, so that they can conduct the trial correctly. I am aware of my responsibility to continuously keep the staff members and investigators who work with this trial informed and trained.

I am aware that quality control of this trial will be performed in the form of monitoring and eventual audit and inspection.

Principal Investigator's signature Date

[redacted], local PI Akershus University Hospital, Norway

Contact information

**Responsibility on the clinical trial**	
Sponsor representative<br>Responsibility:<br>• *Responsible for all aspects of the study including contact with authorities*	[redacted], senior consultant, associate professor, Region Västra Götaland<br><br>VO kvinnosjukvård, obstetrik<br>Diagnosvägen 14<br>Sahlgrenska University Hospital<br>416 85 Göteborg<br><br>[redacted]<br><br>[redacted]
Co-sponsor representative<br><br>Responsibility:<br>• *Responsible for all aspects of the study*	[redacted], senior consultant, associate professor, University of Gothenburg<br><br>Diagnosvägen 14<br>Sahlgrenska University Hospital<br>416 85 Gothenburg<br><br>[redacted]<br><br>[redacted]
Principal investigator<br><br>Responsibility:<br>• *Responsible fro all aspects of the study*	[redacted], senior consultant, associate professor<br><br>VO kvinnosjukvård, obstetrik<br>Diagnosvägen 14<br>Sahlgrenska University Hospital<br>416 85 Gothenburg<br><br>[redacted]<br><br>[redacted]
Monitor organization<br>• Responsibility:*Initiation visits and monitoring visits during the trial according to Monitor plan*	[redacted], RNM Monitor<br>VO Kvinnosjukvård, obstetrik<br>Diagnosvägen 15<br>Sahlgrenska University Hospital<br>416 85 Göteborg
Data management	[redacted], Data Manager<br>Gothia Forum, Sahlgrenska University Hospital

**Responsibility on the clinical trial**	
Responsibility:<br>• Establish a data management plan<br>• Establish and manage the ecrf	su.sdstab.redcap@vgregion.se
Statistics<br>Responsibility:<br>• *Statistical analysis*	[redacted], Ph.Lic. biostatistician, Statistical Consulting Group, Gothenburg, Chalmers University of Technology and University of Gothenburg
Local Principal investigators<br>Responsibility:<br>• *Locally responsible for the conduct of the trial at the respective sites*	[redacted], senior consultant, associate professor, Sahlgrenska University Hospital<br><br>[redacted], senior consultant, PhD, Uppsala University Hospital<br><br>[redacted], senior consultant, associate professor, Falun Regional Hospital<br><br>[redacted], senior consultant, associate professor, Karolinska University Hospital<br><br>[redacted], MD, associate professor, Skåne University Hospital<br><br>[redacted], consultant, PhD student, Danderyd Hospital<br><br>[redacted], senior consultant, associate professor, University Hospital Linköping<br><br>[redacted], consultant, PhD, Umeå University Hospital<br><br>[redacted], senior consultant, professor, Tampere University Hospital<br><br>[redacted], consultant, associate professor Helsinki University Hospital<br><br>[redacted], Senior Consultant, Professor, National PI Norway, Oslo University Hospital

**Responsibility on the clinical trial**	
	[redacted], Senior Consultant, Professor,Local PI Oslo University Hospital<br><br>[redacted], Senior Consultant, PhD, Akershus University Hospital
Steering committee<br><br>Responsibility:<br><br>*Managing the trial*	[redacted], senior consultant, associate professor, Sahlgrenska University Hospital<br><br>[redacted], senior consultant, associate professor, Stellenbosch University<br><br>[redacted], senior consultant, professor, University of Melbourne<br><br>[redacted], senior consultant, professor, University of Melbourne<br><br>[redacted], senior consultant, associate professor, Falun Regional Hospital<br><br>[redacted], senior consultant, associate professor, Karolinska University Hospital
Responsible researcher for patient experience measures<br><br>Responsibility:<br><br>• *Planning and conducting sub-investigations for patient centered outcomes*	[redacted], midwife, associate professor, University of Gothenburg
Responsible researchers for economic evaluation<br><br>Responsibility:<br><br>• *Planning and conducting the economic evaluation of the trial*	[redacted], pharmacist, associate professor, University of Gothenburg

**Responsibility on the clinical trial**	
Responsible researcher for follow up of the children<br><br>Responsibility:<br><br>• *Planning and conducting the two year follow up of the children*	[redacted], senior consultant neonatology, associate professor, Skåne University Hospital<br><br>[redacted], senior consultant, professor Pediatric Ophthalmology, Queen Silvia Children's Hospital at Sahlgrenska University Hospital<br><br>[redacted], senior consultant neonatology, PhD, Queen Silvia Children's Hospital at Sahlgrenska University Hospital<br><br>[redacted], chartered psychologist neuropsykologi, Queen Silvia Children's Hospital at Sahlgrenska University Hospital
Patient organization<br><br>Responsibility:<br><br>• *Contributing with patient perspective on the protocol set up and subject information and consent form*	[redacted], patient representative from the Swedish Preeclampsia patient organization
Local researchers<br><br>Responsibility:<br><br>• *Information and support for clinical staff.*<br>• *Inclusion of participants in the trial*	[redacted], senior consultant, PhD, Sahlgrenska University Hospital<br><br>[redacted], senior consultant, PhD, Sahlgrenska University Hospital<br><br>[redacted], senior consultant, PhD Skåne University Hospital<br><br>[redacted], MD, associate professor, Karolinska University Hospital<br><br>[redacted], senior consultant, professor, University Hospital Linköping<br><br>[redacted], senior consultant, PhD, Umeå University Hospital<br><br>[redacted], consultant, Umeå University Hospital

**Responsibility on the clinical trial**	
	[redacted], consultant, PhD, Tampere University Hospital<br><br>[redacted], consultant, Södra Älvsborg Hospital<br><br>[redacted], senior consultant, Norra Älvsborg Hospital<br><br>[redacted], senior consultant, Skaraborg Hospital Skövde<br><br>[redacted], MD, PhD, Västmanland Hospital Västerås

List of used acronyms and abbreviations

**Abbreviation**	**Term/Explanation**
Adverse Event (AE)	Any untoward medical occurrence in a subject to whom a medicinal product is administered and which does not necessarily have a causal relationship with this treatment.
Serious Adverse Event (SAE)	Any untoward medical occurrence that at any dose requires inpatient hospitalisation or prolongation of existing hospitalisation, results in persistent or significant disability or incapacity, results in a congenital anomaly or birth defect, is life-threatening, or results in death
AR	Adverse Reaction = any unfavourable and unexpected reaction to an investigational medicinal product, regardless of dose
ASR	Annual Safety Report = the annual safety report for reporting to authorities. In Sweden this is the Swedish Medical Products Agency.
CI	Confidence Interval
CRF	Case Report Form
CTIS	Clinical Trial Information System = Centralized EU database/portal for application and communication with authorities concerning clinical trials. This includes the Medical Products Agencies and the Ethical Review Authority of all participating countries

CTR	EU Regulation 536/2014, also called CTR, Clinical Trials Regulation
DSUR	Development Safety Update Report = the standard which should be used for annual safety reporting to authorities
ER	Extended Release
FASS	Farmaceutiska specialiteter i Sverige
GCP	Good Clinical Practice
GMR	Geometric Mean Ratio
IB	Investigator's Brochure
ICH	International Council for Harmonization
ITT	Intention-to-treat = including all data from all subjects who have participated in the trial
PI2	Preeclampsia Intervention 2 Trial
PP	Per Protocol analysis = including only data from subjects who have completed the trial completely in accordance with the protocol, with no deviations from the protocol
RSI	Reference safety information. A list of all known adverse reactions for the investigational medicinal product, including severity and frequency of the adverse reaction. The RSI is contained in the Summary of Product Characteristics or IB and is used to determine which new adverse reactions should be reported as suspected unexpected serious adverse reactions (SUSARs).
SPC or SmPC	Summary of Product Characteristics
SUSAR	Suspected Unexpected Serious Adverse Reaction. This is an event that is likely related to the investigational medicinal product but with unexpected occurrence. An adverse reaction is unexpected if its nature or seriousness is not consistent with the information on the product in the RSI.

1. Synopsis

EU Trial number: 2022-502707-29-00	
Title:	Preeclampsia intervention 4
Trial ID:	PI 4, Version 7.0, 2025-11-01
Short background/ Rationale/Aim:	Preeclampsia is globally responsible for 60,000 maternal deaths per year, and far greater numbers of fetal losses. Preterm preeclampsia is a severe variant with the highest rates of neonatal morbidity and mortality due to iatrogenic preterm birth (clinicians are forced to deliver the baby preterm for maternal or fetal health reasons).<br><br>There is preclinical evidence suggesting metformin, a drug commonly used to treat diabetes in and outside pregnancy, may be able to counter the pathophysiology of preeclampsia, raising the possibility that it could be used to treat the condition.<br><br>Members of our research group have recently shown in the Preeclampsia Intervention 2 trial (PI2) that metformin was able to delay delivery in early preterm preeclampsia. Metformin extended release (ER) was associated with a median 7.6-day prolongation of pregnancy (geometric mean ratio (GMR) 1.39 (95% CI 0.99 to 1.96) P=0.057). We found trends towards increased birthweight (mean difference 110g (95%CI -80 to 300), a decreased length of stay at the neonatal intensive care unit (median difference 12.0 days less; GMR 0.82, 95% CI 0.57 to 1.18) in the metformin ER group. Importantly, while gastrointestinal side effects were common, no serious adverse events related to trial medications were observed.<br><br>The PI 2 trial has shown that metformin may be a disease modifying treatment for preterm preeclampsia. The trial is being repeated in a larger scale in the PI3 trial in South Africa to also assess neonatal outcomes. In the Nordic countries, the demographics of the population are different and expectant management of preeclampsia allows for the women to reach 37 weeks of gestation as opposed to 34 weeks of gestation in the PI2 trial. This trial aims to investigate if metformin can prolong gestation, lower neonatal length of stay and increase birthweight in a Nordic setting.

Primary research question:	Can administering metformin to women with preeclampsia diagnosed between 22+0-33+6 weeks gestation prolong gestation in a Nordic setting?
Secondary research question:	Can administering metformin to women with preeclampsia diagnosed between 22+0-33+6 weeks gestation decrease length of neonatal stay and increase birthweight in a Nordic setting?
Primary endpoint:	Prolongation of gestation from randomisation to delivery, with metformin compared to placebo.
Secondary endpoints:	Length of stay in neonatal care, with metformin compared to placebo.<br><br>Neonatal birthweight, with metformin compared to placebo.
Trial design:	We will perform a double blind randomised controlled trial where women participate from time of diagnosis of preeclampsia to discharge from the hospital. The time will vary depending on the gestational age at enrolment and duration of pregnancy after enrolment but is expected to between 0-28 days. The study also include follow up of the child's neurocogitive function. Follow up data up to two years post partum will be retrieved from Swedish and Norwegian registries and from Swedish, Norwegian and Finnish medical records.
Trial population:	Pregnant women over the age of 18 who present with preeclampsia at a gestation of 22+0 to 33+6 weeks at Nordic hospitals participating in the trial and who are considered stable enough to undergo expectant management will be randomised to orally receive either metformin ER or an identical placebo in divided doses daily.
Number of subjects:	294 (147 in each group)
Inclusion criteria:	• A diagnosis of preeclampsia has been made (as defined in 5.2) by the attending clinician<br>• The managing clinicians have made the assessment to proceed with expectant management.<br>• The subject has given written consent to participate in the study.<br>• The woman must be 18 years of age or older<br>• The gestational age is between 22+0 weeks to 33+6 weeks with a viable fetus<br>• The woman carries a singleton pregnancy

Exclusion criteria:	• Contraindications to treatment with metformin as outlined in SmPC (Attachment E)<br>• Contraindications for expectant management of preeclampsia such as an immediate indication for delivery according to Nordic guidelines for preeclampsia (Swedish: https://www.sfog.se/kunskap/preklampsi/, Finnish: https://www.kaypahoito.fi/sv/gvr00094, Norwegian: https://metodebok.no/emne/K4U6nNQ3/hypertensive-svangerskapskomplikasjoner-og-eklampsi-(2020)/f%C3%B8dselshjelp-(ngf))<br>• Type 1 Diabetes Mellitus<br>• Current use of metformin<br>• Known or suspected allergies against metformin<br>• Reluctance or language difficulties that result in difficulty understanding the meaning of study participation<br>• Unable to understand the informed consent process<br>• Previous participation in the study<br>• Established fetal compromise that necessitates imminent delivery (including planned delivery after 48 hours of corticosteroid treatment). This will be decided by the clinical team before expectant management is offered to the patient.<br>• Suspicion of a major known fetal anomaly or malformation.<br>• Renal disease or dysfunction, suggested by a creatinine level greater than or equal to 125 μmol/L or rapidly declining renal function<br>• Known acute or chronic metabolic acidosis, including diabetic ketoacidosis<br>• Not suitable for inclusion by the opinion of the investigator
Intervention:	Women will be randomised to receive one gram of metformin ER three times a day, or an identical placebo tablet. The treatment will be given from randomisation until delivery.<br><br>Ongoing management and decision to deliver will be left to the discretion of the treating clinicians who will be blinded to treatment group allocation. In the participating Nordic countries, there are national guidelines for preeclampsia management (Swedish: https://www.sfog.se/kunskap/preklampsi/, Finnish: https://www.kaypahoito.fi/sv/gvr00094, Norwegian: https://metodebok.no/emne/K4U6nNQ3/hypertensive-

	svangerskapskomplikasjoner-og-eklampsi-(2020)/f%C3%B8dselshjelp-(ngf)).
Trial medication, dosage, administration:	Metformin ER, one gram three times daily taken orally. Once the participants have been recruited, they will start by taking one 500 mg tablet three times a day. If well tolerated it will be increased day two to a maximum of two tablets three times a day. Treatment will continue until delivery. If there are side effects that are not tolerable, the dose will be decreased and the participant will remain blinded. Each participant will keep a treatment diary and number of tablets taken will be documented by the participant or hospital staff. The study will not alter or interfere with treatment or care routinely given for preterm preeclampsia.
Ethical considerations, risk/benefit:	Pregnant women are a vulnerable population. Preterm preeclampsia is a major cause of maternal, fetal and neonatal morbidity and mortality. Metformin is widely used in pregnancy for the treatment of diabetes and is considered safe in pregnancy. Metformin may be a treatment for preeclampsia and for these reasons we believe that it is ethical to conduct this trial.
Trial period:	*Q1 2024 – Q4 2029*

2. Background and rationale

Preeclampsia affects 5% of all pregnancies and is a leading cause of maternal and perinatal morbidity and mortality.[1] It is responsible for more than 60,000 maternal deaths every year and it is estimated that for every preeclampsia related death there are probably 50-100 other women who experience significant morbidity associated with preeclampsia.[1, 2]

Preeclampsia is a multi-system pregnancy specific disorder characterised by placental malperfusion. The placenta releases soluble factors into the maternal circulation.[1]These factors cause endothelial dysfunction which is characterised by hypertension and other end-organ injury. In its most severe form, it affects the end-organ injuries result in severe morbidity and even death.

Currently, the only treatment available to stop disease progression for preeclampsia is termination/delivery of the fetus and placenta.[1]This poses a difficult clinical dilemma. Clinicians are often forced to deliver early due to maternal indications to prevent major maternal morbidity (i.e. severe maternal organ injury), to arrest disease progression, but in doing so, inflict severe prematurity on the neonate.[1] Neonates delivered preterm are at significant risk of severe disability including cerebral palsy, stroke (intracerebral bleeding), retinopathy of prematurity, chronic lung disease and death.

Recently, members of our team have shown that metformin extended release (ER) could prolong pregnancy in cases of preterm preeclampsia in the South African population (Preeclampsia Intervention 2).[3] If we were able to confirm these findings in a different population of women with preeclampsia, it may result in a change in management for preterm preeclampsia across the world, potentially improving neonatal (and lifelong) health outcomes for thousands of individuals.

Biological role of metformin as possible therapy

Published preclinical data strongly suggests that metformin may be a candidate therapeutic for preeclampsia.[4] Firstly, metformin reduced the placental release of anti-angiogenic factors, namely soluble Fms-like tyrosine kinase 1 (sFlt-1) and soluble Endoglin (sEng), that cause maternal endothelial dysfunction in preeclampsia. Secondly, metformin enhanced vasodilation in vessels isolated from maternal omental arteries obtained at caesarean section and induces angiogenesis.[4]

These characteristics make it a potential therapeutic to treat preeclampsia. Since this report, there have been two further preclinical studies showing that metformin can rescue a preeclampsia phenotype in animal models.[5, 6]

Although metformin is generally considered to mediate its antihyperglycemic effects through specific cellular pathways, recent evidence indicates that it also acts through pathways in the gut. For example, a study comparing metformin formulations with reduced and normal plasma exposure provided evidence that the lower bowel is a major site of action for metformin. [7] Furthermore, recent studies in mice [8] and humans [9, 10] suggest that gut microbial changes contribute to the effects of metformin. Metformin increases gut microbiome-derived butyrate and propionate production. [9, 11] Short-chain fatty acids are not only the primary energy source of epithelial cells of the gut, but they can affect the production of lipids and have been associated with cardiometabolic health. In a more recent paper, we have just shown that metformin dosage was negatively associated with cytokine levels (Stromal cell-derived factor 1 and Macrophage migration inhibitory factor), which have been shown to be elevated in preeclampsia. [12, 13]

Data from systematic reviews and meta-analyses suggest metformin may decrease the incidence of preeclampsia

There have been no trials that have examined whether metformin can prevent preeclampsia, where preeclampsia was the primary outcome. However, there have been meta-analyses of trials examining the potential for metformin to treat other diseases of pregnancy where rates of preeclampsia were reported.

A meta-analysis of trials assessing metformin versus insulin to treat gestational diabetes, published in 2019, reported an incidental reduction in pregnancy induced hypertension among those receiving metformin (risk ratio=0.64; 95% CI 0.44 to 0.95; p=0.03; 5 studies, 1457 women) though there were no differences in rates of preeclampsia (risk ratio=0.89; 95% CI 0.65 to 1.12; p=0.45; 9 studies, 1813 women).[14] Another meta-analysis assessing metformin for prevention of hypertensive disorders of pregnancy in women with gestational diabetes or obesity showed that metformin use (compared with insulin) was associated with a reduced risk of pregnancy-induced hypertension (relative risk 0.56; 95% CI 0.37 to 0.85; 4

studies, 1260 women) and a trend towards a reduced risk of preeclampsia (relative risk 0.83; 95% CI 0.60 to 1.14; 8 studies, 1724 women).[15] There was a decreased risk of any hypertensive disease in pregnancy (relative risk 0.70; 95% CI 0.54 to 0.91; 8 studies, 1457 women).

In trials of obese women, when compared with placebo, metformin use was associated with a non-significant reduction in the risk of preeclampsia (relative risk 0.74; 95%; 95% CI 0.09 to 6.28; 2 studies, 840 women). In women with gestational diabetes, metformin use when compared with glyburide was also associated with a non-significant reduction in risk of any hypertensive disorder in pregnancy (relative risk 0.71; 95% CI, 0.41 to 1.25; 5 studies, 560 women).[15] When metformin was compared to any drug there was a trend towards less preeclampsia in the metformin group (relative risk 0.75; 95% CI 0.52 to 1.09; 11 studies, 2713 women) and a reduction in any hypertensive disorder of pregnancy (relative risk 0.75; 95% CI 0.59 to 0.94; 15 studies, 3120 women). The authors concluded that there was a high probability that metformin use was associated with reduced incidence of hypertensive disorders of pregnancy, when compared with other treatments or placebo but that further trials are needed.[15]

Metformin may be a treatment for preterm preeclampsia, the PI 2 trial

In 2021, the results of the PI 2 trial were published in the British Medical Journal. In this double blind, randomised control trial, 180 women with preterm preeclampsia (gestational age between 26 and 32 weeks) were randomised to receive either metformin ER, a drug commonly used to treat diabetes in pregnancy, or a matched placebo.[3, 16] Metformin ER was associated with a median 7.6-day prolongation of pregnancy (geometric mean ratio (GMR) 1.39, 95% confidence interval (CI) 0.99 to 1.95; P=0.057). There were also trends towards increased birthweight (median difference 110 g; 95% CI -80 to 300), and a decreased length of overall neonatal stay of 12 days (GMR 0.82, 95% CI 0.57 to 1.18). Importantly, no serious adverse events related to trial medications were observed.

A pre-specified per-protocol analysis restricted to participants that continued taking the trial medication at any dose showed a statistically significant difference compared with placebo (median difference of 9.6 days; GMR 1.67; 95% CI 1.15 to 2.42), as was another pre-specified analysis of those who took the full dose of trial medications while they remained pregnant (median difference 11.5 days; GMR 1.85; 95% CI 1.16 to 2.96).[17] This trial suggests that metformin ER can prolong gestation in women with preterm preeclampsia, but further trials are needed.

The Preeclampsia Intervention 3 (PI 3) trial (clinical trial registration number PACTR202104532026017), which has recently started recruitment, is being run in the same population as PI 2 to confirm the findings. This trial will include 500 women (250 in each arm) to confirm findings from PI 2 and to investigate effect on birthweight and length of stay in a neonatal unit, outcomes that were exploratory in PI 2. We still need to investigate if metformin ER works in other populations.

In this application, we want to investigate if metformin can prolong pregnancy in a Nordic population of pregnant women with preeclampsia. In the Nordic setting, expectant management is offered until 37 completed gestational weeks, compared to 34 completed gestational weeks in South Africa. Active management for the fetus is indicated from 22 gestational weeks compared to 27 gestational weeks in South Africa. If PI4 confirms the

findings of PI2, this may result in a change in management for preterm preeclampsia, potentially improving maternal and neonatal health outcomes for thousands of individuals.

Why is it important to find a treatment for preterm preeclampsia?

Finding an affordable and safe treatment that temporizes the disease progression of preeclampsia, thereby delaying delivery to gain gestation, could save the lives of many infants and decrease the hospital burden caused by iatrogenic preterm birth. This could reduce child mortality and improve maternal health, one of The United Nations Sustainable Development Goals.

3. Benefit-risk evaluation

Metformin safety data in human pregnancy

Metformin has been extensively used for the treatment of gestational diabetes in pregnancy and in the first trimester of pregnancy in women living with polycystic ovarian syndrome. Glucophage XR was approved in pregnancy in 2022 as outlined in the SmPC (Attachment E). Metformin was classified as a category B drug (animals have not shown a risk to the fetus but where adequate data in humans are not available) in pregnancy by the Food and Drug Administration (FDA) before the FDA amended the pregnancy labelling rule for prescription drug products. The new FDA labelling includes a summary of risk, a discussion of the data supporting that summary, and relevant information to help health care providers make prescribing decisions and counsel women about the use of drugs during pregnancy. The new risk summary from the FDA for metformin is that “the benefit should outweigh risk”. In Sweden, Metformin is classified as “Group B1” in pregnancy, where there is no evidence of fetal malformation and where animal studies do not indicate harmful effects in pregnancy. Treatment is advised when there is a clear indication such as diabetes in pregnancy.

Metformin is commonly used in pregnancy to treat gestational diabetes and polycystic ovarian syndrome. It has been proven safe for the fetus in numerous studies when used in the first, second and third trimesters of pregnancy.[17-21] A meta-analysis of nine controlled studies with women affected by polycystic ovarian syndrome determined that the rate of major birth defects in women exposed to metformin in the first trimester was not statistically increased compared with the disease-matched control group and concluded that there was no evidence that metformin was associated with an increased risk of major birth defects in women affected by polycystic ovarian syndrome.[22]

Some concern has been raised regarding weight gain later during childhood in the offspring of women treated with metformin.[23] However, this has mainly been among a population of women with diabetes or polycystic ovarian syndrome and following a longer treatment time of metformin. In addition, the results of excessive weight gain in the offspring have been conflicting[17-20, 24] A more recent large epidemiological study concluded that antenatal administration of metformin did not adversely impact on childhood growth and developmental outcomes.[25]

The American College of Obstetricians and Gynaecologists and the American Diabetes Association recommend the use of metformin for the treatment of diabetes in pregnancy[26] Metformin is also incorporated in regional guidelines in the Nordic countries for use in women with diabetes in pregnancy. A large Swedish stepped wedge randomised controlled trial investigating the use of metformin and insulin on large for gestational age infants in women

with pregnancy induced diabetes was recently closed without any obvious severe side effects during the trial.[27]

When used during pregnancy for glycaemic control for women with diabetes, metformin is typically administered for many months. Given the drug is likely to be given for a few weeks in this trial, it will probably be a very safe drug option for pregnancy. Furthermore, the safety/risk profile strongly leans towards safety for this trial given the significant adverse impacts of preterm preeclampsia and preterm birth. Lastly, no serious adverse events arising from metformin were identified in the PI2 trial.

Animal studies of metformin in pregnancy

Reproductive studies have been performed in rats and rabbits, with doses up to 600mg/kg/day and have shown no teratogenic effects which represents dosages of 2-6 times the maximum human daily dose based on body surface calculations. Determination of fetal concentrations showed a partial placental barrier to metformin.[28]

Metformin dosage

Metformin is available as 500mg, 850mg or 1000mg metformin hydrochloride tablets. Extended-release tablets are available as 500mg, 750mg or 1000mg tablets and contain the inactive ingredients sodium carboxymethyl cellulose, hypomellose, and magnesium stearate.

Contraindications for the use of metformin

Metformin is contraindicated for the following

- Renal disease or renal dysfunction (suggested by serum creatinine levels ≥125 umol/L or abnormal creatinine clearance) which may also result from conditions such as cardiovascular collapse (shock), acute myocardial infarction, septicaemia and preeclampsia causing renal failure.
- Known hypersensitivity to metformin hydrochloride.
- Acute or chronic metabolic acidosis, including diabetic ketoacidosis, with or without coma.
- Acute conditions with the potential to alter renal function, e.g., dehydration, severe infection, shock, intravascular administration of iodinated contrast media.
- Acute or chronic disease which may cause tissue hypoxia such as cardiac or respiratory failure, pancreatitis, recent myocardial infarction, shock.
- Hepatic insufficiency, acute alcohol intoxication, alcoholism (acute or chronic).

Warnings and precautions

There is very small risk of lactic acidosis (0.03 cases/1000 patient years) and reported cases have been associated with renal insufficiency which is a contraindication for the use of metformin. Participants should be warned of the following nonspecific symptoms which include malaise, myalgia, respiratory distress, somnolence, and nonspecific abdominal pain and should report these symptoms to their attending physician or call the trial investigators or study nurse at the number provided if they occur. The participant will then receive appropriate care depending on severity of the complication.

If any of these very rare outcomes occur, they will be promptly identified as the women who are recruited in PI4 trial will be intensely monitored (because they have preterm preeclampsia). Biochemistry including renal function is analysed at a minimum of two times weekly according to Nordic guidelines in preeclampsia and renal impairment would be diagnosed promptly.

For a full list of adverse reactions and precautions, this is listed in the SmPC (Attachment E).

Adverse reactions

The most common adverse reactions in adults are diarrhoea, nausea, vomiting, abdominal pain and loss of appetite and taste disturbances. Caution will be exercised as some of these symptoms overlap with those of preeclampsia. All participants will be given an information sheet about the possible side-effects of metformin.

Drug interactions

Information about drug interactions is collected from Farmaceutiska specialiteter i Sverige (FASS) (https://www.fass.se/LIF/product?userType=0&npIId=19651020000013) if not otherwise stated. The full list of interactions can be found in the SmPC (Attachment E).

Furosemide:

Pharmacokinetic parameters of both compounds are affected by co-administration. Furosemide increases the metformin plasma and blood Cmax by 22% and blood AUC by 15%, without any significant change in metformin renal clearance. This diuretic is not given during pregnancy so a drug interaction is unlikely to occur in trial participants.

Nifedipine:

A single-dose, metformin-nifedipine drug interaction study in normal healthy volunteers demonstrated that co-administration of nifedipine increased plasma metformin Cmax and AUC by 20% and 9%, respectively, and increased the amount excreted in the urine. This small increase will not require any dose adjustment of metformin. Tmax and half-life were unaffected. Nifedipine appears to enhance the absorption of metformin. Metformin had minimal effects on nifedipine.

Cationic drugs:

Cationic drugs (e.g., amiloride, digoxin, morphine, procainamide, quinidine, quinine, ranitidine, triamterene, trimethoprim, or vancomycin) that are eliminated by renal tubular secretion theoretically have the potential for interaction with metformin by competing for common renal tubular transport systems. Such interaction between metformin and oral cimetidine has been observed in normal healthy volunteers in both single- and multiple-dose, metformin-cimetidine drug interaction studies, with a 60% increase in peak metformin plasma and whole blood concentrations and a 40% increase in plasma and whole blood metformin AUC. There was no change in elimination half-life in the single-dose study. Metformin had no effect on cimetidine pharmacokinetics.

Dolutegravir:

Dolutegravir is an integrase strand inhibitor licensed for use in HIV infection. It is used in a fixed highly active anti-retroviral therapy (HAART) combination called TLD (Tenofovir Disoproxil Fumurate (TDF), Lamivudine (3TC) and Dolutegravir (DTG)) that consists of TDF, 3TC and DTG.[29] TLD is used as a first line treatment to treatment to treat HIV/AIDS in some cases. In pharmacokinetic studies, dolutegravir increases metformin plasma exposure, possibly by organic cation transporter 2 (OCT2) inhibitions.[29] In normal healthy volunteers demonstrated that co-administration of dolutegravir increased plasma metformin Cmax and AUC by 66% and 79%, respectively. For this reason, if a potential participant is taking TDF, she will be limited to a maximum dose of 1500 mg Metformin XR daily (500 mg three times a day).[29]

Rifampicin:

Rifampicin is used to treat tuberculosis. Pharmacokinetic studies have shown that metformin exposure is increased when rifampicin is co-administered with rifampicin but that it did not result in a statistically significant change in the glucose-lowering effect of metformin. Renal clearance and tubular secretion of metformin were unaffected by rifampicin, and it is hypothesized that the increased exposure is due to increased absorption. Because of the increased exposure we will limit women who are taking rifampicin to a maximum dose of 1500 mg Metformin XR daily (500 mg three times a day).[30]

Mechanism of action

Metformin is an antihyperglycemic agent which improves glucose tolerance in patients with type 2 diabetes, lowering both basal and postprandial plasma glucose. Metformin decreases hepatic glucose production, decreases intestinal absorption of glucose, and improves insulin sensitivity by increasing peripheral glucose uptake and utilization. It does not cause hypoglycaemia and hyperinsulinemia.

Please refer to the above sections for the proposed mechanism of action of metformin to counter the pathophysiology of preeclampsia.

Pharmacokinetics

Information about pharmacokinetics is summarized below.

Absorption: Following a single oral dose of metformin ER 500 mg peak plasma levels (Cmax) are achieved with a median value of 7 hours. Following a single oral dose of 1500 mg of metformin ER 750 mg, a mean plasma concentration of 1193 ng/ml is achieved after a median value of 5 hours (range of 4 to 12 hours). Metformin ER 750 mg was shown to be bioequivalent to metformin ER 500 mg, at a total daily dose of 1500 mg, with respect to Cmax and AUC in healthy fed and fasted subjects. Following a single oral administration in the fed state of one tablet of metformin ER 1000 mg, a mean peak plasma concentration of 1214 ng/ml is achieved after a median time of 5 hours (range of 4 to 10 hours). Metformin ER 1000 mg was shown to be bioequivalent to metformin ER 500 mg, at a dose of 1000 mg, with respect to Cmax and AUC in healthy fed and fasted subjects. At steady state, both Cmax and AUC of metformin do not increase proportionally to the administered dose. Although the AUC is decreased by 30 % when the metformin prolonged release tablet is given under fasting conditions, the peak is neither modified nor delayed by fasting conditions. When the 1000 mg metformin prolonged release tablet is administered in fed conditions the AUC is increased by 77 % (Cmax is increased by 26 % and Tmax is slightly prolonged by about 1

hour) relative to intake in the fasting state. Although there is no information on the exposure after the 750 mg and 500 mg prolonged release tablets. It is presumed that similar increased exposure occurs with these formulations when given in the fed-state.

Distribution: Plasma protein binding is negligible. Metformin partitions into erythrocytes. The blood peak concentration is less than the plasma peak and appears approximately at the same time. The mean volume of distribution (Vd) ranged between 63 – 276.

Metabolism: Metformin is eliminated unchanged in the urine. No metabolite has been identified in humans. There is no biliary excretion.

Elimination: Metformin renal clearance (>400 ml/min) shows elimination by glomerular filtration and by tubular secretion. After oral intake, the apparent terminal elimination half-life is approximately 6,5 hours.

Pharmacokinetics in preterm preeclampsia

As part of the PI 2 trial, an open label pharmacokinetics study of 15 women with preterm preeclampsia was performed.[31]1.5 grams of oral metformin ER was given twice a day. Plasma was taken via an intravenous cannula on the first day of treatment at 2, 4, 5, 6, 7 and 8-hours post dosing. A 24-hour morning trough sample, 12 hours after the night-time dose was also collected. To calculate area under the concentration time curve (AUC) 0-12 it was inferred that the morning trough concentration would be similar to the evening trough concentration. Morning trough concentrations were also measured on day 5. The maximum (peak) plasma concentrations (Cmax), minimum concentrations (Cmin) and AUC0-12 were determined. The AUC0-24 was estimated by doubling the AUC0-12 as participants received metformin twice a day.

This pharmacokinetic study showed that a daily dose of 3 grams of metformin ER administered to women with preterm preeclampsia exhibited similar pharmacokinetics to non-pregnant persons taking a daily dose of 2 grams. At the 3 gram dose, there was good 24 hour drug levels in the circulation among women with preterm preeclampsia.

Summary

Preterm preeclampsia is a devastating disease. At extremely preterm gestations this subjects the neonate to the risk of significant morbidity and mortality.

Preclinical and clinical data have uncovered potent biological actions suggesting metformin is a therapeutic candidate for preeclampsia. Treatment with metformin in pregnancy is considered safe as it is already used extensively to treat diabetes during pregnancy.

The PI2 trial showed that administering metformin ER to pregnant women diagnosed with preterm preeclampsia might prolong gestation and decreased neonatal length of stay in a South African population of mostly African women. In this trial we aim to confirm these findings in a different population group.

4. Trial objectives

4.1. Primary objective

To examine whether 3 grams of metformin ER daily can prolong gestation in pregnancies complicated by preterm preeclampsia diagnosed 22+0–33+6 weeks in women undergoing expectant management, compared to placebo.

4.2. Secondary objectives

To determine whether 3 grams of metformin ER daily can reduce the time from birth to discharge from neonatal care in neonates born to women with preterm preeclampsia diagnosed at 22+0–33+6 weeks undergoing expectant management, compared to placebo.

To determine whether 3 grams of metformin ER daily can increase neonatal birthweight of neonates born to women with preterm preeclampsia diagnosed at 22+0–33+6 weeks undergoing expectant management, compared to placebo.

4.3. Exploratory objectives

Exploratory objectives are to examine whether 3 grams of metformin ER compared with placebo daily can:

- Reduce maternal and neonatal adverse outcomes including
    - Maternal death
    - Eclampsia
    - Cerebral vascular event
    - Posterior reversible encephalopathy syndrome
    - Pulmonary oedema
    - Left ventricular failure
    - Renal dialysis
    - Renal failure
    - HELLP (Haemolysis, elevated liver enzymes and low platelets) syndrome
    - Thrombocytopenia (Platelets <100 X 10^9/L)
    - Liver transaminases >500 IU/L
    - Liver haematoma or rupture
    - Admission to an intensive care unit
    - Severe hypertension
    - Disseminated intravascular coagulation

- Caesarean section
- Major postpartum hemorrhage
- Thromboembolic disease
- Mother's length of stay in hospital postpartum
- Number of antihypertensive drugs at discharge after delivery
- Duration of antihypertensive medication
- Intrauterine foetal demise
- Neonatal death
- Growth restriction at birth (defined as less than 2 standard deviations from median weight from Swedish birth weight growth curves).
- Grade 3 or 4 intraventricular haemorrhage
- Necrotizing enterocolitis
- Reversed a-wave in the ductus venosus
- Persistent reversed flow in the umbilical artery
- Redistribution in the middle cerebral artery
- Low APGAR score, defined as less than 4 at 5 minutes
- Umbilical artery pH below 7.1 ´
- Invasive ventilation, intubation and mechanical ventilation or non-invasive ventilation; continuous positive airway pressure (CPAP) support or high flow nasal oxygen
- Grade III/IV hyaline membrane disease
- Surfactant use
- Retinopathy of prematurity
- Neonatal sepsis

* Be proven tolerable, safe and that women remain compliant by measurement of
  - Nausea
  - Vomiting
  - Diarrhoea
  - Headache
  - Lactic acidosis
  - Drug adherence
* Reduce circulating concentrations of the preeclampsia related biomarkers

- Soluble Fms-like tyrosine kinase-1
- Placental growth factor
- Soluble endoglin

- Affect the gut and vaginal microbiome composition and produce biologically active metabolites
- Improve or at least not decrease patient-reported experience measures including
  - Breastfeeding initiation
  - Depressive symptoms
  - Self-esteem
  - Perceived stress
  - Breastfeeding self-efficacy
  - Health-related quality of life
  - Parental-infant bonding
- Be cost-effective
- Affect the cognitive development of the child

Factors to support inclusion in a clinical trial will also be evaluated.

4.4. Primary endpoint

The primary endpoint is prolongation of gestation, defined as hours from randomisation to delivery.

4.5. Secondary endpoints

- Length of stay in neonatal care, defined as hours from birth to 12 AM at day of discharge from neonatal care, including both hospital and home-based care.
- Neonatal birthweight, in grams.

4.6. Exploratory endpoints

These are described in section 8.

5. Trial design and procedures

5.1. Overall trial design

Phase III, double-blinded, placebo-controlled, parallel-group multi-centre trial of metformin ER compared to placebo to prolong gestation in preterm preeclampsia. The study design is chosen in order to achieve highest possible level of evidence for

the potential effect of the trial drug. The main outcome prolongation of pregnancy is chosen due to its large clinical impact regarding reduction of preterm birth in preeclampsia. The multi-centre approach is chosen due to the rarity of the disorder and generalisability of results. Fourteen Nordic main sites (predominately University Hospitals) participate in the trial. Norway will have a National PI according to local regulations, which is not required in Sweden and Finland. To facilitate management of participants that are moved to neighbouring hospitals after inclusion and to increase inclusion rates, a decentralised approach will also be applied. Four smaller hospitals in Sweden within the catchment area of their respective main centre will be included as satellite sites in the trial (see Attachment G). Site agreements are established between sponsor and every site.

Each study participant will be treated with the trial drug from randomisation to delivery (the same day as caesarean section or when in active labour). The primary and secondary aims are included in the original trial. All other outcomes are analyzed separately and are not dependent on the blinding/unblinding of treatment arms. The study is divided in Part A (from start to two-three months follow up and Code breaking) and Part B (Follow up beyond primary and secondary outcomes including two year follow up of participant and child in Swedish and Norwegian registries and Swedish, Norwegian and Finnish medical records). Type of biological samples taken differ between countries (see Table 1).

Figure 1. Trial diagram

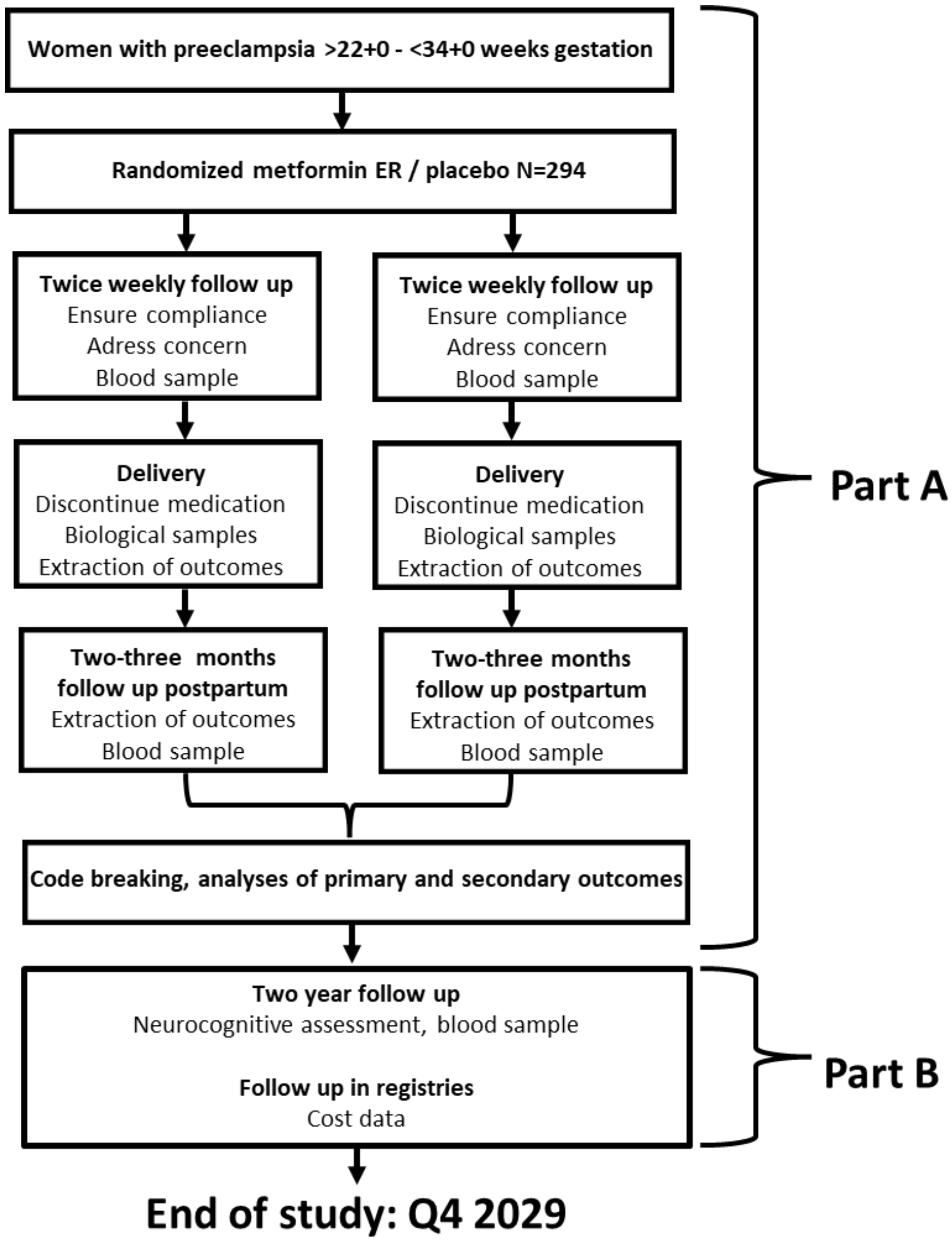

5.2. Procedures and flow chart

Inclusion

Potential participants with a diagnosis of preterm preeclampsia (defined as hypertension in combination with significant proteinuria (albumin/creatinine ratio ≥8 mg/mmol, protein/creatinine ratio>30 mg/mmol or >2+ protein on a urinary dipstick) with or without other organ manifestations from gestational week 22+0 to gestational week 33+6) will be identified at one of the participating main centres or at satellite centres within the catchment area during an in- or outpatient visit. Organ manifestations are defined according to the definition of preeclampsia in guidelines from the participating Nordic countries (Swedish: https://www.sfog.se/kunskap/preklampsi/, Finnish: https://www.kaypahoito.fi/sv/gvr00094, Norwegian: https://metodebok.no/emne/K4U6nNQ3/hypertensive-svangerskapskomplikasjoner-og-eklampsi-(2020)/f%C3%B8dselshjelp-(ngf)). In this trial, significant proteinuria as per above will be required for all eligible women to limit the

heterogeneity of the population. The clinical treatment team will decide if she qualifies for expectant management and is suitable for admission as per the hospital protocol (potential for recruitment), or if delivery is likely within 48 hours (exclusion criteria). The research nurse or site principal investigator will be notified by the ward staff and/or clinical treatment team.

The potential participant will receive information about the trial. A qualified trial physician, with GCP training will assess the eligibility criteria and get informed consent, after answering all questions the potential participant has. After she has signed informed consent, she will be randomised to active treatment or placebo (see section 7.5). A study nurse / nurse assistant will document demographics and clinical signs and symptoms in the eCRF and in addition, the woman will complete four questionnaires according to section 5.4. Treatment packs will be allocated to the patient according to the randomisation number. The participant will receive instructions on how to take the drug to minimize side effects and will be informed about the treatment diary. She will also receive information about how to report side effects to the research nurse if these occur in between the visits. The diary will be accessed through the mobile application MyCap which is connected to the eCRF in RedCap (see Attachment A). There will also be an option for the participant to use a printed version to bring for the follow up visits that are then entered into the eCRF by the research nurse.

Standard clinical care will be maintained throughout the study according to local guidelines and decisions by the managing clinician. A separate informed consent form will be signed by the legal guardian or guardians of the child regarding follow up data of the child and for potential analyses of the umbilical cord sample taken at delivery. A qualified trial physician, with GCP training will get informed consent post partum, after answering all questions the legal guardian or gardians have

Follow up visits during pregnancy

Following a diagnosis of preeclampsia, women are either cared for inpatient or outpatient. They are closely monitored regarding blood pressure, clinical signs and symptoms and biochemical assessment of liver-, coagulation- and renal function. Participants in the trial will, in addition to standard care, also be followed twice weekly with questionnaires including drug compliance and side effects. Tolerability, safety and adherence will be recorded by the study nurse / nurse assistant twice a week between randomisation and delivery through a treatment diary that the woman will fill in daily (Attachment A). At Swedish sites, safety and tolerability will be measured by assessing tolerability according to the PHASE-20 questionnaire (Attachment B). Adherence will be measured by patient-completed treatment diaries and pill counts.

Normal clinical care will be maintained throughout the study. According to Nordic guidelines, this includes analyses of biochemical markers for preeclampsia at a minimum of two times per week and blood pressure monitoring at a minimum of two times per week if outpatient and daily if inpatient. The use of steroids, antihypertensive agents and magnesium sulphate will be administered according to local protocols and will be decided upon by the managing clinician. The use of aspirin and calcium will be noted. In rare occasions, an included participant might be transferred to another hospital during the trial depending on availability of neonatal beds Primarily, the woman will be transferred within her region. If transferred, the receiving hospital will obtain written information about the clinical trial. This will include information about the trial drug, contact information to the research nurse at the recruiting center and a copy of the informed consent form.

If an included participant is transferred to another hospital, the recruiting site will remain responsible for the participant during the trial. The bi-weekly follow up will be conducted by the research nurse at the recruiting site by teleconference or physically, depending on the distance to the receiving hospital. The participant will also be able to contact the research nurse at the recruiting site between the scheduled follow up calls / visits. Data from the medical chart or through interview with the participant will be retrieved by the research nurse at the recruiting center. In these cases, necessary approval documents from the local hospital to retrieve information from the medical chart will be obtained.

At delivery

At delivery, the treatment with the trial drug will be stopped. The woman and her neonate will be monitored and treated according to local protocols. Diagnoses and outcomes will be registered from the medical charts. Biological samples will be obtained (see section 5.3).
If the participant gives birth at another hospital than where she was included, remaining drug will be sent back to the center where she was included for pill count and return to the pharmacy for destruction.

Two-three months follow up visit postpartum

The woman will be followed up at two-three months postpartum in accordance with recommendations of follow up after a pregnancy complicated by preeclampsia. The follow up visit could be separate or in combination with the routine clinical follow up visit. Additional biological samples will be obtained from the mother. She will also fill in a questionnaire regarding signs and symptoms and questionnaires according to section 5.4. A blood pressure,urine sample will be obtained as part of her routine clinical follow up.

Two year follow up visit postpartum (Part B)

Participants will be invited to the two year follow up visit between 23+0 and 25.5 months post birth corrected age. However, the follow up visits might occur after this timeframe if needed. The participant will fill in a questionnaire for patient-reported measures according to section 5.4. Biological samples will also be obtained, as well as blood pressure. The participant will also be asked questions about her current health and any prescribed medication. The legal guardians will be asked to report their child's neurodevelopmental status using the Ages and Stages Questionnaire, Third Edition (ASQ) 24-month questionnaire. The ASQ contains five subdomains: communication, gross motor, fine motor, problem solving, and personal-social, each consisting of six items with a maximum score of 60, resulting in a maximum total score of 300 (higher scores indicate more developmental milestones reached). To assess the risk of autism spectrum disorders, the Modified Checklist for Autism in Toddlers, Revised (M-CHAT-R) will be used. In addition, for the Swedish and Norwegian population, data two years post partum from the Swedish Neonatal Quality Register (SNQ), the Norwegian Neonatal Quality Register and the Norwegian Patient Register on Bayley Scales of Infant Development-4 and other variables related to social, behavioral, cognitive and motor development will be analyzed for children born before gestational week 28 or with a birthweight of ≤ 3 SD. Retrieved data will be validated in medical records. Weight (kg), height (cm) and body mass index (kg/m^2) will also be measured. Follow up data for children up to two years post partum will also be retrieved from Swedish, Norwegian and Finnish medical records.

Table 1. Flowchart of the trial

PI 4	Screening/ Randomisation	After 5 days of treatment ± 1 day	Recurrent visits two times weekly	Delivery	Discharge visit	Two-three months follow up	Two years follow up
**In/exclusion criteria**	A B C						
**Informed consent**	A[1] B[1] C[1]			A[2] B[2] C[2]			
**Patient information and characteristics**	A B C					A B C	A B C
**Questionnaires for mother**	A B C					A B C	A B C
**Questionnaires for child**							A B C
**Registry data/Medical records for child**							A B C
**Drug instruction**	A B C						
**Biological samples for the mother**	A[3,4,5] B[3,4] C[3,4 5]	A[5] C[5]	A[3] B[3*] C[3*]	A[3,6] B[3,] C[3]		A[3, 4] C[3, 4]	A[3,4,5] C[3,4 5]
**Placental samples**				A C			
**Cord blood sample**				A B C			
**Weight, height and body mass index of the child**							A B C
**Blood analyses in clinical routine**	A B C		A B C	A B C	A B C	A B C	
**Tolerability, safety and adherence/drug compliance**			A B C	A B C			
**Incidents (AE & SAE)**			A B C	A B C	A B C	A B C	
**Discontinue trial medication**				A B C			
**Primary and secondary outcomes**				A B C	A B C	A B C	
**Exploratory outcomes**				A B C	A B C	A B C	A B C
**Cost data**							A B C

A Procedures in Swedish sites
B Procedures in Finnish sites
C Procedures in Norwegian sites
[1]Informed consent for the mother
[2]Informed consent regarding the child
[3]Blood sample
[4]Urine sample
[5]Rectal and vaginal swabs
[6]Cerebrospinal fluid sample
*At one occasion

5.3. Biological sampling procedures

5.3.1. Handling, storage, and destruction of biological samples

All biological samples will be collected by trained research staff or clinical staff. All samples in the trial are handled according to current biobank rules and regulations in Sweden and Norway. The law controls the way in which biological samples can be stored and used and in addition the law controls the requirements of quality and security for biobanks. The biological samples collected in Finland are exempt from the Finnish Biobank Act (688/2012) as they are collected exclusively for analysis within the scope of this specific trial, and will be immediately destroyed following analysis. Consequently, the samples will not be stored for future, unspecified biobank research.

Blood samples will preferably be obtained simultaneously as blood samples in routine clinical care. Blood samples will be obtained at inclusion in the study (maximum 50 ml) and two times weekly until delivery (maximum 20 ml per visit). Blood samples will also be collected at delivery (maximum 20 ml),at the two–three months and two-year follow up (Part B) visits (maximum 40 ml per visit).

If the participant is transferred to another hospital that is not participating in the trial during her pregnancy due to shortage of neonatal beds, the local staff will be asked to draw blood samples twice weekly and to obtain biological samples including blood samples at delivery. All material for these samples will be provided by the recruiting center. This is described in detail in “Följsamhet med regler för hantering av biologiska prov”. In rare cases where biosamples will not be possible to obtain, these will be considered as missing. Results from biosamples are not included as main outcomes in the study and are not affecting the safety of the trial.

At delivery, placental samples and cord blood (maximum 10 ml) will be collected. In case of administration of spinal anaesthesia (such as in the second stage of labour, at caesarean section or at surgery postpartum), a cerebrospinal fluid sample (maximum 10 ml) will be collected.

Urine samples will be collected at inclusion and subsequently at the two-three months, and two year follow up visit (Part B) (maximum 10 ml).

Self administred rectal and vaginal swabs will be collected at inclusion (before treatment initiation),after 5 days of treatment +- 1 day, and at the two year follow up visit (Part B).

Analyses will be conducted for angiogenic biomarkers at an accredited laboratorium on validated instruments. Samples will also be stored for later analyses that will require a new ethical permit

5.3.2. Total volume of blood per subject

The total volume of blood taken from each subject during the study is entirely depending on the length of the period between inclusion and delivery, determininig the number of plasma samples taken twice a week during this period. For the majority of subjects, pregnancy will last maximum 40 days (5-6 weeks) after inclusion, resulting in a maximum total volume of

400 ml during the study. In rare cases (a few percent of the subjects), pregnancy can continue after 6 and up to 15 weeks from inclusion to delivery, resulting in a maximum total volume of 750 ml (distributed over several months).

5.3.3. Biobank

All biobank samples taken at Swedish sites are registered in sample collections in participating Swedish Regions' biobanks. The samples are coded/pseudonymized to protect the subject's identification. The code list is held by the local biobank and/or the local PI. The code list and sample associated data can be made available for the sponsor's representative (coordinating PI) and the recipient biobank. All samples and the code list are stored securely and separately to prevent unauthorized persons from having access to them.

Biological samples taken in Finnish sites will be handled and stored in Hospital laboratories and shipped to the sponsor in Sweden for analyses at the end of the trial according to Material Transfer Agreements (MTAs). Biological samples taken in Norwegian sites will be handled and stored in a project specific biobank at Oslo University Hospital Rikshospitalet (PI [redacted]) and shipped to the sponsor in Sweden at the end of the trial according to Material Transfer Agreements (MTAs). The samples will be stored in a freezer at the University of Gothenburg run by the sponsor. After finalization of the trial, samples will be analysed for biomarkers related to preeclampsia. After all analyses are performed, samples will be destroyed.

Plasma and serum samples will be analysed for preeclampsia related biomarkers at appropriate laboratories and MTAs will be established as needed.

5.4. Procedures for obtaining patient reported experience measures

Patient-reported measures will be obtained by questionnaires using validated instruments distributed at inclusion, at two-three months, and at the two year follow up (Part B) visit, as well as by qualitative interview studies. All questionnaires are included as Attachment D, and will be answered on tablets or by paper and pen. All questionnaires are available in Swedish and English. Finnish, Norwegian, Arabic and Somali questionnaires are offered when validated translations are available. At inclusion, the Edinburgh Postnatal Depression Scale (EPDS); self-esteem by the Rosenberg Self-Esteem Scale (RSE), health-related quality of life (EQ-5D),and stress by the Perceived Stress Scale (PSS) will be assessed. At follow-up 2-3 months postpartum, health-related quality of life will be measured by EQ-5D, postpartum bonding will be evaluated by the Postpartum Bonding Questionnaire (PBQ), breastfeeding self-efficacy will be determined by the Breastfeeding self-efficacy scale short-form (BFS); postnatal depression will be measured by EPDS; self-esteem by the RSE and stress by PSS. Questions regarding feeding the baby, breast milk /formula, will also be included. To asses why women chose to participate in the drug trial, short semi-structured interviews with quantifiable answers will be conducted by the research nurse at 2-3 months postpartum. The participants will also be asked what group they think they may have been allocated to, and why they believe that. To measure possible side effects of the metformin, the participants will be asked to fill out a symptom evaluation questionnaire, the PHASE-20 – PHArmacotherapeutical Symptom Evaluation scale, at the bi-weekly assessments during ongoing drug treatment (Attachment B). At the two year follow-up (Part B), the following

questionnaires will be included: the Edinburgh Postnatal Depression Scale (EPDS); self-esteem by the Rosenberg Self-Esteem Scale (RSE), health-related quality of life (EQ-5D),and stress by the Perceived Stress Scale (PSS). Information regarding duration of breastfeeding will also be collected.

5.5. Follow up in Nordic registries up to two years post partum (Part B)

5.5.1. Procedures for obtaining cost data

Cost data will be retrieved for the Swedish and Norwegian populations from registers and medical records and for the Finnish population from medical records only. Economic evaluations are used to assess if a specific treatment is worth the investment, through saving future costs or through any resulting health gains being achieved at an acceptable cost. According to national guidelines, such analyses should employ a societal perspective, thus including all costs and resource use affected by the therapy, regardless of who is the payer. The economic evaluation will thus include drug use and healthcare costs for mother and child from enrolment and until two years post partum, social insurance transaction associated with caring for the child as well as productivity loss associated with sick leave and parental leave. All costs will be estimated from a societal perspective, including costs both to the health system and families. Discounting and sensitivity analyses will be conducted according to state of the art.

To provide a complete picture of costs and enable interpretation of results for other countries and health systems, costs will be reported and analysed both by specific components (e.g., type of resource use/encounters and caregiver categories) and by diagnoses (ICD-codes and ATC-codes, respectively), particular study diagnoses (for example but not restricted to maternal postpartum complications (O- , N- , S- and T chapter), cardiovascular and metabolic health for mother and child (I- and E chapter) and neonatal health (P chapter), will be reported in detail while costs associated with other diagnoses are reported on a more aggregated level.

Costs for drugs dispensed in community pharmacies will be obtained retrospectively from the Swedish Prescribed Drug Register (SPDR, held by the Swedish National Board of Health and Welfare), and the Norwegian Prescribed Drug Registry (NorPD, held by the Norwegian Institute of Public health), which include both patient out-of-pocket costs for prescription drugs outside the reimbursement system and costs for use of the mother and child within the reimbursement system if they have not yet reached the annual ceiling, and they also include costs reimbursed by the regions. While drugs indented to the healthcare units and administered during visits are not available in the registers, the more expensive drugs are expected to be reported and will thus be identify by research staff from medical records, the Swedish National Medical Birth Register (MFR, held by the National Board of Health and Welfare), the Medical Birth Registry of Norway,The Swedish Neonatal Quality Register (SNQ, helt by the Quality Register Center [QRC] Stockholm), and the Norwegian Neonatal Quality Register (held by the National Board of Health and Welfare) . Information about emigration or death will be retrieved from the cause of death register in both Sweden and Norway. The time frame will be limited to two years post partum.

The short-term cost calculation ensures that a partial cost-effectiveness analysis can be presented within the initial funding period. In addition to drug costs, it will be based on administrative cost data from the local hospital registers, including costs for mothers during the initial treatment and pregnancy period, during birth, and for mother and child until discharge. However, for the long-term follow-up, costs for healthcare, both for the mother during pregnancy and childbirth follow-up until 2 years after the birth, and for the child, will be calculated based on resource use obtained retrospectively from the National Patient registers (in Sweden held by the National Board of Health and Welfare, and in Norway held by the Norwegian Institute of Public Health) for specialized outpatient and inpatient care, respectively. The cost calculation will primarily use diagnosis-related groups (DRG) reported in the registers, which combined with retrospectively reported DRG weights and costs statistics per one DRG provides an approximation of costs on group level. Cost per patient data from the same register will be used as a sensitivity analysis, but does currently not have complete coverage. Moreover, out-of-pocket costs for the mothers will be estimated using information from the regions on patient fees up til the annual cost ceiling, According to state of the art. Child healthcare does not incur patient fees. Since primary care, including child health services, are not included in any regional register, use will be collected during the study by research staff.

Parents are expected to be on parental leave during some of this study period, thus productivity loss resulting from sick leave and parental leave will be calculated based on questionnaire data during the study, as well as any assistance services and other resource use resulting from caring for the child. Social insurance transaction costs will be calculated for the Swedish population based on payments paid to parents or other responsible adults for goods and services used in caring for the included child, which will be obtained from the Micro Data for the Analysis of Social Insurance register (MiDAS), held by the Social Insurance Agency and from the LISA register. See variable list in Attachment C. Information from the registers as described above will only be used for above assessment for the period from inclusion to two years after the child's birth.

5.6. Start, end, temporary halt and early termination

5.6.1 Start of the clinical trial

The start of the trial is defined as recruitment of the first participant, i.e. February 6 2024. Sponsor will report the start of the trial in each participating country through notification in the CTIS within 15 days.

5.6.2 Temporary halt or early termination

The drug trial (Part A) may be prematurely terminated if it appears that the treatment involved a large number of serious adverse events (SAE) or if recruitment of subjects cannot be met within reasonable time limits. Decisions on premature termination are taken by the sponsor. If the study is prematurely terminated or suspended, the investigator should immediately inform the subjects about this and ensure appropriate treatment and follow-up. The regulatory authority should be informed as soon as possible, but no later than within 15 days.

5.6.3 End of the clinical trial

The study ends after the two-year follow up visits of all participants are completed.

6. Subject selection

6.1. Inclusion criteria

To be included in the study, subjects must meet the following criteria:

- A diagnosis of preeclampsia has been made (as defined in 5.2) by the attending clinician
- The managing clinicians have made the assessment to proceed with expectant management
- The subject has given written consent to participate in the study.
- The woman must be 18 years of age or older
- The gestational age is between 22+0 weeks to 33+6 weeks with a viable fetus, defined as the following:
    - There is a live fetus when assessed with ultrasound where the prognosis is considered such that the fetus can survive a birth with interventions from neonatal care
    - If the pregnancy is over 23 weeks of gestation, it has been decided to deliver by caesarean section if there is an indication for delivery (except for spontaneous start of labour)
    - There must be a decision for active fetal monitoring, up to 24+0 weeks with ausculation of fetal heartbeat and over 24+0 weeks with CTG.
    - All eligible women in PI4 with pregnancies 22+0-24+0, or in case of uncertainty about viability regardless of gestational week, must be discussed with the sponsor ([redacted]) before inclusion.
- The woman carries a singleton pregnancy

6.2. Exclusion criteria

Subjects must not be included in the study if any of the following criteria are met:

- There are contraindications to treatment with metformin as outlined in SmPC (Attachment E)
- Contraindications for expectant management of preeclampsia such as an immediate indication for delivery according to Nordic guidelines.
- Type 1 Diabetes Mellitus
- Current use of metformin
- Known or suspected allergies against metformin
- Reluctance or language difficulties that result in difficulty understanding the meaning of study participation
- Unable to understand the informed consent process
- Previous participation in the study

- Established fetal compromise that necessitates imminent delivery (including planned delivery after 48 hours of corticosteroid treatment). This will be decided by the clinical team before expectant management is offered to the patient.
- Suspicion of a major known fetal anomaly or malformation.
- Renal disease or dysfunction, suggested by a creatinine level greater than or equal to 125 µmol/L or rapidly declining renal function
- Known acute or chronic metabolic acidosis, including diabetic ketoacidosis
- Not suitable for inclusion by the opinion of the investigator

6.3. Screening and inclusion

Subject eligibility (that subjects fulfil all inclusion criteria and do not meet any exclusion criteria) is established before inclusion, treatment, or randomisation.

Potential participants will be identified when they are seeking health care at one of the participating centres with a diagnosis of preterm preeclampsia (<34+0 gestational weeks). The clinical management team will decide if the patient qualifies for expectant management and is suitable for admission as per the hospital protocol (potential for recruitment), or delivery is likely within 48 hours (exclusion criteria). The research midwife and principal investigator will be notified by the ward staff and/or clinical treatment team. If a previous exclusion criteria is no longer present, the woman can be re-screened.

6.4. Withdrawal criteria

Subjects can discontinue their participation in the study at any time without any explanation. There will be no consequence to her continued treatment. The investigator/sponsor can at any time terminate the study for a subject due to, e.g., unacceptable adverse events/adverse reactions or because the subject does not follow procedures in the study protocol. If the subject discontinues the study, follow-up of this subject will be performed according to the clinic's routine. Basic clinical data and samples already collected will be included in the analysis in accordance with the consent obtained at trial entry. If the trial participant wants to withdraw biological samples, these will be destroyed.

Specific stopping criteria:

- Lactacidosis
- Unacceptable adverse events/adverse reactions
- The subject does not follow procedures in the study protocol
- Renal disease or renal dysfunction (suggested by serum creatinine levels ≥125 umol/L or abnormal creatinine clearance) which may also result from conditions such as cardiovascular collapse (shock), acute myocardial infarction, septicaemia and preeclampsia causing renal failure.
- Acute or chronic metabolic acidosis
- Acute conditions with the potential to alter renal function, e.g., dehydration, severe infection, shock, intravascular administration of iodinated contrast media.
- Acute or chronic disease which may cause tissue hypoxia such as cardiac or respiratory failure, pancreatitis, recent myocardial infarction, shock.
- Hepatic insufficiency, acute alcohol intoxication, alcoholism (acute or chronic).

Stopping criteria where metformin can be resumed if/when the condition resolves

- Gastroenteritis
- Surgery under general anasthesia
- High fever
- Need of contrast radiography

7. Trial treatments

7.1. Description of the investigational medicinal product

The trial medication (metformin ER tablets and identical placebo tablets) will be produced by Merck at sites within the EU and sent in bulk to Apotek Produktion och Laboratorier (APL). A description of the active drug and placebo is outlined in Attachment E.

7.2. Storage of trial medications

The packaged medication will be stored at Tamro AB and distributed to the trial sites. Once a participant is randomised, a treatment pack will be issued.

7.3. Dose and administration

Each participant will be given a treatment pack by the study nurse. A copy of the randomisation (from the randomisation website) will be placed in the participant's trial folder. The treatment pack will be labelled with the participants randomisation number. A patient kit will consist of two labelled bottles in one labelled box. A patient kit contains 8 weeks (56 days) of treatment (336 tablets). Treatment will then be taken as described previously. Trial drugs will under no circumstances be used for other participants. The treatment will be initiated by one 500 mg tablet three times daily and increased to one gram three times daily as tolerated. In the event of side effects, the dose will be decreased to one 500 mg three times daily or until tolerated. The dose may be increased again as tolerated, often by one tablet each day. The participant is informed at inclusion and in the treatment diary that treatment should be stopped in case of gastroenteritis or dehydration and that the participant should then contact the research nurse for further instructions. All doses are documented in the treatment diary either directly through the mobile application or on the paper diary (Attachment A). Dosage procedures and treatment responsibility for Finland are outlined in Attachment H.

For most participants, delivery will occur within 56 days of randomisation. However, in some cases delivery may occur after 56 days of randomisation. When this happens continuation trial drug packs will be available: the principal investigator will contact APL and ask for a number for a continuation pack that will be matched to the contents of the initial treatment pack which will contain a further 28-day supply of the allocated treatment.

7.4. Packaging, labelling, and handling of investigational medicinal products(s)

After delivery of the trial medication from Merck in bulk, it will be packaged and labelled by APL. The trial medication will be placed into containers and labelled randomly according to

the computer-generated randomisation list. Labelling and preparation will be done according to the requirements of the Medicines for Human Use (Clinical Trials) regulations. The packages will subsequently be delivered to the Tamro AB in Gothenburg, Sweden for storage and distribution in blocks to sites.

7.5. Drug accountability and treatment compliance

The research nurse will provide the participant instructions on how to complete a treatment diary where the participant will fill in the number of tablets taken daily and side effects (Attachment A). The treatment diary will be filled in electronically by the participant through the mobile application or by the research nurse if the paper form of the treatment diary is used. If the participant experience side effects that can not be tolerated, the dose will be decreased until the treatment is tolerated. As a second compliance check the patient will be asked to return the empty packaging to the research nurse who will record the number of tablets left in the treatment pack at the time of delivery of the patient. If the participant forgets to take a dose, this is entered as 0 in the treatment diary. If the patient takes the tablet late, up to four hours after intended time of intake is accepted but noted in the treatment diary.

7.6. Randomisation

Subjects will be randomised consecutively as they are found to be eligible for inclusion in the trial. If a subject discontinues their participation, the subject's trial-specific code will not be reused, and the subject will not be allowed to re-enter the trial again.

Participants will be randomised 1:1 to either of the two treatments using random permutated blocks of varying sizes, stratified by treatment site and gestational age at inclusion (22+0–28+6 or 29+0–33+6 weeks).

An online, web-based system will be used for randomisation. Researchers and participants will be blinded to treatment allocation.

7.7. Blinding

All treatment packs will be identical and will contain either active tablets or placebo. The treatment packs and labelling will be prepared and issued by APL. Folded leaflet with information in Swedish, Finnish, Norwegian and Danish will be used for labelling on cans and boxes. Labelling for site will be issued at Tamro AB. The researchers, clinicians and participants will not have access to the randomisation list. This process will ensure that there is allocation concealment throughout the conduct of the trial. The allocation will only be released once the trial is completed and the database has been locked.

7.8. Code breaking

The need for code breaking should be very uncommon as the trial intervention is rarely associated with severe side effects and it will not delay or prevent standard management of the patient. If, however, code breaking is needed for any reason the principal investigator will be informed and, if necessary, the treatment will be revealed. Sealed envelopes with the treatment code for code breaking are created by the statistician, provided by Tamro AB together with the trial medication and stored at respective site with local PI.

7.9. Auxiliary medicinal products

There are no auxiliary medicinal products for this trial.

7.10. Concomitant use of other medicinal products and treatments

It is very unlikely that the investigators will need to supply concomitant medication. If medications are considered necessary for the safety and well-being of the subject the investigator may provide treatment at their discretion, unless otherwise specified in the e xclusion criteria. Concomitant medication should be recorded in the eCRF.

7.10.1. Recommendations and precautions related to Metformin

Concurrent use of alcohol and iodinated contrast agents is not recommended.

The study drug must be discontinued before or at the time of contrast agent intake and must not be reintroduced until at least 48 hours later, provided that renal function has been re-evaluated and found to be stable.

7.10.2. Combinations requiring precautions

Some medication may adversely affect renal function which may increase the risk of lactic acidosis, such as NSAIDs, including selective cyclooxygenase (COX) II inhibitors, ACE inhibitors, angiotensin II receptor antagonists and diuretics, especially loop diuretics. When starting or using such products in combination with metformin, careful monitoring of renal function is necessary.

Co-administration of metformin with:

- Inhibitors of OCT1 (e.g. verapamil) may decrease the effect of metformin.

- Inducers of OCT1 (such as rifampicin) may increase the gastrointestinal absorption and efficacy of metformin.

- Inhibitors of OCT2 (such as cimetidine, dolutegravir, ranolazine, trimethoprim, vandetanib, isavuconazole) may decrease the renal elimination of metformin and thus lead to an increase in the plasma concentration of metformin.

- Inhibitors of both OCT1 and OCT2 (such as crizotinib, olaparib) may alter the efficacy and renal elimination of metformin.

Caution is therefore recommended, especially in patients with impaired renal function, when these medicinal products are co-administered with metformin, as the plasma concentration of metformin may increase. If necessary, dose adjustment (halving the dose) of study drug may be considered as OCT inhibitors/inducers may alter the effect of metformin.

7.11. Destruction

After delivery, the trial medication will be collected by the investigators and logged on the medication log. All medication including unused trial medications from Swedish sites will be destroyed by Tamro AB, and from Norwegian and Finnish sites by Hospital pharmacies.

7.12. Treatment after trial end

No further treatment will be provided after delivery. The participant will receive routine clinical care for women who have had a pregnancy complicated by preeclampsia.

8. Methods for measurement of endpoints for clinical efficacy and safety

8.1. Primary endpoint

The primary endpoint is prolongation of gestation, defined as days and hours from randomisation to delivery. Prolongation of gestation in pregnancies with intrauterine fetal demise will be considered as interval censored between time from randomisation to last visit preceding delivery (where the baby was known to be alive) and time from randomisation to delivery.

8.2. Secondary endpoints

Length of neonatal care, defined as hours from birth to 12 AM at day of discharge from neonatal care, including both hospital and home-based care. Length of neonatal stay will be as zero if the infant was not admitted to neonatal care.

Neonatal birthweight, in grams.

8.3. Exploratory endpoints

Maternal and neonatal adverse outcomes, including but not limited to

- Maternal death (binary), defined as death during pregnancy or within 42 days postpartum.
- Eclampsia (binary), defined as tonic-clonic seizures in the absence of other aetiology.
- Cerebral vascular event (binary), defined as stroke or intracerebral hemorrhage on computed tomography or magnetic resonance imaging
- Posterior reversible encephalopathy syndrome (binary), diagnosed on presence of neurologival symptoms in combination with cerebral edema on imaging
- Pulmonary oedema (binary), defined as oxygen saturation ≤90%, with clinical signs and symptoms requiring treatment.
- Left ventricular failure (binary), diagnosed on echocardiography as a left ventricular ejection fraction less than 40%
- Renal failure (binary), defined as serum creatinine greater than or equal to 125 µmol/L.
- Renal dialysis (binary)
- HELLP (Haemolysis, elevated liver enzymes and low platelets) syndrome (binary), defined as a platelet count less than 100 × 109/L, aspartate aminotransferase greater than 70 µg/L, and haemolysis as demonstrated by lactate dehydrogenase greater than or equal to 10 µkat/Lor haemolysis on a peripheral blood smear or a decreased haptoglobin level.
- Thrombocytopenia (binary), defined as platelet count less than 100 x 10^9/L
- Liver transaminases > 70 µg/L (binary)

- Liver haematoma or rupture (binary), defined on imaging or intraoperatively
- Placental abruption (binary), defined as retroplacental clot covering more than 15% or maternal surface or at diagnosis on histology
- Admission to intensive care unit (binary)
- Severe hypertension (binary), defined as a systolic blood pressure greater than 160 mm Hg and/or a diastolic blood pressure greater than 110 mm Hg despite anti-hypertensive treatment after randomisation and before delivery
- Disseminated intravascular coagulation (binary), defined as prolongation of prothrombin time and the activated partial thromboplastin time together with rapidly declining platelets
- Caesarean section (binary)
- Major postpartum haemorrhage (binary), defined as blood loss more than 1000 ml in the first 24 hours postpartum
- Thromboembolic disease (binary), defined as a deep.vein thrombosis, pulmonary embolus or both
- Mother's length of stay in hospital postpartum, defined as hours from delivery to discharge
- Antihypertensive drugs at discharge after delivery (nominal), defined as number of different drugs
- Antihypertensive medication (continuous), defined as days on antihypertensive medication after delivery to 2-3 months follow up visit
- Intrauterine foetal demise (binary), defined as death in utero from 22+0 gestational weeks).
- Neonatal death (binary), defined as death from birth until 28 days of age
- Fetal growth restriction at birth (binary), defined by the Swedish Marsal curves [32]
- Grade 3 or 4 intraventricular haemorrhage (binary), diagnosed on ultrasound.
- Necrotizing enterocolitis (binary), defined as necrotizing enterocolitis requiring surgery.
- Reversed a-wave in the ductus venosis (binary), diagnosed on fetal ultrasound
- Persistent reversed flow in the umbilical artery (binary), confirmed on two fetal ultrasounds
- Redistribution in the middle cerebral artery (binary)
- Low APGAR score (binary), defined as less than 4 at 5 minutes
- Umbilical artery pH below 7.1 (binary)
- Invasive ventilation, intubation and mechanical ventilation or non-invasive ventilation; continuous positive airway pressure (CPAP) support or high flow nasal oxygen (binary)
- Grade III/IV hyaline membrane disease (binary)
- Surfactant use (binary)
- Retinopathy of prematurity (binary), defined as requiring laser therapy or becacizumab treatment
- Neonatal sepsis (binary), defined as a confirmed laboratory bloodstream infection treated with a course of antibiotics

Tolerability, safety, and compliance by measurement of

- Nausea (binary, patient-reported)
- Vomiting (binary, patient-reported)
- Diarrhoea (binary, patient-reported), defined as loose, watery stools> 3 times/24 hours.
- Headache (binary, patient-reported)

- Lactic acidosis (binary), defined as blood pH< 7.35 or blood lactate concentration> 5 mM.
- Drug adherence (categorical; took all trial medication, decreased medication but continued taking, those who stopped all medication)

Circulating concentrations of the preeclampsia related biomarkers (continuous)

- Soluble Fms-like tyrosine kinase-1 (continuous)
- Placental growth factor (continuous)
- Soluble endoglin (continuous)
- Cerebral biomarkers (continuous)
- Endothelial biomarkers (continuous)
- Cardiac biomarkers (continuous)
- Renal biomarkers (continuous)
- Coagulation biomarkers (continuous)

Effects of metformin on the gut and vaginal microbiome composition and its potential to produce biologically active metabolites.

Patient-reported experience measures, including

- Breastfeeding self-efficacy (continuous), defined as total score of Breastfeeding self-efficacy questionnaire at 2-3 months follow-up.
- Edinburgh Postnatal Depression scale (numeric) at 2-3 months follow-up.
- Rosenberg Self-Esteem Scale (numeric) at 2-3 months follow-up.
- Perceived Stress Scale (numeric) at 2-3 months follow-up.
- EQ-5D (numeric) at inclusion and 2-3 months follow-up
- Postpartum Bonding Questionnaire (numeric) at 2-3 months follow-up

Cost-effectiveness including

- Short-term cost-effectiveness analysis from a hospital perspective; incremental cost-effectiveness ratio in which incremental hospital costs for mothers from enrolment to discharge after birth and for neonatal care, will be compared to incremental effects measured as prolongation of gestation.

Factors to support inclusion in a clinical drug trial, assessed by
- Short semi-structured interviews with quantifiable answers conducted by the research nurse at the 2-3 months follow-up,

9. Handling of Adverse Events

9.1. Definitions

9.1.1. Adverse Event (AE)

Adverse Event (AE): Any untoward medical occurrence in a clinical trial subject administered a medicinal product and, which does not necessarily have a causal relationship with the treatment, can be an unfavourable and unintended sign (including an abnormal laboratory discovery), symptom or disease temporally associated with the use of the

investigational medicinal product, whether or not related to the investigational medicinal product. All adverse events will be documented on data capture sheets

An adverse event includes

- Any unintentional, unfavourable clinical signs or symptoms deviating from the expected natural course of disease.
- Any new illness or disease
- Metformin can induce increased liver enzymes in 1% of cases. Since preeclampsia can also induce increased liver enzymes and the possible interaction between the two is not known , increased liver enzymes >2 times reference value will be included as an AE.

The following are not considered adverse events:

- A pre-existing condition (unless it worsens significantly in pregnancy over and above what may be expected with the concurrent diagnosis of preeclampsia).
- Diagnostic or therapeutic procedures related to preeclampsia such as surgery or imaging.
- Complications to preeclampsia are expected outcomes and included in the exploratory outcomes but will not be reported as adverse events. These include eclampsia, HELLP syndrome, renal failure and pulmonary edema and are listed in 8.3.
- Hospitalization due to preeclampsia or childbirth
- The most commonly reported side effects of Metformin that are nausea, vomiting, diarrhea (watery stools > 3 times/24 hours), abdominal pain, loss of appetite, headache, Vitamin B12 decrease/deficiency, taste disturbance, and lactacidosis (blood pH < 7.35 or blood lactate > 5 mM, see below under AESI).

9.1.2. Serious Adverse Event (SAE)

Serious adverse event (SAE): Any untoward medical occurrence that at any dose:

- leads to death
- is life-threatening
- requires inpatient hospitalization or prolongation of existing hospitalization not related to preeclampsia and complications of preeclampsia
- results in persistent or significant disability or incapacity
- results in a congenital anomaly/malformation
- Since affected liver function is a known but unusual adverse event for Metformin treatment and since the liver can be affected in women with preeclampsia, jaundice with bilirubin > 50 ul/L will also be reported as SAE.[33-35]

Medical and scientific assessment will be made to determine if an event is “serious” and whether it would prompt reporting in other situations, for example important medical events that may not be directly life-threatening or result in death or hospitalization but may compromise the study subject or may require intervention to prevent one of the other results set forth in the definitions above. These should also normally be considered as SAEs. For

this trial, lactic acidosis and renal failure requiring dialysis are defined as serious adverse events in addition to above definition.

Based on knowledge of the disease in question and expected clinical course, some events that are otherwise serious are not considered as SAEs in this trial. The following is a list of SAEs that shall not be reported as SAEs:

- Preterm delivery with the associated complications
- Adverse events related to the diagnosis of preeclampsia which include severe hypertension, intracranial haemorrhage, renal failure, abnormal hepatic function, disseminated intravascular coagulopathy, eclampsia, left ventricular failure, pulmonary oedema and haemorrhage.
- Complications of preterm birth that may be expected include sepsis, necrotising enterocolitis, respiratory complications, seizures, hypoglycaemia and intra-ventricular haemorrhage.

9.1.3. Suspected Unexpected Serious Adverse Reaction (SUSAR)

SUSAR: A reaction/event that is unexpected, serious, and suspected to be caused by the treatment, i.e., adverse reactions that are not included in the Investigator's Brochure (IB) or SmPC.

9.1.5.Adverse Evenst of Special Interest (AESI)

AESI: a known serious side effect of a particular substance, in this case the drug Metformin. Lactacidosis is an AESI since it is a known undesirable side effect according to SmPC and since the frequence of lactacidosis might be different in a population of women with preeclampsia.

9.2. Assessment of Adverse Events (AE)

9.2.1. Assessment of causal relationship

The investigator is responsible for determining whether there is a causal relationship between the AE/SAE and use of the investigational medicinal product.

Consideration should be given to whether there is a reasonable possibility of establishing a causal relationship between the adverse event and the investigational medicinal product based on the analysis of the available evidence.

All AE can be categorized as either likely related, possibly related, unlikely related or not related, in accordance with the definitions below:

Likely related: Clinical event, including abnormal results from laboratory analyses, occurring within a reasonable time after administration of the intervention/investigational medicinal product. It is unlikely that the event can be attributed to underlying disease or other medications but is most likely caused by the investigational medicinal product and its emergence is reasonable in relationship with use of the investigational medicinal product.

Possibly related: Clinical event, including abnormal results from laboratory analyses, occurring within a reasonable time after administration of the intervention/investigational medicinal product. The event could be explained by the investigational medicinal product and its emergence is reasonable in relationship with use of the investigational medicinal product, but there is insufficient information to determine the relationship. The event could be explained by an underlying disease or other medications.

Unlikely related: Clinical event, including abnormal responses from laboratory tests, unlikely to be related to the intervention/investigational medicinal product and can be reasonably explained by other medication or underlying disease.

Not related: Clinical event, including abnormal results from laboratory analyses, that is not reasonably related to the use of the intervention/investigational medicinal product.

If the reporting investigator does not provide any information on causality, the sponsor should consult with the reporting investigator and encourage the expression of a position on this issue. The sponsor must take into account the assessment of causality provided by the investigator. If the sponsor disagrees with the investigator's assessment of causality, both the investigator's and the sponsor's views should be included in the report.

9.2.2. Assessment of intensity

Each adverse event shall be classified by an investigator as mild, moderate or severe.

Mild: The adverse event is relatively tolerable and transient in nature but does not affect the subject’s normal life.

Moderate: The adverse event causes deterioration of function but does not affect health. The event can be sufficiently unpleasant and interferes with normal activities but does not completely obstruct them.

Severe: The adverse event causes deterioration of function or work ability or poses a health risk to the subject.

9.2.3. Assessment of seriousness

The investigator is responsible for assessing the seriousness (serious or non-serious). If the adverse event is considered serious, this should be reported as a serious adverse event (SAE) by the investigator to the sponsor.

9.3. Reporting and registration of Adverse Events

At each trial visit, adverse events (AE) are registered, starting after start of treatment with the trial drug, up to and including two-three months after the subject has ended their treatment with the investigational medicinal product. If the participant is transferred to another hospital, AE will be registered by the research nurse from the recruiting sites at follow up visits or in between visits if the participant contacts the research nurse. All AE that occurs during the trial and which are observed by the investigator/trial nurse or reported by the subject will be registered in the eCRF regardless of whether they are related to the investigational medicinal product or not. Assessment of causal relationship, severity, and whether the AE is considered to be an SAE will be made by the investigator and entered in the eCRF. At

minimum for each AE/SAE, a description of the event is recorded (diagnosis/symptom if diagnosis is missing), start and stop dates, causal relationship, severity, if the AE is considered to be an SAE, measures and outcome.

The following symptoms are clearly related to the process and the expected course of the condition and therefore will not be reported as AE:
Expected adverse events based on knowledge of the disease in question and expected clinical course.

9.3.1. Reporting of Adverse Events (AE)

All AE shall be registered in the CRF when the investigator or study nurse becomes aware of the incident as indicated above (section 9.3, Reporting and registration of Adverse Events). All AE will also be reported to Merck according to the agreement between Merck and the sponsor. AE will also be reported to the DSMB as outlined in the DSMB charter.

9.3.2. Reporting of Serious Adverse Events (SAE)

Serious adverse events (SAE) are reported to the sponsor on a special SAE form within 24 hours of the investigator being informed of the SAE.

Follow-up information describing the outcome and handling of the SAE is reported as soon as this information is available. The original should be kept in the Investigator Site File.

The sponsor will review the SAE for SUSAR according to if the incident fulfils the requirements of an SAE, if a causal relationship is likely (section 9.2.1 Assessment of causal relationship) and if the event is unexpected (section 9.1.4 Suspected Unexpected Serious Adverse Reactions).

9.3.3. Reporting of Suspected Unexpected Serious Adverse Reactions (SUSAR)

SUSAR that are fatal or life-threatening are reported as soon as possible and no later than 7 days after the serious adverse event has become known to the sponsor. Relevant follow-up information is sent thereafter within an additional 8 days. Other SUSAR are reported as soon as possible and no later than 15 days after they have come to the sponsor's knowledge.

Information about SUSAR occurring during the trial is compiled by the sponsor and sent to the principal investigators at all participating sites.

As far as possible, study personnel will remained blinded to groups.

Since this is an investigator-initiated non-commercial trial where the sponsor representant lacks the ability to report directly into the EudraVigilance database, reporting of SUSARs for all Nordic sites will be handled by Department of Clinical Pharmacology at Sahlgrenska University hospital, Sweden.

9.3.4 Reporting of Adverse Event of Special Interest (AESI)

AESI will be reported as a SUSAR according to 9.3.3.

9.4. Follow-up of Adverse Events

All reported incidents that have not been resolved at the last visit will be followed up by a telephone call 30 days after the last visit. All AEs must be followed up until they are fully evaluated or no longer considered clinically significant by the investigator. In case of unacceptable AEs, treatment interruption and removal of subject from the trial must be considered. For SAEs, the investigator will follow the participant until the SAE has subsided, or until the condition becomes chronic in nature, or stabilizes. Within 24 hours of receipt of follow-up information, the investigator shall update the SAE form and submit any supporting documentation to the sponsor via mail.

9.5. Independent Data Monitoring Committee

The Independent Data Monitoring Committee will have meetings every 12 months to discuss the progress of the trial. They will be supplied with reports of progress prior to each meeting. They will be notified as per the protocol of all serious adverse effects. If there are serious concerns, they will arrange an emergency meeting and they will be able to stop the trial at any time. Minutes of their meetings will be sent to the chair of the PI4 Steering Committee for documentation and auditing purposes. An attachment describing the the work of the DSMB is attached to this protocol (Attachment F).

9.6. Annual Safety Report (ASR)

A yearly safety report will be submitted by the sponsor in CTIS. The report will be written in the format "Development Safety Update Report (DSUR). In the safety report, time frame will be defined as well as a list of all SAE and SUSAR. In addition, a summary of the safety for the research participants and benefit/risk assessment for the trial will be submitted as an attachment. The report will only contain aggregated and anonymised data.

9.7. Procedures in case of emergencies or overdose

Misuse of the trial medication will be reported in the same manner as adverse reactions in the CRF. The trial might be temporarily suspended or there might be supplementary monitoring measures introduced if an unforeseen event is likely to have a serious impact on the benefit/risk relationship.

9.8. Reference Safety Information

Description of Metformin is outlined in SmPC UK (Attachment E)

10. Statistics

10.1. Analysis populations

The Intention-To-Treat (ITT) population consists of all randomised participants.

The Per-Protocol population consists of all participants in the ITT-population without any major protocol deviations. The per-protocol-population is defined at the clean-file meeting before the database is locked.

The safety population consists of all participants who received at least one dose of study medication.

10.2. Statistical methods

General statistical methodology

Descriptive data will be presented as mean and standard deviation, mean and coefficient of variation, or median and interquartile range for numeric variables. Categorical variables will be presented using numbers and percentages. All baseline variables will be tabulated and presented descriptively by treatment group.

An appropriate analysis of the difference between treatments with regards to non-normally distributed primary outcome prolongation of gestation and secondary outcome neonatal birthweight will be performed using log-normal mixed effects models. Due to the presence of zeroes and competing events, length of neonatal stay will be analysed using competing risk analysis with stillbirth and neonatal death as competing events. Treatment group and gestational age strata (22+0–28+6 or 29+0–33+6 weeks at inclusion) will be included as fixed effects. Confinement hospital will be included as random effect in mixed effects models, and as fixed effect in time-to-event analyses. The treatment effect will be given as fold-change between metformin ER and placebo arms.

Exploratory outcomes will be analysed using Welch's T-test for continuous variables, possibly after application of appropriate transformations (e.g., log-transformation for positive, skewed variables). Questionnaire scales will be treated as continuous variables in this regard. Binary variables will be analysed using the Farrington-Manning test for the rate difference (risk difference). Rate ratios (relative risks) will be presented descriptively. Ordinal variables will be analysed using the Mantel-Haenszel chi-squared test, and non-ordered categorical variables using Pearson's chi-squared test.

All primary, secondary, and exploratory endpoints will be analysed on the ITT and PP populations. Safety analyses will be performed on the safety population.

All tests will be two-tailed and conducted at 5% significance level. All estimates of treatment effect will be presented with 95% confidence intervals. The type I error rate of the primary and secondary outcomes will be maintained using a hierarchical testing procedure.

Primary efficacy analysis

The difference in prolongation of gestation between the metformin ER and placebo group will be analysed on the ITT population using a log-normal mixed effects censored regression model. Treatment group and gestational age strata (22+0–28+6 or 29+0–33+6 weeks at inclusion) will be included as fixed effects, and confinement hospital as random effect. Truncated observation times due to induction of delivery at 37 weeks of gestation, without other maternal or foetal indications for delivery, will be considered as right censored observations. Prolongation of gestation in pregnancies with intrauterine foetal demise will be considered as interval censored between time from randomisation to last visit preceding

delivery (where the baby was known to be alive) and time from randomisation to delivery. The treatment effect will be given as the fold-change between metformin ER and placebo arms with 95% confidence interval, calculated as the exponentiated regression coefficient of the group effect on log-scale. The primary efficacy analysis will be performed at the 5% significance level.

Sensitivity analyses to assess the impact of stillbirths will be performed by

i) considering prolongation of gestation in pregnancies will intrauterine foetal demise as left censored at the smallest observed prolongation in pregnancies with a live born baby, and
ii) treating stillborn babies as having zero prolongation, analysed using a zero-inflated log-normal mixed effects model.

Sensitivity analyses using Kaplan-Meier analysis and Cox proportional hazards regression will also be performed.

Secondary efficacy analyses

Secondary efficacy analyses are the comparison of length of neonatal stay and birthweight between metformin ER and placebo group on the ITT population, as follows:

Length of neonatal stay will be analysed using the Fine-Gray subdistribution hazard model, with stillbirth and neonatal death as competing events. With this method stillborn babies and babies that die during neonatal care will remain in the risk set also after death and hence contribute to a longer length of neonatal stay.Treatment group, gestational age strata (22+0–28+6 or 29+0–33+6 weeks at inclusion) and confinement hospital will be included as fixed effects. The treatment effect will be estimated as the ratio of the median length of neonatal stay between treatment groups, marginalised over stratification variables. Standard error and confidence interval will be calculated using non-parametric bootstrap, and p-value calculated using Wald Z-test. A significant difference in length of neonatal stay between treatment groups will be considered to be a confirmatory finding if both this and the primary endpoint are significant at the 5% level. For descriptive purposes, differences and ratios in other percentiles will also be presented. Sensitivity analyses using cause-specific Cox proportional-hazards regression will also be performed, and corresponding cause-specific hazard ratios presented. The proportional hazards assumption will be assessed using standard diagnostics. Sensitivity analyses to account for the possibility of non-proportional hazards and informative censoring will be performed, as appropriate.

Neonatal birthweight will be analysed using a log-normal mixed effects model, with treatment group and gestational age strata (22+0–28+6 or 29+0–33+6 weeks at inclusion) as fixed effects, and confinement hospital as random effect. The treatment effect will be given by the fold-change between metformin ER and placebo arms with 95% confidence interval, calculated as the exponentiated regression coefficient of the group effect on log-scale. A significant difference in neonatal birthweight between treatment groups will be considered to be a confirmatory finding if both this test and the primary and first secondary endpoints are significant at the 5% level. For descriptive purposes, the mean difference will also be calculated using linear mixed effects model with normal distribution, identity link, and robust standard errors.

Exploratory analyses

Exploratory outcomes will be analysed according to the methods described under “General statistical methodology” above. Exploratory analyses, subgroup analyses and interaction analyses will be performed to assess compliance, dose-dependence, missing data, and treatment effect heterogeneity.

Cost-effectiveness analysis

The cost-effectiveness analysis will first assess whether the intervention is either dominating or being dominated by the standard care, i.e., if the intervention is either more beneficial with regards to costs and health outcomes, or less beneficial on both accounts. In both those cases, the decision to recommend or not the new treatment can be made without further analysis. However, if either of the treatments results in both higher costs and better health outcomes, the incremental cost-effectiveness ratio will be calculated. Sensitivity analyses will be conducted according to state of the art to account for mortality as well as skewness, assumptions, and uncertainty, including time horizon, costs, and health outcomes.

Safety analyses

Safety analyses will be performed on the safety population. In safety analyses, a subject will belong to the actual treatment taken, not to the randomised treatment.

Statistical Analysis Plan

A Statistical Analysis Plan that contains a detailed description of all planned analysis will be written and signed before the database is locked. Once all study data has been collected and entered, the database will be reviewed for completeness, accuracy, and consistency. At a formal Clean File meeting the database will be declared locked, and the analysis can start.

Drop-outs

We don’t expect any missing data for the primary efficacy analysis since all necessary data will be collected at enrolment or can be retrieved from medical charts. All randomised women will be included in the primary efficacy analysis on an intention-to-treat basis.

10.3. Adjustment of significance and confidence interval

The type I error rate of the primary and secondary outcomes will be maintained using a hierarchical testing procedure. In case of a successful test for the primary outcome, the entire probability mass $\alpha=0.05$ will be transferred to the secondary outcomes, one by one, in the order listed above. Statistical significance will be declared for all outcomes with a significant result until the first encounter of a non-significant test. All these significant tests will be considered to be confirmatory findings. Estimates and confidence intervals of remaining outcomes will be presented descriptively but not considered as confirmatory findings. Exploratory outcomes will be presented with point estimates and unadjusted 95% confidence limits only.

10.4. Sample size calculations

A total of 294 women will be recruited to this study, as needed to detect a 50% increase in the primary endpoint prolongation of gestation between the metformin ER and placebo arms at 90% power, two-sided test, 5% significance level. Based on data from the Swedish observational study GO PROVE [36] underlying assumptions are a coefficient of variation of 1.35, median 8 days prolongation in the placebo arm, 5% censored observations in the placebo arm, and log-normal distribution.

Due to the possibility of censoring in the primary outcome and the influence of gestational age at inclusion on the probability of censoring, the sample size was estimated by means of simulations from a pilot sample of 46 women in the GO PROVE study being managed expectantly with preeclampsia diagnosed before 34 gestational weeks. A total sample size of 280 was found necessary. To be cautious, the sample size was uplift by 5% to a total of 294 women. Sample size calculations were performed using SAS/STAT Software, Version 9.4 of the SAS System for Windows (SAS Institute, Cary, NC, USA).

10.5. Interim analysis (if relevant)

No interim analyses are planned.

11. Quality Control and Quality Assurance

11.1. Quality Assurance and Sponsor oversight

The sponsor representative shall maintain quality assurance throughout the trial; design, implementation, data collection, evaluation, reporting, and archiving, to ensure that the trial is conducted and data are generated, documented, monitored, reported and archived in compliance with the protocol, Good Clinical Practice (GCP), applicable requirements of the organization, Clinical Trials Regulation EU 536/2014 and other applicable regulatory requirement(s). The following quality assurance systems to ensure and control the quality shall be used: training of trial personnel, working manuals, regular meetings, central monitoring and audits.

To enable monitoring and auditing, the investigators shall allow trial-related monitoring, auditing, and regulatory inspections by providing direct access to the eCRF, patient record, as well as other source data and other study-specific documentation. Similarly, this shall also be apparent to the subjects in the subject information and ICF. The sponsor representative/PI shall be responsible for the trial’s monitoring plan, which shall be based on the identified risks, as well as follow-up of risks during the trial, as well as timeliness of the monitoring plan.

11.2. Monitoring

The trial will be monitored by an independent monitor before the trial begins, during conducting the trial, and after the trial has been completed. This is to ensure that the trial is carried out according to the protocol and that data is collected, documented, and reported according to ICH-GCP and applicable ethical and regulatory requirements. Monitoring is performed as per the trial’s monitoring plan and is intended to ensure that the subject’s

rights, safety, and well-being are met and that data in the CRF are complete, correct, and consistent with the source data.

11.3. Source data

The investigator must keep source documents for each subject in the trial. A document describing what has been classified as source data in the trial (source data reference document) should be included in the Investigator Site File (ISF). The investigator must ensure that all source documents are accessible for monitoring and other quality control activities.

Source data are defined before trial start at each individual site and can, in cases where source data is not registered in another document, consist of the CRF. This should be decided in consultation with the monitor and clearly stated in the source data reference document.

Access to trial-related documentation, such as subjects' medical records, CRFs, other source data and other trial documentation will be provided for monitoring and auditing purposes. Access to subjects' medical records will require a confidentiality agreement to be signed by the person in charge of the medical records at the trial site (the head of department) and by the monitor and auditor, if applicable. Access will also be granted in the context of regulatory inspections.

11.4. Deviations, serious breaches and other reporting obligations

The responsible investigator shall, without delay, report to the sponsor any serious breaches and deviations from the trial protocol, ICH-GCP and other regulations that significantly and directly affect, or with high likelihood could affect, the subjects’ safety and integrity or the reliability and robustness of the data generated in the trial. The sponsor should assess the suspected serious breach and the consequences of deviations that have occurred, and, without undue delay but no later than 7 days (from knowledge) report these to the Medical Products Agency via CTIS.

Other unexpected events that may affect the benefit/risk relationship must be reported via CTIS without undue delay, but no later than 15 days after the sponsor becomes aware of the event.

Minor deviations that do not affect subjects’ integrity or safety, nor significantly affect the trial’s scientific value, are documented in the trial documentation of the principal investigator and the sponsor and appropriate measures shall be taken. The deviations must be recorded in the clinical trial report.

11.5. Audits and inspections

Authorized representatives for the sponsor and Competent Authorities (CA) may carry out audits or inspections at the trial site, including source data verification. The investigator must ensure that all source documents are available for audits and inspections. The purpose of an audit or inspection is to systematically and independently review all trial-related activities and

documents, to determine whether these activities were performed, registered, analysed and reported correctly according to protocol, ICH- GCP and applicable regulations.

12. Ethics

12.1. Compliance to the protocol, ICH-GCP and regulations

The trial will be performed in compliance with this clinical trial protocol, the EU regulation on clinical trials on medicinal products for human use (536/2014), the Declaration of Helsinki, ICH-GCP E6(R3) (Good Clinical Practice), and current national regulations governing this clinical trial. This is to ensure the safety and integrity of the trial subjects as well as the quality of the data collected.

12.2. Ethical review of the trial

The final protocol for clinical trials on medicinal products must be approved, as a part of the application for a permit for clinical trials via CTIS, by the Ethical Review Authority and the Medical Products Agency in alla participating countries before the trial can be conducted. The final version of the informed consent form and other information provided to subjects, must be approved or given a written positive opinion by the Ethical Review Authorities. The authorities must be informed via CTIS of any changes in the trial protocol in accordance with current requirements. See also section 13, Substantial changes to the .

12.3. Procedure for obtaining informed consent

The principal investigator at each site shall ensure that the subject is given full and adequate oral and written information about the trial, its purpose, any risks and benefits as well as inclusion and exclusion criteria. Subjects must also be informed that they are free to discontinue their participation in the trial at any time without having to provide a reason. Subjects should be given the opportunity to ask questions and be allowed time to consider the provided information. If the person chooses to participate, both the subject and the investigator shall sign the informed consent form. A copy of the subject information as well as the informed consent form shall be provided to the subject. The subject's signed and dated informed consent must be obtained before any trial-specific activity is performed. Each subject who participates in the trial will be identified by a subject number on a subject identification list. The subject agrees that monitors, auditors, and inspectors may have access to their medical records and other source data. If new information is added to the trial, the subject has the right to reconsider whether he/she will continue their participation. Informed consent from the legal guardian or guardians regarding follow up data of the child and potential analyses of the umbilical cord sample taken at delivery will be obtained after the child is born.

12.4. Data protection

If any part of the data is processed by another organization, inside or outside the EU, appropriate agreements and/or other appropriate protective measures are taken to ensure that the data processing is performed in accordance with the provisions of the General Data

Protection Regulation (GDPR) and other relevant legislation, before any data transfer takes place.

In the information provided to subjects, subjects will be fully informed about how their trial data will be collected, used and disclosed. The content of the informed consent form complies with relevant integrity and data protection legislation. The subject information and the informed consent form will explain how trial data are stored to maintain confidentiality in accordance with national data legislation. Data will be stored in the online double authentication eCRF REDCap, provided by Västra Götaland region. Data recorded in the treatment diary through the mobile application MyCap is directly transferred to the eCRF in REDCap. All data will be pseudonymized. Each participant will be assigned a unique participant study number in REDCap. The code to the personal identification number for the participant will be kept in a closed locker.

The informed consent form will also explain that for verification of the data, representatives delegated by the sponsor, as well as relevant authorities, may require access to parts of medical records or trial records that are relevant to the trial, including the subject's medical history.

12.5. Insurances

Swedish participants are insured through the Swedish patient insurance. The Swedish pharmaceutical insurance is valid for metformin ER. In Finland trial participants are insured via patient insurance and regarding metformin via Finnish pharmaceutical insurance. In Norway trial participants are insured through the Norwegian Drug Liability Insurance Association (LAF).

13. Substantial changes to the trial

Substantial changes to the signed clinical trial protocol are only possible through approved protocol amendments and by agreement between the sponsor and the principal investigator.

In the event that substantial changes to the protocol which may affect the safety, rights of subjects or the reliability and robustness of data generated need to be implemented during the course of the trial, permission from the relevant authority via application in CTIS should be obtained before implementing the change. This includes the addition of a new trial site or a change of the principal investigator at the trial site.

Non-substantial changes should be continuously recorded and entered in CTIS.

14. Data governance

Subjects who participate in the trial are coded with a trial-specific identification number. All subjects are registered in a subject identification list (subject enrolment and identification list) that connects the subject's name and personal number with a subject number/trial identification number.

All data will be registered, managed, and stored in a manner that enables correct reporting, interpretation, and verification. The complete Trial Master File, as well as source documents, will be archived for at least *25* years after the trial is completed. Source data in the medical records system are stored and archived in accordance with national regulations.

14.1. Case Report Form

An electronic Case Report Form (eCRF) is used for data collection. The eCRF system REDCap that is used for this trial is provided by Västra Götalands region. The investigator must ensure that data is registered and any corrections in the eCRF are made as stated in the clinical trial protocol and in accordance with the instructions. The investigator must ensure that the registered data is correct, complete, and that reporting takes place according to the timelines that have been predefined and agreed. The investigator signs the completed CRF. A copy of the completed eCRF will be archived at the study site.

15. Notification of trial completion, reporting, and publication

End of the trial is reported in CTIS at the latest 15 days after completion.

Within one year of trial completion, a clinical study report is completed, and a summary of the clinical trial results must be reported in CTIS, including a summary for lay people.

We plan to publish the results in an international peer-reviewed journal.

16. References

1. Chappell, L.C., et al., *Pre-eclampsia.* Lancet, 2021. **398**(10297): p. 341-354.
2. Abalos, E., et al., *Global and regional estimates of preeclampsia and eclampsia: a systematic review.* Eur J Obstet Gynecol Reprod Biol, 2013. **170**(1): p. 1-7.
3. Cluver, C.A., et al., *Use of metformin to prolong gestation in preterm pre-eclampsia: randomised, double blind, placebo controlled trial.* BMJ, 2021. **374**: p. n2103.
4. Brownfoot, F.C., et al., *Metformin as a prevention and treatment for preeclampsia: effects on soluble fms-like tyrosine kinase 1 and soluble endoglin secretion and endothelial dysfunction.* Am J Obstet Gynecol, 2016. **214**(3): p. 356 e1-356 e15.
5. Hu, J., J. Zhang, and B. Zhu, *Protective effect of metformin on a rat model of lipopolysaccharide-induced preeclampsia.* Fundam Clin Pharmacol, 2019. **33**(6): p. 649-658.
6. Wang, F., et al., *Effect of Metformin on a Preeclampsia-Like Mouse Model Induced by High-Fat Diet.* Biomed Res Int, 2019. **2019**: p. 6547019.
7. Buse, J.B., et al., *The Primary Glucose-Lowering Effect of Metformin Resides in the Gut, Not the Circulation: Results From Short-term Pharmacokinetic and 12-Week Dose-Ranging Studies.* Diabetes Care, 2016. **39**(2): p. 198-205.
8. Shin, N.R., et al., *An increase in the Akkermansia spp. population induced by metformin treatment improves glucose homeostasis in diet-induced obese mice.* Gut, 2014. **63**(5): p. 727-35.
9. Forslund, K., et al., *Disentangling type 2 diabetes and metformin treatment signatures in the human gut microbiota.* Nature, 2015. **528**(7581): p. 262-266.
10. de la Cuesta-Zuluaga, J., et al., *Metformin Is Associated With Higher Relative Abundance of Mucin-Degrading Akkermansia muciniphila and Several Short-Chain Fatty Acid-Producing Microbiota in the Gut.* Diabetes Care, 2017. **40**(1): p. 54-62.
11. Wu, H., et al., *Metformin alters the gut microbiome of individuals with treatment-naive type 2 diabetes, contributing to the therapeutic effects of the drug.* Nat Med, 2017. **23**(7): p. 850-858.
12. Forslund, S.K., et al., *Combinatorial, additive and dose-dependent drug-microbiome associations.* Nature, 2021. **600**(7889): p. 500-505.
13. Todros, T., et al., *Increased levels of macrophage migration inhibitory factor (MIF) in preeclampsia.* Eur J Obstet Gynecol Reprod Biol, 2005. **123**(2): p. 162-6.
14. Bao, L.X., W.T. Shi, and Y.X. Han, *Metformin versus insulin for gestational diabetes: a systematic review and meta-analysis.* J Matern Fetal Neonatal Med, 2021. **34**(16): p. 2741-2753.
15. Kalafat, E., et al., *Metformin for prevention of hypertensive disorders of pregnancy in women with gestational diabetes or obesity: systematic review and meta-analysis of randomized trials.* Ultrasound Obstet Gynecol, 2018. **52**(6): p. 706-714.
16. Cluver, C., et al., *A double blind, randomised, placebo-controlled trial to evaluate the efficacy of metformin to treat preterm pre-eclampsia (PI2 Trial): study protocol.* BMJ Open, 2019. **9**(4): p. e025809.
17. Gilbert, C., M. Valois, and G. Koren, *Pregnancy outcome after first-trimester exposure to metformin: a meta-analysis.* Fertil Steril, 2006. **86**(3): p. 658-63.
18. Rowan, J.A., et al., *Metformin versus insulin for the treatment of gestational diabetes.* N Engl J Med, 2008. **358**(19): p. 2003-15.

19. Coetzee, E.J. and W.P. Jackson, *Pregnancy in established non-insulin-dependent diabetics. A five-and-a-half year study at Groote Schuur Hospital.* S Afr Med J, 1980. **58**(20): p. 795-802.
20. Hughes, R.C. and J.A. Rowan, *Pregnancy in women with Type 2 diabetes: who takes metformin and what is the outcome?* Diabet Med, 2006. **23**(3): p. 318-22.
21. Charles, B., et al., *Population pharmacokinetics of metformin in late pregnancy.* Ther Drug Monit, 2006. **28**(1): p. 67-72.
22. Cassina, M., et al., *First-trimester exposure to metformin and risk of birth defects: a systematic review and meta-analysis.* Hum Reprod Update, 2014. **20**(5): p. 656-69.
23. Barbour, L.A., et al., *A cautionary response to SMFM statement: pharmacological treatment of gestational diabetes.* Am J Obstet Gynecol, 2018. **219**(4): p. 367 e1-367 e7.
24. MaHpR, A., *Drug Safety Update 2022*. 2022.
25. Landi, S.N., et al., *Association of Long-term Child Growth and Developmental Outcomes With Metformin vs Insulin Treatment for Gestational Diabetes.* JAMA Pediatr, 2019. **173**(2): p. 160-168.
26. *Practice Bulletin No. 180: Gestational Diabetes Mellitus.* Obstet Gynecol, 2017. **130**(1): p. e17-e37.
27. Fadl, H., et al., *Changing diagnostic criteria for gestational diabetes in Sweden - a stepped wedge national cluster randomised controlled trial - the CDC4G study protocol.* BMC Pregnancy Childbirth, 2019. **19**(1): p. 398.
28. Choi, Y.H., S.G. Kim, and M.G. Lee, *Dose-independent pharmacokinetics of metformin in rats: Hepatic and gastrointestinal first-pass effects.* J Pharm Sci, 2006. **95**(11): p. 2543-52.
29. Song, I.H., et al., *The Effect of Dolutegravir on the Pharmacokinetics of Metformin in Healthy Subjects.* J Acquir Immune Defic Syndr, 2016. **72**(4): p. 400-7.
30. Te Brake, L.H.M., et al., *Rifampicin Alters Metformin Plasma Exposure but Not Blood Glucose Levels in Diabetic Tuberculosis Patients.* Clin Pharmacol Ther, 2019. **105**(3): p. 730-737.
31. Tong, S., et al., *Clinical trial of metformin to treat preterm preeclampsia: pharmacokinetics and biomarker studies.* Pregnancy Hypertension, 2018. **13**: p. S97.
32. Marsal, K., et al., *Intrauterine growth curves based on ultrasonically estimated foetal weights.* Acta Paediatr, 1996. **85**(7): p. 843-8.
33. Wang, C., et al., *Literature review of the clinical characteristics of metformin-induced hepatotoxicity.* Front Pharmacol, 2022. **13**: p. 969505.
34. Brackett, C.C., *Clarifying metformin's role and risks in liver dysfunction.* J Am Pharm Assoc (2003), 2010. **50**(3): p. 407-10.
35. Jalali, M., et al., *The effects of metformin administration on liver enzymes and body composition in non-diabetic patients with non-alcoholic fatty liver disease and/or non-alcoholic steatohepatitis: An up-to date systematic review and meta-analysis of randomized controlled trials.* Pharmacol Res, 2020. **159**: p. 104799.
36. Thorgeirsdottir, L., et al., *Study protocol: establishment of a multicentre pre-eclampsia database and biobank in Sweden: GO PROVE and UP MOST, a prospective cohort study.* BMJ Open, 2021. **11**(11): p. e049559.

17. Attachments

Attachment A:

Treatment Diary

Attachment B:

PHASE-20 – PHArmacotherapeutical Symptom Evaluation scale

Attachment C

Variable list for Swedish and Norwegian registers

Attachment D:

Edinburgh Postnatal Depression Scale (EPDS)

Rosenberg Self-Esteem Scale (RSE)

Perceived Stress Scale (PSS)

Breastfeeding self-efficacy scale short-form (BFS)

Health-related quality of life (EQ-5D)

Postpartum Bonding Questionnaire (PBQ)

Breastfeeding Questions

Modified Checklist for Autism in Toddlers, Revised (M-CHAT-R)

The Ages and Stages Questionnaire, Third Edition (ASQ) 24-month questionnaire

Attachment E:

SmPC Metformin extended release

Attachment F:

DSMB Charter

Attachment G:

Satellite sites

Attachment H:

Dosage procedures and treatment responsibility for Finland
