# Supplementary material for: Can metformin prolong pregnancy in preterm pre-eclampsia? Study protocol for a Nordic multicentre randomised placebo-controlled trial (PI4)
Source: BMJ Open. 2026 Oct 13;16(10):e120993. doi: 10.1136/bmjopen-2026-120993 (PMC13663727; doi:10.1136/bmjopen-2026-120993)
Supplement: Supplementary file 2 [file bmjopen-16-10-s003.pdf]

# Online Supplemental Appendix 3

## Validity and reliability of study instruments in the PI4 trial

*PHASE-20*

PHASE-20 was developed and evaluated for identifying possible medication-related symptoms in older adults (Hedström et al., 2009); responsiveness has recently been demonstrated for an adapted version in adults (Mekuria et al., 2026), but has not been evaluated in pregnant women.[1,2]

*The Breastfeeding Self-Efficacy Scale–Short Form (BSES-SF)*

The Swedish version of the Breastfeeding Self-Efficacy Scale–Short Form (BSES-SF) has demonstrated good reliability and validity in postpartum women.[3]

*The Edinburgh Postnatal Depression Scale (EPDS)*

The Swedish version of the Edinburgh Postnatal Depression Scale (EPDS) has demonstrated good validity for detecting depressive symptoms both during pregnancy and postpartum.[4,5]

*The EQ-5D-5L*

The EQ-5D-5L has demonstrated validity and responsiveness across a range of adult populations and has an established Swedish value set.[6,7]

*The Postpartum Bonding Questionnaire (PBQ)*

The Postpartum Bonding Questionnaire (PBQ) has demonstrated good validity for assessing disturbances in parent–infant bonding and has shown acceptable internal reliability in Swedish postpartum populations.[8,9]

*Perceived Stress Scale (PSS-10)*

The Swedish version of the 10-item Perceived Stress Scale (PSS-10) has demonstrated good reliability and construct validity in a general adult population.[10]

*The Rosenberg Self-Esteem Scale (RSES)*

The Swedish version of the Rosenberg Self-Esteem Scale (RSES) has demonstrated satisfactory reliability, validity, and sensitivity to change in adult populations.[11]

*Neurodevelopmental tests*

The choice of neurodevelopmental test instruments is based on clinical and scientific use in neonatal risk groups, including preterm infants, at the age specified in the program (2 years corrected age). Bayley-4 is included in the Swedish National Follow-up Program for high-risk infants [12] and is widely used in studies evaluating neurodevelopmental outcome in preterm infants.[13] The validity and reliability are summarized in reference 14. M-CHAT-R/F is used in screening for autism in the general population as well as in preterm infants with adequate sensitivity and specificity.[15] Test data is

summarized in reference 16. Ages and Stages Questionnaire is internationally used for developmental screening of low-risk as well as high-risk infants including preterms.[17]

**References**


1. Hedström, M., Lidström, B., & Hulter Åsberg, K. (2009). PHASE-20: Ett nytt instrument för skattning av möjliga läkemedelsrelaterade symtom hos äldre personer i äldreboende. Vård i Norden, 29(4), 9–14.
2. Mekuria, A. B., Andrade, A. Q., Lim, R., Rowett, D., Hedström, M., & Roughead, E. E. (2026). Responsiveness and minimum important change of the Pharmacotherapeutic Symptom Evaluation-20–Australian version: A tool for measuring changes in medicine-related symptoms over time. International Journal of Clinical Pharmacy, 48, 644–655. https://doi.org/10.1007/s11096-025-02045-4.
3. Gerhardsson, E., Nyqvist, K. H., Mattsson, E., Volgsten, H., Hildingsson, I., & Funkquist, E.-L. (2014). The Swedish version of the Breastfeeding Self-Efficacy Scale–Short Form: Reliability and validity assessment. Journal of Human Lactation, 30(3), 340–345. https://doi.org/10.1177/0890334414523836.
4. Wickberg, B., & Hwang, C. P. (1996). The Edinburgh Postnatal Depression Scale: Validation on a Swedish community sample. Acta Psychiatrica Scandinavica, 94(3), 181–184. https://doi.org/10.1111/j.1600-0447.1996.tb09845.x.
5. Rubertsson, C., Börjesson, K., Berglund, A., Josefsson, A., & Sydsjö, G. (2011). The Swedish validation of Edinburgh Postnatal Depression Scale (EPDS) during pregnancy. Nordic Journal of Psychiatry, 65(6), 414–418. https://doi.org/10.3109/08039488.2011.590606.
6. Feng, Y.-S., Kohlmann, T., Janssen, M. F., & Buchholz, I. (2021). Psychometric properties of the EQ-5D-5L: A systematic review of the literature. Quality of Life Research, 30, 647–673. https://doi.org/10.1007/s11136-020-02688-y
7. Burström, K., Teni, F. S., Gerdtham, U.-G., Leidl, R., Helgesson, G., Rolfson, O., & Henriksson, M. (2020). Experience-based Swedish TTO and VAS value sets for EQ-5D-5L health states. PharmacoEconomics, 38, 839–856. https://doi.org/10.1007/s40273-020-00905-7.
8. Brockington, I. F., Fraser, C., & Wilson, D. (2006). The Postpartum Bonding Questionnaire: A validation. Archives of Women's Mental Health, 9, 233–242.
9. Lindeberg, S., Tedgård, E., Kerstis, B., Tedgård, U., Taylor, A., & Jönsson, P. (2026). Development of the Parent-to-Infant Bonding Scale: validation in Swedish mothers and fathers in community and clinical contexts. Child Psychiatry & Human Development, 57(2), 324-336.
10. Nordin, M., & Nordin, S. (2013). Psychometric evaluation and normative data of the Swedish version of the 10-item Perceived Stress Scale. Scandinavian Journal of Psychology, 54(6), 502–507. https://doi.org/10.1111/sjop.12071.
11. Eklund, M., Bäckström, M., & Hansson, L. (2018). Psychometric evaluation of the Swedish version of Rosenberg's self-esteem scale. Nordic Journal of Psychiatry, 72(5), 318–324. https://doi.org/10.1080/08039488.2018.1457177.
12. National Guidelines for the Follow-up of Neonatal Risk Infants. Swedish Neonatal Society 2015. https://neo.barnlakarforeningen.se/wp-content/uploads/sites/14/2022/10/Nationella-Uppfoljningsprogrammet-2015.pdf,
13. Serenius F, Källén K, Blennow M, Ewald U, Fellman V, Holmström G, Lindberg E, Lundqvist P, Maršál K, Norman M, Olhager E, Stigson L, Stjernqvist K, Vollmer B, Strömberg B; EXPRESS Group. Neurodevelopmental outcome in extremely preterm infants at 2.5 years after active perinatal care in Sweden. JAMA 2013 309(17):1810-20.
14. Bayley, N., & Aylward, G.P Bayley-4: Scales of Infant and Toddler Development: Technical manual. NCS Pearson, Inc. 2019.
15. Hamner T, Perez Liz G, Kelly K, Nanovic S, Turchi R, Fein D, Robins DL. Autism screening and diagnostic outcomes among toddlers born preterm. Dev Med Child Neurol. 2025 67(3):348-356.

16. Robins D, Fein D, Barton M. Modifierad checklista för autism hos små barn, reviderad med uppföljning (M-CHAT-R/F). https://mchatscreen.com/wp-content/uploads/2015/05/M-CHAT-R_F_Swedish.pdf.
17. Schonhaut L, Armijo I, Schönstedt M, Alvarez J, Cordero M. Validity of the ages and stages questionnaires in term and preterm infants. Pediatrics. 2013 131(5):e1468-74.
