# Supplementary material for: Can metformin prolong pregnancy in preterm pre-eclampsia? Study protocol for a Nordic multicentre randomised placebo-controlled trial (PI4)
Source: BMJ Open. 2026 Oct 13;16(10):e120993. doi: 10.1136/bmjopen-2026-120993 (PMC13663727; doi:10.1136/bmjopen-2026-120993)
Supplement: Supplementary file 3 [file bmjopen-16-10-s004.pdf]

Form 1 - Inclusion And Exclusion Criteria

Participant Study Number

____

Date informed consent signed (consent 1)

____

Written consent for follow-up study on child (consent 2), caregiver 1 - the response should be moved to Form 7 - Delivery details. To remove the response, click on "Reset". After this, the field will be hidden

◯ Yes ◯ No

Written consent for follow-up study on child (consent 2), caregiver 2 - the response should be moved to Form 7 - Delivery details. To remove the response, click on "Reset". After this, the field will be hidden

◯ Yes ◯ No ◯ Not applicable (single caregiver)

Date of Birth

____
(YYYY-MM-DD)

Age

____
(Age)

Estimated due date

____
(YYYY-MM-DD)

Pregnancy dated by

◯ First trimester ultrasound
◯ Second trimester ultrasound
◯ Last menstrual period
◯ Embryo Transfer (ET)
◯ Unsure dates

Gestational status at inclusion [ga_incl_w_calc] wks +

[ga_incl_d_calc] days

If the gestational age is between 22+0 - 23+6 gestational weeks, the sponsor should be contacted for discussion around viability before randomization

Gestation at enrolment (weeks)

____

Gestation at enrolment (days)

____

Due to copyright, questionnaires are not included

Inclusion criteria

Albumin/creatinine ratio ________________________

Protein/creatinine ratio ________________________

Urine dipstick (maximum)
- ◯ Clear
- ◯ Trace
- ◯ 1+
- ◯ 2+
- ◯ 3+

Albumin/creatinine ratio < 8 / Protein/creatinine ratio < = 30 mg/mmol and Urine dipstick below 2+ - please consider when completing inclusion criteria 1

1. Diagnosis of preeclampsia has been made by the attending clinician ◯ Yes ◯ No

Defined as: hypertension in combination with significant proteinuria (albumin/creatinine ratio ≥8 mg/mmol, protein/creatinine ratio>30 mg/mmol or >2+ protein on a urinary dipstick) with or without other organ manifestations

Date of preeclampsia diagnosis ________________________

Albumin/creatinine ratio and Protein/creatinine ratio are both not provided. Add values for one of the ratios above as soon as possible.

2. The managing clinicians have made the assessment to proceed with expectant management ◯ Yes ◯ No

3. The subject has given written consent (consent 1) to participate in the study

3. The subject has not given written consent to participate in the study - please record informed consent date above

4. The woman must be 18 years of age or older (age: [age] yrs)

4. The woman must be 18 years of age or older (age: [age] yrs)

5. The gestational age is not between 22+0 and 33+6 weeks

5. The gestational age is between 22+0 and 33+6 weeks with a viable fetus ◯ Yes ◯ No

6. The woman carries a singleton pregnancy ◯ Yes ◯ No

Exclusion criteria

1. There are contraindications to treatment with metformin as outlined in SmPC	◯ Yes ◯ No

Metformin contraindicated due to:

- ☐ Hypersensitivity to metformin or any of the excipients (magnesium stearate, sodium carboxymethylcellulose, hypromellose)
- ☐ Any type of acute metabolic acidosis (such as lactic acidosis, diabetic ketoacidosis)
- ☐ Diabetic pre-coma
- ☐ Severe renal failure (GFR < 30 mL/min)
- ☐ Acute conditions with the potential to alter renal function such as: dehydration, severe infection, shock
- ☐ Disease which may cause tissue hypoxia (especially acute disease, or worsening of chronic disease) such as: decompensated heart failure, respiratory failure, recent myocardial infarction, shock
- ☐ Hepatic insufficiency, acute alcohol intoxication, alcoholism

2. Contraindications for expectant management of preeclampsia such as an immediate indication for delivery according to Swedish guidelines	◯ Yes ◯ No
3. Type 1 Diabetes Mellitus	◯ Yes ◯ No
4. Current use of metformin	◯ Yes ◯ No
5. Known or suspected allergies against metformin	◯ Yes ◯ No
6. Reluctance or language difficulties that result in difficulty understanding the meaning of study participation	◯ Yes ◯ No
7. Unable to understand the informed consent process	◯ Yes ◯ No
8. Previous participation in the study	◯ Yes ◯ No
9. Established fetal compromise that necessitates imminent delivery (including planned delivery after 48 hours of corticosteroid treatment). This will be decided by the clinical team before expectant management is offered to the patient	◯ Yes ◯ No
10. Suspicion of a major fetal anomaly or malformation	◯ Yes ◯ No
11. Renal disease or dysfunction, suggested by a creatinine level greater than or equal to 125 µmol/L or rapidly declining renal function	◯ Yes ◯ No
12. Known acute or chronic metabolic acidosis, including diabetic ketoacidosis	◯ Yes ◯ No
13. The investigator has decided the woman is not suitable for inclusion	◯ Yes ◯ No

All eligibility criteria fullfilled, patient can be included in the study

One or more eligibity criteria not fullfilled / not completed, patient cannot be included in the study

Comments form 1

Form 2 - Randomisation

Subject is eligible according to form 1 Inclusion and exclusion criteria

Subject is not eligible according to form 1 Inclusion and exclusion criteria

Please complete form 1 Inclusion and exclusion criteria before the subject can be randomised

Language - check that correct language is displayed.<br>If not, please contact the study team.	◯ svenska<br>◯ norsk<br>◯ finska<br>◯ engelska
Satellite site SU	◯ SU<br>◯ SÄS<br>◯ NÄL<br>◯ SkaS
Satellite site Uppsala	◯ Uppsala<br>◯ Västerås
Gestational age (GA) group at enrolment<br>(stratification factor):<br>(from inclusion 5: [ga_incl_w_calc]+[ga_incl_d_calc])	◯ 22+0 to 28+6<br>◯ 29+0 to 33+6<br>( )

GA category does not correspond to calculated GA at inclusion. Please correct!

Date and time of randomisation	______________________<br>(yyyy-mm-dd hh:mm)

If try to randomize the patient and you get this message:

- check the IP database (link included) for unregistered shipments

- register any new shipments for your site - if not available, order new shipment

- contact the REDCap administrator (click to open e-mail) with subject "URGENT - PI4 IP shipments registered"

Comments form 2

Form 3 - Medical and Sociodemographic History

Obstetric history

Gravidity ______
Parity ______
Number of miscarriages ______
Number of ectopic pregnancies ______
Number of molar pregnancies ______
Number of legal abortions ______

Did the participant have hypertension in a previous pregnancy? ◯ Yes ◯ No ◯ Unknown

Did the participant have preeclampsia in a previous pregnancy? ◯ Yes ◯ No ◯ Unknown

New paternity? ◯ Yes ◯ No

First pregnancy (Gravidity=1) but one or more of the previous 3 questions (hypertension / preeclampsia previous pregnancy, new paternity) is not answered "Not applicable".

Socio-demographic information

Ethnicity
◯ European
◯ Afro-carribean
◯ East Asia (China, Japan, Korea, Vietnam etc)
◯ South Asia (India, Pakistan, Indonesia, Philippines etc)
◯ The Middle East (Irak, Iran, Saudi Arabia, Egypt etc)
◯ Mixed/other
◯ Unknown

Highest education
◯ University (universitet/högskola)
◯ Upper secondary school (gymnasium)
◯ Below 12 years (grundskola)

Smoking
◯ No
◯ Quit before the pregnancy
◯ Quit during the pregnancy
◯ Currently smoking

Snuff use
◯ No
◯ Quit before the pregnancy
◯ Quit during the pregnancy
◯ Current use of snuff

Alcohol use
◯ No
◯ Quit before the pregnancy
◯ Quit during the pregnancy
◯ Current use of alcohol

Baseline Medical Information at first visit at maternity care

Date of first visit at maternity care (MVC) ______________________________

Gestational status at first visit MVC (calculated) [ga_prim_w_calc] wks +

[ga_prim_d_calc] days

Gestational status at first visit MVC (weeks) - calculated ______________________________

Gestational status at first visit MVC (days) - calculated ______________________________

Blood pressure at first visit MVC ______ / ______ mmHg

Proteinuria at first visit MVC
- ◯ Clear
- ◯ Trace
- ◯ 1+
- ◯ 2+
- ◯ 3+
- ◯ Not done

Albumin or protein to creatinine ratio elevated?
- ◯ Albumin to creatinine ratio
- ◯ Protein to creatinine ratio

Albumin/creatinine ratio ______________________________

Protein/creatinine ratio ______________________________

On hypertensive treatment at first visit MVC ◯ Yes ◯ No

Specify hypertensive treatment ______________________________

Height ______________________________ (cm)

Weight at first visit MVC ______________________________ (kg)

Corrected BMI at first visit MVC ______________________________
(Minus 4kg if measured after 14w before calculating BMI / Minus 8kg if measured ed after 28w before calculating BMI)

Medication during pregnancy

Low dose aspirin use in this pregnancy ◯ Yes ◯ No

Gestation aspirin was started

____
(Enter GA in weeks when aspirin was started (eg.14))

Calcium use in this pregnancy (not including multivitamins) ◯ Yes ◯ No

Gestation calcium was started

____
(Enter GA in weeks when calcium was started (eg.14))

Medical history - diagnoses before pregnancy

Any medical condition to record that was diagnosed before pregnancy? ◯ Yes ◯ No

Medical history condition(s) - tick all that apply

- ☐ Hypertension
- ☐ HIV
- ☐ Renal disease
- ☐ Anemia
- ☐ Diabetes type 2
- ☐ SLE
- ☐ Inflammatory bowel disease (IBD)
- ☐ Rheumatoid arthritis
- ☐ Epilepsy
- ☐ Thromboembolism
- ☐ History of depression
- ☐ Antiphospholipid syndrome
- ☐ Asthma
- ☐ Migraine
- ☐ Irritable Bowel Syndrome (IBS)
- ☐ Anxiety disorder
- ☐ Other condition(s)

Other medical condition(s):

____

Comments form 3

Form 4 - Inclusion

Blood pressure

Highest recorded blood pressure before inlusion ______ / ______ mmHg

Medications at inclusion

Any medications at inclusion	◯ Yes ◯ No
If yes, indicate which medications	☐ Iron<br>☐ Levothyroxine<br>☐ Antidepressive medication<br>☐ Antihypertensive medication<br>☐ Other
Other medications	______________________
Antihypertensive medication, start datum	______________________
Patient started hypertensive medication [med_hypertens_days] days before inclusion.	
Number of days on hypertensive treatment (at ICF date)	______________________
Antihypertensive medication, specify	☐ Labetalol<br>☐ Nifedipine<br>☐ Hydralazine<br>☐ Other
Other hypertensive medication	______________________

First dose and diary

Date and time first tablet of study drug (metformin/placebo) was taken	______________________<br>(yyyy-mm-dd hh:mm)
First dose date (date only)	______________________
How will the diary be completed?	◯ MyCap app<br>◯ Paper
Provide the participant with the paper diary and provide instructions	

- First save the form by selecting "Save & Stay"
- To open the MyCap database and add the participant study ID, click on this link: MyCap database new subject

- First save the form by selecting "Save & Stay"
- To open the MyCap database and add the participant study ID, click on this link: MyCap database new subject

- First save the form by selecting "Save & Stay"
- To open the MyCap database and add the participant study ID, click on this link: MyCap database new subject

Comments form 4

Form 7 - Delivery Details

Written consent (consent 2) for follow-up study on child, caregiver 1 — ◯ Yes ◯ No

Written consent (consent 2) for follow-up study on child, caregiver 2 — ◯ Yes ◯ No ◯ Not applicable (single caregiver)

Parent(s) did not sign consent 2, but information about the child was already collected.

Please ensure that the information is deleted and/or the cord blood sample is destructed!!

Form 7 - Delivery details

Intrauterine fetal death (IUFD) [neo_iufd]
Babies gender [sex]
Birth weight [bw]
Apgar score at 5 minutes [apgar5]
Cord blood pH [cord_ph]
Umbilical artery lactate [cord_lac]

Form 5 Routine biochemical tests and biobank samples - cord blood sample should be destroyed

Cord blood taken [f_eso]
Date and time of cord blood sample [cord_datetime]
Cord blood number of aliquots [cord_nr]

Delivery details

Date and time of Delivery — ______________________ (YYYY-MM-DD HH:MM)

Delivery date only — ______________________

Prolongation [prolongation_days] days
[prolongation_hrs] hours

Prolongation — ______________________ (days)

Prolongation (hours) — ______________________ (hours)

Gestational status at delivery (calculated) [ga_del_w_calc] wks +

[ga_del_d_calc] days

Gestation at delivery (weeks) - calculated — ______________________

Gestation at delivery (days) - calculated — ______________________

Indication for Delivery	◯ Loss of blood pressure control<br>◯ Eclampsia<br>◯ Stroke (hemorrhagic or infarct/ischemic)<br>◯ HELLP syndrome<br>◯ Placental abruption<br>◯ Heart failure<br>◯ Maternal pulmonary oedema or high suspicion of<br>◯ Fetal death (IUFD)<br>◯ Platelet count < 100, no HELLP (thrombocytopenia)<br>◯ Elevated transaminases<br>◯ Renal failure according to national definitions*<br>◯ Remaining neurological symptoms<br>◯ Remaining epigastric pain, nausea or vomitting with affected hepatic values<br>◯ Fetal distress<br>◯ Poor fetal growth<br>◯ Deterioration in fetal Doppler studies<br>◯ Reached 37 weeks<br>◯ Spontaneous preterm labour<br>◯ Renal deterioration<br>◯ Other maternal reason<br>◯ Declined further expectant management
* Sweden: Increase in P-Creatinine by > 90 µmol/L or increase in P-Creatinine by >50% (relative increase)	
* Finland: Increase in P-Creatinine > 110 µmol/l or increase in P-Creatinine by >50% (relative increase)	
* Norway: Increase in P-Creatinine > 90µmol/l)	
Intrauterine fetal death (IUFD) should be reported as SAE	
Mode of delivery	◯ Spontaneous vaginal delivery<br>◯ Induction vaginal delivery<br>◯ Failed induction needing CS<br>◯ Elective CS<br>◯ Emergency CS
Operative vaginal delivery?	◯ No<br>◯ Yes, with vacuum extraction (sugklocka)<br>◯ Yes, with obstetrical forceps (tång)
Highest systolic blood pressure after randomisation before delivery	______________________
Highest diastolic blood pressure after randomisation before delivery	______________________
Medication used before delivery	☐ Labetalol per oral<br>☐ Nifedipine long acting (Adalat Oros)<br>☐ Nifedipine short acting<br>☐ Hydralazine oral<br>☐ IV labetalol<br>☐ IV hydralazine<br>☐ None of the above

NEONATAL OUTCOMES

Neonatal outcomes may not be collected since caregiver(s) did not consent (consent 2)

Intrauterine fetal death (IUFD) ○ Yes ○ No

Babies gender ○ Male ○ Female

Birth weight (grams) ______________________

Gestational age in days ______________________

Expected weight ______________________

Growth according to Marsal (calculated) - z-score ______________________

Growth according to Marsall (calculated) procentavvikelse ______________________

Birthweight classification Marsal [bw_class_marsal_calc]

Birthweight classification Marsal ______________________

APGAR score at 5 minutes ______________________

Cord Blood (umbilical artery) pH ______________________

Umbilical artery lactate ______________________

Comments form 7

Form 5 - Routine biochemical tests and biobank samples

Date of visit

____

Report laboratory results from the latest sample taken before the 2 year follow-up

Date of sample

____
(YYYY-MM-DD)

Tick all tests that were NOT taken/analysed at this visit

- ☐ Platelets
- ☐ Haemoglobin
- ☐ ALT
- ☐ Urea
- ☐ Creatinine
- ☐ AST

Platelets

____
(number/&#181L)

Haemoglobin

____
(g/L)

ALT

____
(µkat/L)

ALAT > 2 x reference value (>1.5 µkat/L), check that AE was reported

Urea

____
(mmol/L)

Creatinine

____
(µmol/L)

Extended samples taken ◯ Yes ◯ No

Tick all tests that were NOT taken/analysed at this visit

- ☐ AST
- ☐ PK (INR)
- ☐ APTT
- ☐ Plasma albumin
- ☐ Haptoglobin
- ☐ LD
- ☐ Antithrombin

AST

____
(µkat/L)

ASAT > 2 x reference value (>1.2 µkat/L), check that AE was reported

PK (INR)

____
((ratio))

APTT

____
(sec)

Plasma albumin

____
(g/L)

Haptoglobin

____
(g/L)

LD

____
(&#181kat/L)

Antithrombin

____
(kIE/L)

Biobank samples - for freezing

Blood taken for freezing

○ Yes ○ No

Date and time of sampling

____

Type of sample(s)

☐ EDTA plasma
☐ EDTA plasma, 1x
☐ Serum
☐ LiHep plasma
☐ Buffy coat, EDTA
☐ Whole blood

EDTA plasma

Centrifugation date and time EDTA plasma sample

____

EDTA number of aliquots

____

Date and time of freezing EDTA plasma aliquots

____

Comments EDTA plasma sample (e.g. hemolysis in the tube)

____

Serum

Centrifugation date and time serum sample

____

Serum number of aliquots

____

Date and time of freezing serum aliquots

____

Comments serum sample (e.g. hemolysis in the tube)

____

LiHep plasma

Centrifugation date and time LiHep plasma sample

____

LiHep plasma number of aliquots

____

Date and time of freezing LiHep plasma aliquots

____

Comments LiHep plasma sample (e.g. hemolysis in the tube)

____

Buffy coat, EDTA

Centrifugation date and time Buffy coat, EDTA sample

____

Buffy coat, EDTA number of aliquots

____

Date and time of freezing Buffy coat, EDTA aliquots

____

Comments Buffy coat, EDTA sample (e.g. hemolysis in the tube)

____

Whole blood

Centrifugation date and time whole blood sample

____

Date and time of freezing whole blood sample

____

Comments whole blood sample (e.g. hemolysis in the tube)

____

Urine sample

Urine sample ◯ Yes ◯ No

Date and time of urine sample ______________________

Date and time of freezing urine sample ______________________

Urine sample number of aliquots ______________________

Swabs

Rectal and vaginal swab should be taken 5 days (+/ 1 day) after start of IP ([screening_randomis_arm_1][firstdose])

Rectal swab ◯ Yes ◯ No

Date and time of rectal swab ______________________

Date and time of freezing rectal swab ______________________

Rectal swab date only ______________________

Rectal swab was taken before first dose? 1=Yes 0=No ______________________

Swab was taken before first dose of IP ([screening_randomis_arm_1][st_tablet])

Swab was taken after first dose of IP ([screening_randomis_arm_1][st_tablet])

Rectal swab not taken 4-6 days after first dose of IP ([screening_randomis_arm_1][st_tablet])

Vaginal swab ◯ Yes ◯ No

Date and time of vaginal swab ______________________

Date and time of freezing vaginal swab ______________________

Vaginal swab date only ______________________

Vaginal swab taken before first dose? 1=Yes 0=No ______________________

Swab was taken before first dose of IP ([screening_randomis_arm_1][st_tablet])

Swab was taken after first dose of IP ([screening_randomis_arm_1][st_tablet])

Vaginal swab not taken 4-6 days after first dose of IP ([screening_randomis_arm_1][st_tablet])

Post delivery samples

Cerebrospinal fluid sample	◯ Yes ◯ No
Date and time of CSF sample	______________ (YYYY-MM-DD HH:MM)
CSF sample number of aliquots	______________
Cord blood taken	◯ Yes ◯ No
Date and time cord blood sample	______________
Date and time of freezing cord blood sample	______________
Cord blood number of aliquots	______________
Parent(s) did NOT sign consent 2, the cord samples should be destroyed!!!	◯ Cord blood sample destroyed
Placental samples taken	◯ Yes ◯ No
Type of placental sample taken	☐ Fresh frozen 2 tubes<br>☐ RNA later 2 tubes
Date and time of sampling - please transfer to either fresh frozen or RNA later below	______________ (YYYY-MM-DD HH:MM)
Date and time of sampling - placental frozen fresh	______________
Date and time of freezing placental fresh frozen sample	______________
Date and time of sampling - placental RNA later	______________
Date and time of freezing placental RNA later sample	______________
Comments form 5	

Form 6 - Fetal Ultrasound Findings

Ultrasound done ◯ Yes ◯ No

Date of ultrasound ________________ (YYYY-MM-DD)

Gestational status at ultrasound [ga_us_w_calc] wks + [ga_us_d_calc] days

Gestational status at ultrasound (weeks) - calculated ________________

Gestational status at ultrasound (days) - calculated ________________

Fetal weight estimation

Fetal weight ☐ Not estimated

Estimated fetal weight (grams) ________________

Gestational age in days ________________

Expected weight ________________

Growth according to Marsal (calculated) - z-score ________________

Growth according to Marsall (calculated) procentavvikelse ________________

Ultrasound growth classification Marsal is [centile_class_marsal]

Ultrasound growth classification Marsal ________________

Amniotic fluid

Single deepest pocket (SDP) ________________ (cm (1 decimal))

Amniotic fluid index (AFI) ________________ (mm)

Record SDP or AFI

Fetal doppler investigation

Fetal doppler	☐ Not done
Umbilical artery pulsitility index (PI)	______________________
BFC	◯ Class 0<br>◯ Class 1<br>◯ Class 2<br>◯ Class 3A<br>◯ Class 3B
Further Doppler examination	☐ Not done
Middle cerebral artery pulsitility index (PI)	______________________
Redistribution in MCA	◯ Yes ◯ No ◯ Not done
Cerebro-placental ratio ______ ______	
Ductus venosus a-Wave positive	◯ Yes ◯ No ◯ Not done
Ductus venosus PI ______ ______	
Uterine artery PI, right side ______ ______<br>Uterine artery notch, right side ______	
Uterine artery PI, left side ______ ______<br>Uterine artery notch, left side ______	
Comments form 6	

Form 8 - Maternal Discharge

Maternal complications	
Maternal death	◯ Yes ◯ No
Eclampsia	◯ Yes ◯ No
Pulmonary edema	◯ Yes ◯ No
Renal failure- this variable will be hidden once new variable "highest creatinine value" is completed and the response is removed (press "reset")	◯ Yes ◯ No
Highest Creatinine value after diagnosis of preeclampsia	__________ (µmol/L)
Date highest Creatinine value	__________
Renal dialysis	◯ Yes ◯ No
Cerebro-vascular event	◯ Yes ◯ No
Liver haematoma or rupture	◯ Yes ◯ No
Admission to ICU	◯ Yes ◯ No
Posterior reversible encephalopathy syndrome	◯ Yes ◯ No
Left ventricular failure	◯ Yes ◯ No
DIC	◯ Yes ◯ No
HELLP syndrome	◯ Yes ◯ No
Placental abruption	◯ Yes ◯ No
Major PPH (more than 1000 ml in the first 24 hours PP)	◯ Yes ◯ No
Thomboembolic disease (DVT or pulmonary embolism)	◯ Yes ◯ No
Blood transfusion	◯ Yes ◯ No
Thrombocytopenia- this variable will be hidden once new variable "lowest platelets value" is completed and the response is removed (press "reset")	◯ Yes ◯ No
Lowest Platelets value after diagnosis of preeclampsia	__________ (number/µL)

Date lowest Platelets value	__________________________________
Liver transaminases > 70 µg/L	◯ Yes ◯ No
Highest ALAT value after delivery - this variable will be hidden once new variable "highest value after diagnosis of preeclampsia" is completed and this value is removed	__________________________________ (µkat/L)
Highest ALAT value after diagnosis of preeclampsia	__________________________________ (µkat/L)
Date highest ALAT value	__________________________________
Highest ALAT > 2 x reference value (>1.5 µkat/L), check that AE was reported	
Highest ASAT value after delivery - this variable will be hidden once new variable "highest value after diagnosis of preeclampsia" is completed and this value is removed	__________________________________ (µkat/L)
Highest ASAT value after diagnosis of preeclampsia	__________________________________ (µkat/L)
Date highest ASAT value	__________________________________
Highest ASAT > 2 x reference value (>1.2 µkat/L), check that AE was reported	
Severe hypertension- this variable will be hidden once the response is removed (press "reset")	◯ Yes ◯ No

Blood pressure

Highest systolic blood pressure after delivery - this variable will be hidden once new variable "highest systolic blood pressure after diagnosis of preeclampsia" is completed and this value is removed	__________________________________
Highest systolic blood pressure after diagnosis of preeclampsia	__________________________________ (mmHg)
Date highest systolic blood pressure	__________________________________
Highest diastolic blood pressure post delivery (mmHg) - this variable will be hidden once new variable "highest diastolic blood pressure after diagnosis of preeclampsia" is completed and this value is removed	__________________________________

Highest diastolic blood pressure after diagnosis of preeclampsia

____
(mmHg)

Date highest diastolic blood pressure

____

Discharge

Date and time of maternal discharge

____
(YYYY-MM-DD HH:MM)

Discharge date only

____

Mothers length of stay (hours from delivery - discharge)

____

Prescribed hypertensive drug at discharge?

◯ Yes ◯ No

Antihypertensive medication, specify

☐ Labetalol
☐ Nifedipine
☐ Hydralazine
☐ Other

Labetalol - prescribed daily dose

____
(mg/day)

Nifedipine - prescribed daily dose

____
(mg/day)

Hydralazine - prescribed daily dose

____
(mg/day)

Other hypertensive medication (include daily dose)

____

Comments form 8

Form 9 - Neonatal Discharge

Discharge neonate	☐ Neonatal/infant death before discharge
Date of neonatal/infant death	______________________
Age at time of death	______________________ (days)
Cause of death	______________________
Date of final discharge from hospital (when neonate is discharged from all care, including home care)	______________________ (YYYY-MM-DD)
Bronchopulmonary dysplasia	◯ Yes ◯ No
NICU admission	◯ Yes ◯ No
Neonatal care	◯ Yes ◯ No
Date for discharge from Neonatal care (including home care)	______________________
Date and time (12:00) discharge from Neonatal care	______________________
Nr of hours neonatal care From delivery until discharge neonatal care (12:00 on day of discharge)	______________________
Discharged home on oxygen treatment	◯ Yes ◯ No
Days on oxygen therapy	______________________
Days ventilated	______________________ ((Intubated and ventilated))
Days on nasal prongs	______________________ ((any % of oxygen))
Days on CPAP	______________________
Total days on any respiratory support	______________________
Comments form 9 Neonatal discharge	

PHASE-20

Instruktion till dig som ska använda PHASE-20

Denna skattningsskala är till för att uppmärksamma om du upplever besvär som möjligen kan bero på din läkemedelsbehandling. Dina svar kan vara av stort värde för att din läkare ska få en så bra helhetsbild som möjligt.

Läs ett besvär i taget och kryssa i den ruta som bäst överensstämmer med hur du upplevt eventuella besvär sedan förra besöket. Skriv gärna något i rutan för kommentar om du vill berätta något särskilt om ett visst besvär.

Ibland efterfrågas flera besvär, till exempel yr/ostadig/faller lätt. Där kan du förtydliga om något är extra framträdande genom att välja det besväret under "2. Ange de besvär som stämmer bäst", eller markera att du inte besväras av det genom att välja det besväret under "3. Ange de besvär som inte stämmer".

Markera rutan vid det alternativ som du bedömer bäst motsvarar besvärsnivån sedan förra besöket (1). Lämna förtydligande kommentarer i marginalen vid behov (4). Ange de besvär som stämmer bäst(2) och ange de besvär som inte stämmer (3).

1. Besvärsnivån sedan förra besöket 2. Ange de besvär som stämmer bäst 3. Ange de besvär som inte stämmer 4. Kommentar

1. Yr/ostadig/faller lätt ______ ______ ______ ______ ______ ______
2. Trött/dåsig/orkeslös ______ ______ ______ ______ ______ ______
3. Sover dåligt/mardrömmar ______ ______ ______ ______ ______ ______
4. Ont i magen/ont i bröstet ______ ______ ______ ______ ______ ______
5. Huvudvärk ______ ______
6. Nedstämd ______ ______
7. Orolig/ångestfylld ______ ______ ______ ______ ______ ______
8. Lättirriterad ______ ______
9. Glömsk ______ ______
10. Dålig aptit ______ ______
11. Muntorr ______ ______
12. Illamående/kräks ______ ______ ______ ______ ______ ______
13. Diarré ______ ______
14. Förstoppning ______ ______
15. Hjärtklappning ______ ______
16. Svullna ben/anklar ______ ______ ______ ______ ______ ______
17. Andfådd ______ ______
18. Täta trängningar/ urinläckage ______ ______ ______ ______ ______ ______
19. Klåda/utslag ______ ______ ______ ______ ______ ______
20. Annat, till exempel smärta (ange vad) ______

______

______

PHASE-20 - PHArmacotherapeutical Symptom Evaluation, 20 frågor

Syftet är att identifiera symtom som kan ha samband med läkemedelsbehandling, till exempel biverkningar, interaktionseffekter eller underbehandling. Skalan är framtagen i samarbete mellan Landstinget i Uppsala län och Regionförbundet Uppsala län.

Övriga kommentarer:

Behandlingsdagbok Mycap Check

Check the entries in the MyCap diary from [screening_randomis_arm_1][firstdose] - [ldate] (date of first dose - day before this visit) , by clicking on this link to PI4 MyCap

Check the entries in the MyCap diary from [screening_randomis_arm_1][firstdose] - [ldate] (date of first dose - day before this visit) , by clicking on this link to PI4 MyCap

Check the entries in the MyCap diary from [screening_randomis_arm_1][firstdose] - [ldate] (date of first dose - day before this visit) , by clicking on this link to PI4 MyCap

Check the entries in the MyCap diary from [date][previous-instance] - [ldate] (day of the previous visit - day before this visit), by clicking on this link to PI4 MyCap

Check the entries in the MyCap diary from [date][previous-instance] - [ldate] (day of the previous visit - day before this visit), by clicking on this link to PI4 MyCap

Check the entries in the MyCap diary from [date][previous-instance] - [ldate] (day of the previous visit - day before this visit), by clicking on this link to PI4 MyCap

Metformin was permanently stopped previously

If any Metformin was taken between [vdate][previous-instance] and [ldate], please correct the previous form!

Date before this visit ________________________________

After review, please complete below:

Was metformin stopped/paused during this period (= 0 tablets taken on one or more days) ◯ Yes ◯ No

Specify why patient stopped taking metformin ________________________________

Any changes to metformin dosing after this visit?
- ◯ No
- ◯ Yes
- ◯ Dose changed
- ◯ Dosing paused
- ◯ Dosing permanently stopped

Reason metformin [met_change_app]
- ◯ Patient decision
- ◯ Investigator instruction

Specify why patient decided to stop metformin or change dose after this visit ________________________________

Reason metformin permanently stopped by investigator	◯ Lactacidosis<br>◯ Unacceptable adverse events/adverse reactions<br>◯ The subject does not follow procedures in the study protocol<br>◯ Renal disease or renal dysfunction (suggested by serum creatinine levels ≥125 umol/L or abnormal creatinine clearance) which may also result from conditions such as cardiovascular collapse (shock), acute myocardial infarction, septicaemia and preeclampsia causing renal failure<br>◯ Acute or chronic metabolic acidosis<br>◯ Acute conditions with the potential to alter renal function, e.g., dehydration, severe infection, shock, intravascular administration of iodinated contrast media<br>◯ Acute or chronic disease which may cause tissue hypoxia such as cardiac or respiratory failure, pancreatitis, recent myocardial infarction, shock<br>◯ Hepatic insufficiency, acute alcohol intoxication, alcoholism (acute or chronic)<br>◯ Gastroenteritis<br>◯ Surgery under general anesthesia<br>◯ High fever<br>◯ Need of contrast radiography<br>◯ Other: ______
Reason metformin paused or dose changed by investigator	◯ Gastroenteritis<br>◯ Surgery under general anesthesia<br>◯ High fever<br>◯ Need of contrast radiography<br>◯ Other: ______
If changed dose, new daily dose if dose should be increased in steps, describe below and provide the target dose for the next visit here	______________________________<br>(tablets/day)
Description of dose change (if done in steps)	
Comments Behandlingsdagbok Mycap Check	

Behandlingsdagbok Paper

Record from the patient's diary from the day of the first dose - day before this visit

Record from the patient's diary from the day of the previous visit - day before this visit

This visit was the last visit before delivery on [delivery_arm_1][delivery_date].

Tick this box if the delivery took place after this recurrent visit. All days until and including the delivery date will occur in the schedule below (if completed on Form 7 - Delivery details).

☐ Yes

Metformin was permanently stopped previously

If any Metformin was taken between [date1] and [vdate], please correct the previous form!

I=Illamående K=Kräkningar D=Diarré H=Huvudvärk A=Annat

Morgon Lunch Kväll Besvär Annat besvär/kommentar
[date1] ______ ______ ______ ______ ______

[date2] ______ ______ ______ ______ ______

[date3] ______ ______ ______ ______ ______

[date4] ______ ______ ______ ______ ______

[date5] ______ ______ ______ ______ ______

[date6] ______ ______ ______ ______ ______

[date7] ______ ______ ______ ______ ______

Date - date of previous visit ________________________________

Date ________________________________

Date ________________________________

Date ________________________________

Date ________________________________

Date ________________________________

Date	______________________________
After completion of diary information, complete below section	
Specify why patient stopped taking metformin	______________________________
Any changes to metformin dosing after this visit?	◯ No<br>◯ Yes<br>◯ Dose changed<br>◯ Dosing paused<br>◯ Dosing permanently stopped
Reason Metformin [met_stop]	◯ Patient decision<br>◯ Investigator instruction
Specify why patient decided to stop metformin or change dose after this visit	______________________________
Reason metformin permanently stopped by investigator	◯ Lactacidosis<br>◯ Unacceptable adverse events/adverse reactions<br>◯ The subject does not follow procedures in the study protocol<br>◯ Renal disease or renal dysfunction (suggested by serum creatinine levels ≥125 umol/L or abnormal creatinine clearance) which may also result from conditions such as cardiovascular collapse (shock), acute myocardial infarction, septicaemia and preeclampsia causing renal failure<br>◯ Acute or chronic metabolic acidosis<br>◯ Acute conditions with the potential to alter renal function, e.g., dehydration, severe infection, shock, intravascular administration of iodinated contrast media<br>◯ Acute or chronic disease which may cause tissue hypoxia such as cardiac or respiratory failure, pancreatitis, recent myocardial infarction, shock<br>◯ Hepatic insufficiency, acute alcohol intoxication, alcoholism (acute or chronic)<br>◯ Gastroenteritis<br>◯ Surgery under general anesthesia<br>◯ High fever<br>◯ Need of contrast radiography<br>◯ Other: ______
Reason metformin paused or dose changed by investigator	◯ Gastroenteritis<br>◯ Surgery under general anesthesia<br>◯ High fever<br>◯ Need of contrast radiography<br>◯ Other: ______
If changed dose, new daily dose<br><br>- if dose should be increased in steps, describe below and provide the target dose for the next visit here	______________________________<br>(tablets/day)
Description of dose change (if done in steps)	

Comments Behandlingsdagbok Paper

Total dose for this page

____

Number of tablets taken in total until this page

____

Form 10 - Two Months Postpartum Outcome

Date of visit

____
(YYYY-MM-DD)

Neonate follow-up

Neonatal follow-up may not be collected since caregiver(s) did not consent (consent 2)

Neonatal death (death from birth until 28 days of age) ◯ No ◯ Yes

Infant death (death after 28 days of age) ◯ No ◯ Yes

Date of neonatal/infant death

____
(YYYY-MM-DD)

Age at time of death

____
(days)

Infant death occurred after [w_age_death_days] days. Please change from Neonatal death to Infant death above

Neonatal death occurred after [w_age_death_days] days. Please change from Infant death to Neonatal death above

Below questions should also be completed in case of neonatal or infant death

Grade III or IV intraventricular haemorrhage, diagnosed on ultrasound ◯ No ◯ Yes

Necrotising enterocolitis, defined as necrotizing enterocolitis requiring surgery ◯ No ◯ Yes

Invasive ventilation, intubation and mechanical ventilation or non-invasive ventilation; continuous positive airway pressure (CPAP) support or high flow nasal oxygen ◯ No ◯ Yes

Grade III or IV hyaline membrane disaease ◯ No ◯ Yes

Surfactant use ◯ No ◯ Yes

Retinopathy of prematurity, defined as requiring laser therapy or bevacizumab treatment ◯ No ◯ Yes

Neonatal sepsis, defined as a confirmed laboratory bloodstream infection treated with a course of antibiotics ◯ No ◯ Yes

Maternal follow-up Blood pressure is taken as below:

- If < 140/90: take another blood pressure immediately
- If >140/90: wait 15 min and take another blood pressure
- Enter both blood pressures Matning 1

Maternal systolic blood pressure ______________________________

Maternal diastolic blood pressure ______________________________

Mätning 2

Maternal systolic blood pressure ______________________________

Maternal diastolic blood pressure ______________________________

Is the mother on blood pressure treatment ◯ Yes ◯ No

Blood pressure treatment at discharge:

Treatment Daily dose   Still taken? Stop date
Labetalol [discharge_arm_1][labetalol_dose] mg ______ ______
Nifedipine [discharge_arm_1][nifedipine_dose] mg ______ ______
Hydralazine [discharge_arm_1][hydralazine_dose] mg ______ ______
Other: [discharge_arm_1][med_hypertens_disch_other] ______ ______

New blood pressure treatment since discharge
- ☐ No
- ☐ Labetalol
- ☐ Nifedipine
- ☐ Hydralazine
- ☐ Enalapril
- ☐ Metoprolol
- ☐ Calcium channel blockers
- ☐ Other

(tick all that apply)

Labetalol - prescribed daily dose ______________________________ (mg/day)

Nifedipine - prescribed daily dose ______________________________ (mg/day)

Hydralazine - prescribed daily dose ______________________________ (mg/day)

Enalapril - prescribed daily dose

__________________ (mg/day)

Metoprolol - prescribed daily dose

__________________ (mg/day)

Calcium channel blockers - prescribed daily dose

__________________ (mg/day)

Other blood pressure treatment (include daily dose)

____

Final hypertensive diagnosis

- ◯ Preeclampsia
- ◯ Chronic hypertension with superimposed preeclampsia

Why did you decide to participate in the study?

Instructions:

- Ask the question "Varför valde du att delta i studien"
- Enter the reasons in the order they come up

Comments form 10 Two Months Postpartum Outcome

Form 11 Two Years Postpartum Outcome

Date

____
(YYYY-MM-DD)

Maternal follow-up Blood pressure is taken as below:

- If < 140/90: take another blood pressure immediately

- If >140/90: wait 15 min and take another blood pressure

- Enter both blood pressures

1st blood pressure ______ / ______
2nd blood pressure ______ / ______

Medications - tick all that are currently taken

- ☐ ACE inhibitor
- ☐ AT-2 inhibitor
- ☐ Diuretics
- ☐ Betablocker
- ☐ Aspirin
- ☐ Insulin
- ☐ Oral diabetes medication
- ☐ Lipid lowering medication
- ☐ Levothyroxine
- ☐ Antidepressants
- ☐ Sleep medicine
- ☐ Calcium antagonist
- ☐ None of the above

Postpartum hypertension?

◯ Yes

Approximately how many weeks after delivery did you receive antihypertensive treatment?

____
(weeks)

Antihypertensive treatment?

◯ Ongoing

Breastfeeding

Answers from questionnaire at 2-3 months follow-up:

- Never breastfed

- Stopped breastfeeding exclusively after [23_months_followup_arm_1][bf_ft_stop] weeks

- Stopped breastfeeding partially at [23_months_followup_arm_1][bf_pt_stop] weeks

- Partially breastfeeding at 2-3 months follow-up

- Exclusively breastfeeding at 2-3 months follow-up

How long did you exclusively breastfeed for?	◯ 2 months<br>◯ 3 months<br>◯ 4 months<br>◯ 5 months<br>◯ 6 months<br>◯ 7 months<br>◯ 8 months<br>◯ 9 months<br>◯ 10 months<br>◯ 11 months<br>◯ 12 months<br>◯ 13 months<br>◯ 14 months<br>◯ 15 months<br>◯ 16 months<br>◯ 17 months<br>◯ 18 months<br>◯ Still breastfeeding
How long did you partially breastfeed for?	◯ 2 months<br>◯ 3 months<br>◯ 4 months<br>◯ 5 months<br>◯ 6 months<br>◯ 7 months<br>◯ 8 months<br>◯ 9 months<br>◯ 10 months<br>◯ 11 months<br>◯ 12 months<br>◯ 13 months<br>◯ 14 months<br>◯ 15 months<br>◯ 16 months<br>◯ 17 months<br>◯ 18 months<br>◯ Still breastfeeding
Have you regained your period?	◯ Yes ◯ No
Do you use contraception?	◯ Yes ◯ No
What contraception?	◯ Combined oral contraception<br>◯ Gestagen only oral contraception<br>◯ Gestagen implant<br>◯ Gestagen intrauterine device<br>◯ No hormonal contraception<br>◯ Other
Other contraception (details)	______________________________

After delivery, have you been seen in healthcare for any of the following diseases?	☐ Migraine<br>☐ High blood pressure<br>☐ Visual disturbances<br>☐ Diabetes<br>☐ Thyroid function disorder<br>☐ Allergies<br>☐ Irritable colon<br>☐ Alcohol or drug addiction<br>☐ Depression<br>☐ Anxiety<br>☐ Pain-related disorders<br>☐ None of the above

Pregnancy

Are you currently pregnant?	◯ Yes ◯ No
Was there a pregnancy after discharge and before follow-up (excluduing ongoing pregnancy)	◯ Yes ◯ No
Number of pregnancies	◯ 1 ◯ 2 ◯ 3 ◯ 4<br>◯ > 4

Pregnancy 1

Type of pregancy	◯ Pregnancy terminating before 21+6 GA<br>◯ Pregnancy beyond 21+6 GA
Outcome (pregnancy terminating before 21+6 GA)	◯ Early miscarriage (12w or less)<br>◯ Late miscarriage (13, 23w)<br>◯ Ectopic pregnancy<br>◯ Induced abortion<br>◯ Molar pregnancy<br>◯ Unknown<br>◯ No information
Outcome (pregnancy beyond 21+6 GA)	◯ Liveborn<br>◯ Stillbirth<br>◯ Neonatal death<br>◯ Unknown<br>◯ No information
GA (w)	______________________________
Gestational Hypertension	◯ Yes ◯ No
Preeclampsia	◯ Yes ◯ No
Eclampsia	◯ Yes ◯ No
HELLP	◯ Yes ◯ No
Placental Abruption	◯ Yes ◯ No
SGA and/or FGR	◯ Yes ◯ No

Delivery details	◯ Caesarean section<br>◯ Induced preterm delivery<br>◯ Spontaneous preterm delivery
Treatment details	______________________
Any other severe complication during pregnancy	◯ Yes ◯ No
Other severe complication during pregnancy - specify	______________________

Pregnancy 2

Type of pregancy	◯ Pregnancy terminating before 21+6 GA<br>◯ Pregnancy beyond 21+6 GA
Outcome (pregnancy terminating before 21+6 GA)	◯ Early miscarriage (12w or less)<br>◯ Late miscarriage (13, 23w)<br>◯ Ectopic pregnancy<br>◯ Induced abortion<br>◯ Molar pregnancy<br>◯ Unknown<br>◯ No information
Outcome (pregnancy beyond 21+6 GA)	◯ Liveborn<br>◯ Stillbirth<br>◯ Neonatal death<br>◯ Unknown<br>◯ No information
GA (w)	______________________
Gestational Hypertension	◯ Yes ◯ No
Preeclampsia	◯ Yes ◯ No
Eclampsia	◯ Yes ◯ No
HELLP	◯ Yes ◯ No
Placental Abruption	◯ Yes ◯ No
SGA and/or FGR	◯ Yes ◯ No
Delivery details	◯ Caesarean section<br>◯ Induced preterm delivery<br>◯ Spontaneous preterm delivery
Treatment details	______________________

Any other severe complication during pregnancy	◯ Yes ◯ No
Other severe complication during pregnancy - specify	__________________________________

Pregnancy 3

Type of pregancy	◯ Pregnancy terminating before 21+6 GA<br>◯ Pregnancy beyond 21+6 GA
Outcome (pregnancy terminating before 21+6 GA)	◯ Early miscarriage (12w or less)<br>◯ Late miscarriage (13, 23w)<br>◯ Ectopic pregnancy<br>◯ Induced abortion<br>◯ Molar pregnancy<br>◯ Unknown<br>◯ No information
Outcome (pregnancy beyond 21+6 GA)	◯ Liveborn<br>◯ Stillbirth<br>◯ Neonatal death<br>◯ Unknown<br>◯ No information
GA (w)	____________________________
Gestational Hypertension	◯ Yes ◯ No
Preeclampsia	◯ Yes ◯ No
Eclampsia	◯ Yes ◯ No
HELLP	◯ Yes ◯ No
Placental Abruption	◯ Yes ◯ No
SGA and/or FGR	◯ Yes ◯ No
Delivery details	◯ Caesarean section<br>◯ Induced preterm delivery<br>◯ Spontaneous preterm delivery
Treatment details	__________________________________
Any other severe complication during pregnancy	◯ Yes ◯ No
Other severe complication during pregnancy - specify	__________________________________

Pregnancy 4

Type of pregancy	◯ Pregnancy terminating before 21+6 GA<br>◯ Pregnancy beyond 21+6 GA
Outcome (pregnancy terminating before 21+6 GA)	◯ Early miscarriage (12w or less)<br>◯ Late miscarriage (13, 23w)<br>◯ Ectopic pregnancy<br>◯ Induced abortion<br>◯ Molar pregnancy<br>◯ Unknown<br>◯ No information
Outcome (pregnancy beyond 21+6 GA)	◯ Liveborn<br>◯ Stillbirth<br>◯ Neonatal death<br>◯ Unknown<br>◯ No information
GA (w)	__________________________________
Gestational Hypertension	◯ Yes ◯ No
Preeclampsia	◯ Yes ◯ No
Eclampsia	◯ Yes ◯ No
HELLP	◯ Yes ◯ No
Placental Abruption	◯ Yes ◯ No
SGA and/or FGR	◯ Yes ◯ No
Delivery details	◯ Caesarean section<br>◯ Induced preterm delivery<br>◯ Spontaneous preterm delivery
Treatment details	__________________________________
Any other severe complication during pregnancy	◯ Yes ◯ No
Other severe complication during pregnancy - specify	__________________________________

Pregnancy 5 onwards

Provide details of the other pregnancy/pregnancies:

Other medical diagnosis

Any Medical Conditions to record that were diagnosed after discharge? ◯ Yes ◯ No

Specify medical conditions diagnosed after discharge

Adverse Events

Initial report or follow-up	◯ Initial ◯ Follow-up
AE description	________________________________
AE description copy - SAEs to be followed up	________________________________
SAE to follow-up	◯ [sae_term_fu][1]<br>◯ [sae_term_fu][2]<br>◯ [sae_term_fu][3]<br>◯ [sae_term_fu][4]<br>◯ [sae_term_fu][5]<br>◯ [sae_term_fu][6]<br>◯ [sae_term_fu][7]<br>◯ [sae_term_fu][8]<br>◯ [sae_term_fu][9]<br>◯ [sae_term_fu][10]
Date of follow-up	________________________________
AE start date	________________________________<br>(yyyy-mm-dd)
AE start time	________________________________
AE intensity	◯ Mild<br>◯ Moderate<br>◯ Severe
SAE maximum intensity	◯ Mild<br>◯ Moderate<br>◯ Severe
AE outcome	◯ Resolved<br>◯ Ongoing<br>◯ Unresolved<br>◯ Fatal<br>(If resolved, provide end date)
AE end date	________________________________<br>(yyyy-mm-dd)
Causal relationship to study drug/treatment	◯ Likely<br>◯ Possibly<br>◯ Unlikely<br>◯ Not related<br>◯ Unknown/unclassifiable
AE serious	◯ Yes ◯ No

Date investigator aware of SAE	______________________________
Date AE became serious	______________________________
Serious criteria	☐ Leads to death<br>☐ Is life-threatening<br>☐ Requires inpatient hospitalization or prolongation of existing hospitalization not related to preeclampsia and complications of preeclampsia<br>☐ Results in persistent or significant disability or incapacity<br>☐ Jaundice with bilirubin > 50 ul/L<br>☐ Results in congenital anomaly/malformation<br>☐ Other*<br>(select all criteria that apply)
* Other: An important medical event that may not be immediately life-threatening or result in death or hospitalisation, that may jeopardise the participant or that may require intervention to prevent serious outcomes (see ICH E2A and E19) should generally be considered as serious."	
Date of death	______________________________
Time of death	______________________________<br>(provide if available)
Hospitalization date (SAE related hospitalization)	______________________________
Discharge date (SAE related hospitalization)	______________________________
SAE description:<br>(Describe the SAE: e.g. symptoms, treatment)	
Describe how the SAE developed until resolution	
Is the event an Adverse event of Special Interest (AESI) (i.e. lactacidosis)?	◯ Yes ◯ No
For unresolved Adverse events, please update the information above when the adverse event is resolved or changed.	

Overdose

**Overdose form**<br><br>**Definition of overdose: More than the maximum defined dose in the protocol at any dosing event (i.e. > 2 tablets)**	
Date overdose	______________________
Who took the overdose?	◯ Participant ◯ Other person
Specify other person (e.g. relation to participant)	______________________
Dose - number of tablets à 500 mg	______________________ (tablets)
Intentional overdose?	◯ Yes ◯ No
Description of event	______________________
Side effects	______________________
Was treatment unblinded?	◯ Yes ◯ No
If yes, did participant receive metformin?	◯ Yes ◯ No

Discontinuation Completion

Was the study participation discontinued prematurely (i.e. withdrawal of consent)

If Metformin was discontinued, this should be reported on the Diary check form ("Behandlingsdagbok")

◯ Yes ◯ No

Date of discontinuation

____

Reason premature study discontinuation

◯ Withdrawal of consent
◯ Investigator/sponsor decision

Investigator/sponsor decision - stop criteria/reason

◯ Lactacidosis
◯ Unacceptable adverse events/adverse reactions
◯ The subject does not follow procedures in the study protocol
◯ Renal disease or renal dysfunction (suggested by serum creatinine levels ≥125 umol/L or abnormal creatinine clearance) which may also result from conditions such as cardiovascular collapse (shock), acute myocardial infarction, septicaemia and preeclampsia causing renal failure
◯ Acute or chronic metabolic acidosis
◯ Acute conditions with the potential to alter renal function, e.g., dehydration, severe infection, shock, intravascular administration of iodinated contrast media
◯ Acute or chronic disease which may cause tissue hypoxia such as cardiac or respiratory failure, pancreatitis, recent myocardial infarction, shock
◯ Hepatic insufficiency, acute alcohol intoxication, alcoholism (acute or chronic)

Date consent withdrawn

____

Reason consent withdrawn

____

Comment consent withdrawal

____

Comments Discontinuation Completion

IP compliance

Was additional IP ordered and received?	◯ Yes ◯ No
Nr of bottles (168 tablets per bottle)	◯ 1 ◯ 2
Treatment pack number	◯ [screening_randomis_arm_1][rx_pack] (same as previous pack number)<br>◯ Other pack number: ______
Total number of tablets taken (paper diary)	______________________________
IP provided	______________________________
Nr of tablets left calculated : provided - nr of tablets taken	______________________________

Number of tablets taken MyCap* -

copy from MyCap database (link)

______

Number of tablets taken MyCap* -

copy from MyCap database (link)

______

Number of tablets taken MyCap* -

copy from MyCap database (link)

______

Number of tablets taken paper

[total_tablets_final_paper]

Number of tablets provided [tablets_provided_total]
Number of tablets left - expected [total_tablets_left_calc]

Number of tablets left - counted ______

Provide a (possible) explanation why less tablets were returned ([total_tablets_left_count]) than expected ([total_tablets_left_calc])

More tablets are returned than calculated (less tablets were taken than reported in the diary) - Check the diary entries - Report to local PI - Provide a (possible) reason

Note to file

Deviation (avvikelse)

Date reported
