# Supplementary material for: Can metformin prolong pregnancy in preterm pre-eclampsia? Study protocol for a Nordic multicentre randomised placebo-controlled trial (PI4)
Source: BMJ Open. 2026 Oct 13;16(10):e120993. doi: 10.1136/bmjopen-2026-120993 (PMC13663727; doi:10.1136/bmjopen-2026-120993)
Supplement: Supplementary file 4 [file bmjopen-2026-120993.reviewer_comments.pdf]

PEER REVIEW HISTORY

BMJ Open publishes all reviews undertaken for accepted manuscripts. Reviewers are asked to complete a checklist review form and are provided with free text boxes to elaborate on their assessment. These free text comments are reproduced below.

ARTICLE DETAILS

Title (Provisional)

Can metformin prolong pregnancy in preterm pre-eclampsia? Study Protocol for a Nordic multicentre randomised placebo-controlled trial (PI4)

Authors

Gudmundsson, Pia; Cluver, Catherine Anne; Sandström, Anna; Lindström, Linda; Timpka, Simon; Hesselman, Susanne; Lilliecreutz, Caroline; Brismar Wendel, Sophia; Von Wowern, Emma; Linden, Karolina; Andersson, Ola; Gyllensten, Hanna; Alkmark, Mårten; Norrman, Emma; Wesslén, Erica; Mantel, Angla; Blomberg, Marie; Aronzon, Hugo; Laivuori, Hannele; Villa, Pia; Michelsen, Trond Melbye; Sørbye, Ingvil Krarup; Haavaldsen, Camilla; Vinnars, Marie-Therese; Tiblad, Eleonor; Raab, Nadine; Toivonen, Elli; Hellström, Ann; Sävman, Karin; Ohlsson, Hanna; Berver, Stina; Ottosson, Emelie; Bejlum, Carina; Kullinger, Merit; Stenfeldt, Karin; Walker, Susan P; Tong, Stephen; Imberg, Henrik; Bergman, Lina

---

VERSION 1 - REVIEW

**Reviewer**	**1**
**Name**	**Amorim, Melania**
**Affiliation**	**Universidade Federal de Campina Grande, Obstetrics and Gynaecology**
**Date**	**10-May-2026**
**COI**	**None**

Dear Authors,

Thank you for the opportunity to review this protocol for the Pre-eclampsia Intervention 4 (PI4) trial — a phase III, double-blind, multicentre, randomised placebo-controlled trial of metformin extended-release for prolongation of gestation in preterm pre-eclampsia, conducted across fourteen main and four satellite sites in Sweden, Norway, and Finland.

This is, on every methodological dimension, a strong protocol. The trial is the high-resource Nordic counterpart of the South African PI2 (Cluver et al., BMJ 2021) and PI3 trials and of the Dutch PI-NL trial, and is part of a coordinated programme designed for individual-participant-data meta-analysis. The design is sound: 1:1 randomisation with permuted

blocks of varying size, stratified by trial site and gestational-age group at inclusion; centrally generated allocation list incorporated into the REDCap eCRF; double-blinding maintained through identical placebo packaging; sealed envelopes for emergency unblinding stored with the trial medication; intention-to-treat as primary analysis population; sophisticated time-to-event modelling with explicit handling of right-censoring at 37+0 weeks and interval-censoring for IUFD; Fine-Gray competing-risks model for length of neonatal stay; hierarchical testing procedure for type I error control. Regulatory governance is rigorous (EU Clinical Trials Regulation via CTIS, Swedish Medical Products Agency, Swedish Ethical Review Authority, with national approvals in Norway and Finland). An independent DSMB with appropriate composition (obstetrician, paediatrician, statistician) reviews safety annually. Patient and public involvement, mediated by the Preeklampsiföreningen, is integrated into design and dissemination. The cost-effectiveness evaluation, biobanking strategy with biomarker substudies, and two-year follow-up of the child's cognitive development through validated instruments (ASQ-3, M-CHAT-R, Bayley-4) substantially extend the value of the trial beyond its primary endpoint.

My comments below are clarifications and refinements rather than substantive concerns about the trial itself. The protocol is publishable, in my assessment, after these points are addressed.

Major comments

1. Statistical methods in the main manuscript are too compressed relative to the sophistication of the protocol. The Statistical methods section in the manuscript (approximately two paragraphs) describes the analytical strategy at a high level — "appropriate parametric or semi-parametric models", "censoring and competing events will be handled where relevant" — without conveying that the primary analysis is a log-normal mixed-effects censored regression model with right-censoring at 37+0 weeks planned induction and interval-censoring for intrauterine fetal demise; that length of neonatal stay is analysed by Fine-Gray subdistribution hazard model with stillbirth and neonatal death as competing events; that neonatal birthweight is analysed by log-normal mixed-effects model; or that trial site enters as random effect in mixed models and fixed effect in time-to-event analyses. These details are in Online Supplemental Appendix 1 (Protocol v7.1, sections 10.1–10.2), but readers of the published protocol should not have to consult the supplement to understand the analytical strategy. Suggest expanding the Statistical methods section in the main manuscript to summarise these specific approaches, while still pointing to the protocol and forthcoming SAP for full detail.

2. The hierarchical testing order is not specified in the manuscript. The manuscript states that "the full significance level (α=0.05) will be sequentially allocated to the secondary outcomes, in a predefined order of testing", without specifying what that order is. The protocol clarifies the sequence (length of neonatal stay → birthweight). Specifying the testing order in the manuscript is a transparency requirement — it pre-empts any later

question about the ordering being data-driven and is the standard expectation under the SPIRIT-Outcomes 2022 recommendations (item 20a.1).

3. The censoring strategy for the primary outcome should be made explicit in the manuscript. The handling of right-censoring at 37+0 weeks (when planned induction occurs without other maternal or fetal indication) and interval-censoring of intrauterine fetal demise is critical for the interpretation of "prolongation of gestation defined as time from randomisation to birth", because both conventions have potential consequences for the estimated treatment effect. A 1–2 sentence description in the Outcomes or Statistical methods section of the manuscript would suffice.

4. Reference 32 (Zhang Y et al. 2021) is published in Oxidative Medicine and Cellular Longevity, a Hindawi journal. Hindawi has, since 2022, retracted in excess of 1,700 papers across its portfolio for systematic manipulation of the publication and peer-review process; Oxidative Medicine and Cellular Longevity has been particularly affected, with hundreds of retractions in 2023–2024. The article cited here has not, as of this writing, been retracted, and its mechanistic content is thematically appropriate to the assertion it sustains in the manuscript discussion. However, citation of journals with documented integrity concerns is increasingly discouraged in the methodological literature. Consider replacing the reference with a non-Hindawi source covering a similar mechanistic point — for example, Han et al. 2025 in BMC Pregnancy and Childbirth on insulin-resistance-induced trophoblast pyroptosis and its protection by metformin (doi:10.1186/s12884-025-07419-0) covers a closely related mechanism in a non-Hindawi venue.

5. The manuscript does not contain a declaration of use or non-use of artificial intelligence in its preparation. BMJ guidelines now require this declaration explicitly. Please add a brief statement in an appropriate section (Acknowledgments or Methods).

6. The conflict-of-interest disclosure could be more specific. The current statement — "LB, CC, SW, ST and HI provide consulting services to companies developing novel therapeutics for pre-eclampsia" — does not allow readers to assess the nature of the conflicts in relation to the trial drug or its potential comparators. Consider naming the companies involved, in line with ICMJE recommendations for transparent disclosure.

Minor comments

7. Viability assessment between 22+0 and 23+6 weeks. The eCRF (Online Supplemental Appendix 3, Form 1) indicates that for participants between 22+0 and 23+6 weeks at inclusion, the sponsor must be contacted for discussion regarding viability before randomisation. This is good practice, but is not described in the main manuscript. Brief mention in the Inclusion criteria or Participant enrolment section would be helpful, particularly for international readers operating in jurisdictions where viability thresholds may differ.

8. Sample-size narrative could be sharpened. The Strengths bullet "a clinically significant 50% increase in prolongation of gestation from randomisation to birth of 3.5 days" combines a relative effect (50% increase) and an absolute effect (3.5 days) in a way that is initially confusing. Suggest: "an absolute increase of 3.5 days from a median prolongation of 7 days, corresponding to a 50% relative increase". It is also worth noting in the Discussion that PI2 estimated a geometric mean ratio of 1.39 (95% CI 0.99–1.95) in the prolongation of gestation, slightly below the GMR = 1.50 that PI4 is sized to detect; if the true effect is closer to the PI2 point estimate, PI4 may also yield a borderline result, and the planned IPD meta-analysis with PI3 and PI-NL is therefore an important component of the evidentiary strategy. The current Strengths-and-limitations panel mentions this in general terms; making the link between trial size, PI2's effect estimate, and the IPD meta-analysis more explicit would benefit readers.

9. Reference 24 (ACOG Practice Bulletin 180 on Gestational Diabetes Mellitus, Obstet Gynecol 2017) appears in the reference list but I could not locate a clear in-text citation in the manuscript body. Please verify whether the reference is cited; if not, remove from the list.

10. References 20–22 (Nordic clinical guidelines on hypertensive disorders of pregnancy, in Swedish, Finnish, and Norwegian) are appropriate for justifying the standardised care across sites, but are local-language sources potentially inaccessible to international readers. Consider providing English-language titles or brief descriptive annotations of what each guideline contains. Reference 25 (UN Sustainable Development Goals website homepage) could be made more specific by pointing to the SDG 3.1 indicator on maternal mortality reduction or to a relevant UN technical report.

11. Typographical and editorial points:

— "concentrations similar to those observed in to non-pregnant individuals" (Intervention section) — read "to those observed in non-pregnant individuals" (delete "in to").

— "Mars 11 2024" in the Ethics section — should read "March 11, 2024".

— Some inconsistencies in reference formatting (extra spaces, hyphenation around author names) reflect Endnote artefacts and merit a final pass before publication.

12. PI2 / PI3 / PI-NL relationship and the planned IPD meta-analysis. The Discussion mentions the parallel trials and the planned IPD meta-analysis, but the methodological framing of "we will participate in a prospectively planned IPD meta-analysis with harmonised outcomes" deserves earlier prominence — perhaps in the Methods (after Sample size calculation) or in a brief paragraph in the Introduction. The fact that four trials are running in parallel with harmonised primary and secondary outcomes is a significant strength and is currently dispersed across the abstract Strengths bullet and the Discussion.

13. Consent to follow-up of the child (consent 2). The eCRF and protocol indicate that two consents are obtained — consent 1 from the participant before birth, and consent 2 from

caregivers post-birth for child follow-up — and that biological samples and follow-up data must be destroyed if consent 2 is not granted. This is well-handled operationally; brief mention of the dual-consent structure in the Ethics section of the manuscript would clarify the procedure for readers.

14. SPIRIT item 18a.1 — Responsiveness of study instruments. The submitted SPIRIT-Outcomes 2022 checklist marks this item as "-". The trial uses validated instruments (ASQ-3, M-CHAT-R, Bayley-4, plus PHASE-20 in the eCRF for medication-related symptoms). A brief sentence summarising what is known about responsiveness of these instruments in similar populations would address the checklist item.

15. SPIRIT item 26b — Additional consent provisions for ancillary studies. Listed as "-" in the submitted checklist, but the trial does have a separate consent (consent 2) for child follow-up and biological-sample use, and the eCRF makes provisions for destruction of samples if consent 2 is withheld. Please complete this checklist item with the manuscript location.

16. SPIRIT item 31b — Authorship eligibility guidelines. Listed as "-". A brief mention of adherence to ICMJE recommendations would address this checklist item.

Closing remarks

This is a methodologically robust, well-organised, internationally relevant phase III trial protocol from a competent and experienced consortium. The PI4 trial fills an important gap in the metformin-for-preterm-pre-eclampsia evidence base by providing high-resource, multicentre, Nordic data that complement the existing PI2 trial and the ongoing PI3 (South Africa) and PI-NL (Netherlands) trials, with a prospectively planned IPD meta-analysis. The clinical question is important: an effective intervention to delay disease progression in preterm pre-eclampsia would represent a substantial advance in maternal-fetal medicine, with implications for guideline development across high- and low-resource settings.

My recommendation is Minor Revision. The points above are clarifications and refinements that, in my view, do not require structural changes to the trial or protocol but would strengthen the published manuscript. I would be glad to review a revised version.

Sincerely,

Melania Amorim, MD, PhD

**Reviewer**	**2**
**Name**	**Di Simone , Nicoletta**
**Affiliation**	**Humanitas University**
**Date**	**06-Jul-2026**
**COI**	**None**

This is a well-designed and clinically relevant study protocol addressing an important unmet need in the management of preterm pre-eclampsia. The multicentre, randomised, double-blind design is a major strength. Nevertheless, the manuscript would benefit from several revisions to strengthen its scientific rationale.

First, the biological basis for the intervention should be better developed. Although previous clinical evidence is cited, the manuscript does not describe or hypothesise the mechanisms through which metformin may prolong pregnancy or influence the pathophysiology of pre-eclampsia.

Second, the rationale for the selected dosage of extended-release metformin (1 g three times daily) should be clearly explained.

Finally, the authors should further justify the effect size assumed for the sample size calculation, discuss the clinical relevance of prolongation of gestation as the primary endpoint in terms of expected neonatal benefit, and briefly describe the planned approach to safety monitoring and management of missing data.

VERSION 1 - AUTHOR RESPONSE

REVIEWER 1

Reviewer: 1, Comment 1
Statistical methods in the main manuscript are too compressed relative to the sophistication of the protocol. The Statistical methods section in the manuscript (approximately two paragraphs) describes the analytical strategy at a high level — "appropriate parametric or semi-parametric models", "censoring and competing events will be handled where relevant" — without conveying that the primary analysis is a log-normal mixed-effects censored regression model with right-censoring at 37+0 weeks planned induction and interval-censoring for intrauterine fetal demise; that length of neonatal stay is analysed by Fine-Gray subdistribution hazard model with stillbirth and neonatal death as competing events; that neonatal birthweight is analysed by log-normal mixed-effects model; or that trial site enters as random effect in mixed models and fixed effect in time-to-event analyses. These details are in Online Supplemental Appendix 1 (Protocol v7.1, sections 10.1–10.2), but readers of the published protocol should not have to consult the supplement to understand the analytical strategy. Suggest expanding the Statistical methods section in the main manuscript to summarise these specific approaches, while still pointing to the protocol and forthcoming SAP for full detail.
Thank you for this comment. We have revised the Statistical Methods section accordingly and expanded the description of the planned analytical strategy in the main manuscript, including the statistical models for the primary and secondary outcomes and the handling of censoring and competing events.
Page 17, Lines 5-8, Page 18, Lines 1-14
Statistical analyses will be conducted according to the intention-to-treat principle, with all randomised participants analysed according to their randomised treatment allocation. The primary outcome, prolongation of gestation, will be analysed using a log-normal mixed-effects censored regression model, and treatment effects will be presented as geometric

mean ratios with 95% confidence intervals. Planned induction at 37+0 weeks' gestation without another maternal or fetal indication for delivery will be treated as right-censoring. In cases of intrauterine fetal demise, the event time will be treated as interval-censored between the last assessment confirming fetal viability and the diagnosis of fetal demise. Neonatal length of stay will be analysed using a Fine-Gray subdistribution hazards model, with stillbirth and neonatal death treated as competing events, and treatment effects will be presented as subdistribution hazard ratios with 95% confidence intervals. Neonatal birthweight will be analysed using a log-normal mixed-effects model, and treatment effects will be presented as geometric mean ratios with 95% confidence intervals.
All primary and secondary efficacy analyses will be adjusted for the stratification variables (gestational age group at randomisation and trial site). Additional prognostic factors may be included as prespecified covariates in the Statistical Analysis Plan (SAP), based on findings from the observational GO PROVE study. Trial site will be included as a random effect in mixed-effects models and as a fixed effect in the Fine-Gray model. Safety analyses will be conducted on the safety population, defined as all randomised participants who received at least one dose of the trial medication.

Page 18, Lines 20-22
If the primary outcome is statistically significant, the full significance level (α=0.05) will be sequentially allocated to the secondary outcomes, in the following prespecified order: neonatal length of stay followed by neonatal birthweight.

Page 18 Lines 23-27 and Page 19, Lines 1-5
Statistical significance will be declared for each outcome with a significant result until the first non-significant test is encountered, at which point no further outcomes will be tested for confirmatory purposes.
Missing outcome data are expected to be limited, as the primary and key secondary outcomes are obtained from medical records. The handling of missing data will be prespecified in the SAP, and sensitivity analyses will be conducted where appropriate. Additional per-protocol analyses based on treatment adherence will also be performed. Detailed specifications of all primary, secondary, exploratory, sensitivity, and per-protocol analyses will be provided in the SAP, which will be finalised before database lock and unblinding.

Reviewer: 1, Comment 2
The hierarchical testing order is not specified in the manuscript. The manuscript states that "the full significance level (α=0.05) will be sequentially allocated to the secondary outcomes, in a predefined order of testing", without specifying what that order is. The protocol clarifies the sequence (length of neonatal stay → birthweight). Specifying the testing order in the manuscript is a transparency requirement — it pre-empts any later question about the ordering being data-driven and is the standard expectation under the SPIRIT-Outcomes 2022 recommendations (item 20a.1).
Thank you for this comment. We have revised the manuscript accordingly and now explicitly specify the predefined hierarchical testing sequence, with testing proceeding from the primary outcome (prolongation of gestation) to neonatal length of stay and subsequently neonatal birthweight.
Page 18, Lines 20-22

If the primary outcome is statistically significant, the full significance level (α=0.05) will be sequentially allocated to the secondary outcomes, in the following prespecified order: neonatal length of stay followed by neonatal birthweight.

Reviewer: 1, Comment 3

The censoring strategy for the primary outcome should be made explicit in the manuscript. The handling of right-censoring at 37+0 weeks (when planned induction occurs without other maternal or fetal indication) and interval-censoring of intrauterine fetal demise is critical for the interpretation of "prolongation of gestation defined as time from randomisation to birth", because both conventions have potential consequences for the estimated treatment effect. A 1–2 sentence description in the Outcomes or Statistical methods section of the manuscript would suffice.

Thank you for this comment. We have revised the manuscript accordingly and now provide an explicit description of the planned censoring strategy for the primary outcome in the Statistical Methods section, including the handling of right-censoring at 37+0 weeks and interval-censoring of intrauterine fetal demise.

Page 17, Lines 6-8, and Page 18, Lines:1-4

The primary outcome, prolongation of gestation, will be analysed using a log-normal mixed-effects censored regression model, and treatment effects will be presented as geometric mean ratios with 95% confidence intervals. Planned induction at 37+0 weeks' gestation without another maternal or fetal indication for delivery will be treated as right-censoring. In cases of intrauterine fetal demise, the event time will be treated as interval-censored between the last assessment confirming fetal viability and the diagnosis of fetal demise.

Reviewer: 1, Comment 4

Reference 32 (Zhang Y et al. 2021) is published in Oxidative Medicine and Cellular Longevity, a Hindawi journal. Hindawi has, since 2022, retracted in excess of 1,700 papers across its portfolio for systematic manipulation of the publication and peer-review process; Oxidative Medicine and Cellular Longevity has been particularly affected, with hundreds of retractions in 2023–2024. The article cited here has not, as of this writing, been retracted, and its mechanistic content is thematically appropriate to the assertion it sustains in the manuscript discussion. However, citation of journals with documented integrity concerns is increasingly discouraged in the methodological literature. Consider replacing the reference with a non-Hindawi source covering a similar mechanistic point — for example, Han et al. 2025 in BMC Pregnancy and Childbirth on insulin-resistance-induced trophoblast pyroptosis and its protection by metformin (doi:10.1186/s12884-025-07419-0) covers a closely related mechanism in a non-Hindawi venue.

Thank you for acknowledging this. The title for the suggested doi appears to be *Insulin resistance-induced mitochondrial dysfunction and pyroptosis in trophoblasts: protective role of metformin* and written by Du et al, but this reference suits the purpose well and has now replaced reference nr 32 in the manuscript (in the updated version nr 35).

Reviewer: 1, Comment 5

The manuscript does not contain a declaration of use or non-use of artificial intelligence in its preparation. BMJ guidelines now require this declaration explicitly. Please add a brief statement in an appropriate section (Acknowledgments or Methods).

Thank you for this comment. We have added an AI use statement to the manuscript in the Methods section.
Page 10, Lines 7-10:
The authors used ChatGPT (GPT-5; OpenAI) during manuscript preparation to assist with language editing and improve readability. All AI-generated suggestions were critically reviewed, verified, and edited by the authors, who take full responsibility for the final content of the manuscript.

Reviewer: 1, Comment 6
The conflict-of-interest disclosure could be more specific. The current statement — "LB, CC, SW, ST and HI provide consulting services to companies developing novel therapeutics for pre-eclampsia" — does not allow readers to assess the nature of the conflicts in relation to the trial drug or its potential comparators. Consider naming the companies involved, in line with ICMJE recommendations for transparent disclosure.
Thank you for the comment. This has now been updated in the section Competing interests.
Page 25, Lines 1-4:
The following authors provide consulting services to the following companies that developing therapeutics for preeclampsia: Diamedica Therapeutics (LB, CC, SW,ST, HI), Beech Biotech (CC, ST, HI), Avilar Therapeutics (CC, ST), GondolaBio (CC, ST), Araka Therapeutics (CC, LB) and Eli Lilly (ST).

Reviewer: 1, Comment 7
Viability assessment between 22+0 and 23+6 weeks. The eCRF (Online Supplemental Appendix 3, Form 1) indicates that for participants between 22+0 and 23+6 weeks at inclusion, the sponsor must be contacted for discussion regarding viability before randomisation. This is good practice, but is not described in the main manuscript. Brief mention in the Inclusion criteria or Participant enrolment section would be helpful, particularly for international readers operating in jurisdictions where viability thresholds may differ.
Thank you for the comment. A statement regarding this has been added to the Inclusion criteria.
Page 11, Lines 4-5:
If the gestational age is between 22+0 to 23+6 gestational weeks, the sponsor should be contacted for discussion around viability before randomisation.

Reviewer: 1, Comment 8
Sample-size narrative could be sharpened. The Strengths bullet "a clinically significant 50% increase in prolongation of gestation from randomisation to birth of 3.5 days" combines a relative effect (50% increase) and an absolute effect (3.5 days) in a way that is initially confusing. Suggest: "an absolute increase of 3.5 days from a median prolongation of 7 days, corresponding to a 50% relative increase". It is also worth noting in the Discussion that PI2 estimated a geometric mean ratio of 1.39 (95% CI 0.99–1.95) in the prolongation of gestation, slightly below the GMR = 1.50 that PI4 is sized to detect; if the true effect is closer to the PI2 point estimate, PI4 may also yield a borderline result, and the planned IPD meta-analysis with PI3 and PI-NL is therefore an important component of the evidentiary strategy. The current Strengths-and-limitations panel mentions this in general terms; making the link

between trial size, PI2's effect estimate, and the IPD meta-analysis more explicit would benefit readers.

Thank you for this comment. We have revised the manuscript accordingly and clarified in the Sample size calculation section that the trial is powered to detect a 50% increase in prolongation of gestation, corresponding to an absolute increase of 3.5 days, assuming a median prolongation of 7 days in the placebo group. We have also further highlighted the planned individual participant data meta-analyses combining results from PI2 and the ongoing parallel PI3 and PI-NL trials in the Discussion section.

Page 15, Lines 5-8:

A total of 294 participants will be recruited to the PI4 trial to detect a 50% relative increase in prolongation of gestation between the metformin ER and placebo arms, corresponding to an absolute increase of 3.5 days in median prolongation under the assumption of a median prolongation of seven days in the placebo arm.

Page 23, Lines 13-19:

Together, these four metformin trials (PI2, PI3, PI4, and PI-NL) will provide an opportunity to evaluate the effects of metformin on the primary, secondary, and selected exploratory outcomes in individual participant data meta-analyses. This collaborative approach will increase the precision of treatment effect estimates and strengthen the evidence base for maternal and neonatal outcomes, particularly for outcomes that are not adequately powered within individual trials. Results from these analyses may provide important evidence to inform future clinical guidelines for the management of preterm pre-eclampsia.

Reviewer: 1, Comment 9

Reference 24 (ACOG Practice Bulletin 180 on Gestational Diabetes Mellitus, Obstet Gynecol 2017) appears in the reference list but I could not locate a clear in-text citation in the manuscript body. Please verify whether the reference is cited; if not, remove from the list.

Thank you for acknowledging this. The reference has now been removed from the reference list.

Reviewer: 1, Comment 10

References 20–22 (Nordic clinical guidelines on hypertensive disorders of pregnancy, in Swedish, Finnish, and Norwegian) are appropriate for justifying the standardised care across sites, but are local-language sources potentially inaccessible to international readers. Consider providing English-language titles or brief descriptive annotations of what each guideline contains. Reference 25 (UN Sustainable Development Goals website homepage) could be made more specific by pointing to the SDG 3.1 indicator on maternal mortality reduction or to a relevant UN technical report.

English-language titles have been added to the guidelines references and the reference to the SDG has been changed to the SDG 3.1.

Reviewer: 1, Comment 11

Typographical and editorial points:

— "concentrations similar to those observed in to non-pregnant individuals" (Intervention section) — read "to those observed in non-pregnant individuals" (delete "in to").

Thank you, the typo has been corrected in the Intervention section.

Page 12, Line 3:

… concentrations similar to those observed in non-pregnant individuals…

— "Mars 11 2024" in the Ethics section — should read "March 11, 2024".
“Mars 11 2024” could only be found in the reference list, but has been changed after updating of the reference.

— Some inconsistencies in reference formatting (extra spaces, hyphenation around author names) reflect Endnote artefacts and merit a final pass before publication.
Thank you, the inconsistencies and errors have been corrected.

Reviewer: 1, Comment 12
PI2 / PI3 / PI-NL relationship and the planned IPD meta-analysis. The Discussion mentions the parallel trials and the planned IPD meta-analysis, but the methodological framing of "we will participate in a prospectively planned IPD meta-analysis with harmonised outcomes" deserves earlier prominence — perhaps in the Methods (after Sample size calculation) or in a brief paragraph in the Introduction. The fact that four trials are running in parallel with harmonised primary and secondary outcomes is a significant strength and is currently dispersed across the abstract Strengths bullet and the Discussion.
Thank you for this comment. A paragraph regarding the planned meta-analyses has now been added to the Introduction.
Page 9, Line 26, and Page 10, Lines 1-2:
Results from this trial will be analysed separately but also be part of meta-analyses combining results from this and three other ongoing or finalized metformin versus placebo trials in South Africa and The Netherlands.

Reviewer: 1, Comment 13
Consent to follow-up of the child (consent 2). The eCRF and protocol indicate that two consents are obtained — consent 1 from the participant before birth, and consent 2 from caregivers post-birth for child follow-up — and that biological samples and follow-up data must be destroyed if consent 2 is not granted. This is well-handled operationally; brief mention of the dual-consent structure in the Ethics section of the manuscript would clarify the procedure for readers.
A sentence regarding consent 2 has been added to the Ethics section
Page 20, Lines 17-19:
The second consent concerns follow-up of children and analyses of cord blood and placental samples. If this consent is not granted, cord blood and placental samples will be destroyed if and follow-up data for the child will not be analysed.

Reviewer: 1, Comment 14
SPIRIT item 18a.1 — Responsiveness of study instruments. The submitted SPIRIT-Outcomes 2022 checklist marks this item as "-". The trial uses validated instruments (ASQ-3, M-CHAT-R, Bayley-4, plus PHASE-20 in the eCRF for medication-related symptoms). A brief sentence summarising what is known about responsiveness of these instruments in similar populations would address the checklist item.
This study does not include any longitudinal/repeated evaluations of child outcomes, and as the intervention is started before birth evaluations before and after intervention is not possible. Outcome measurements thus rely on cross-sectional age-specific evaluations and

group comparisons. A sentence regarding validity and reliability of study instruments that refers to Online Supplemental Appendix 3 has been added to the Outcomes section.
Page 14, lines 6-7
Validity and reliability of study instruments are summarised in Online Supplemental Appendix 3.

Reviewer: 1, Comment 15
SPIRIT item 26b — Additional consent provisions for ancillary studies. Listed as "-" in the submitted checklist, but the trial does have a separate consent (consent 2) for child follow-up and biological-sample use, and the eCRF makes provisions for destruction of samples if consent 2 is withheld. Please complete this checklist item with the manuscript location.
The checklist has been updated with the following information: Page 20, Ethics and dissemination.

Reviewer: 1, Comment 16
SPIRIT item 31b — Authorship eligibility guidelines. Listed as "-". A brief mention of adherence to ICMJE recommendations would address this checklist item.
A sentence regarding this has now been added to the Authors' contributions section and the SPIRIT item 31b has been updated with manuscript location.
Page 24, lines 1-2:
This study was conducted and reported in accordance with the recommendations of the International Committee of Medical Journal Editors (ICMJE).

REVIEWER 2

Reviewer 2, Comment 1
The biological basis for the intervention should be better developed. Although previous clinical evidence is cited, the manuscript does not describe or hypothesise the mechanisms through which metformin may prolong pregnancy or influence the pathophysiology of pre-eclampsia.
Thank you for the comment. The biological basis for the intervention has now been described in more detail in the Introduction.
Page 8, lines 13-26:
Published preclinical data strongly suggests that metformin may be a candidate therapeutic for preeclampsia.[16] Firstly, in vitro and ex vivo experiments using primary human tissues showed that metformin reduced the placental release of anti-angiogenic factors, namely soluble Fms-like tyrosine kinase 1 (sFlt-1) and soluble Endoglin (sEng), that cause maternal endothelial dysfunction in preeclampsia. Secondly, metformin enhanced vasodilation in vessels isolated from maternal omental arteries obtained at caesarean section and induces angiogenesis.[16] These characteristics make it a potential therapeutic to treat preeclampsia. Further preclinical studies show that metformin can rescue a preeclampsia phenotype in an animal model.[17] Although metformin is generally considered to mediate its antihyperglycemic effects through specific cellular pathways, recent evidence indicates that it also acts through pathways in the gut. For example, a study has provided evidence that the lower bowel is a major site of action for metformin. [18] Furthermore, recent studies in mice [19] and humans [20 21] suggest that gut microbial changes contribute to the effects of metformin [21].

Reviewer 2, Comment 2

The rationale for the selected dosage of extended-release metformin (1 g three times daily) should be clearly explained.

The rationale for the selected dosage of metformin which is explained in the Interventions section, has now been updated.

Page 13, Lines:1-5

The dose is based on pharmacokinetic studies showing that a daily dose of three grams of metformin ER administered to women with preterm pre-eclampsia resulted in plasma concentrations similar to those observed in non-pregnant individuals taking two grams daily. [8 19] *The results do not suggest metformin accumulation with the three-gram dose.* [8] *A daily dose of 3 grams was also used in the PI2 trial.* [8]

Reviewer 2, Comment 3

The authors should further justify the effect size assumed for the sample size calculation, discuss the clinical relevance of prolongation of gestation as the primary endpoint in terms of expected neonatal benefit, and briefly describe the planned approach to safety monitoring and management of missing data.

Thank you for this comment. We have revised the manuscript accordingly and further justified the effect size assumed for the sample size calculation. We have also expanded the discussion of the clinical relevance of prolongation of gestation as the primary endpoint, including its expected neonatal benefit. Finally, we have clarified the planned approach to safety monitoring and expanded the Statistical Methods section to describe the planned handling of missing data.

Page 15, lines 5-8:

A total of 294 participants will be recruited to the PI4 trial to detect a 50% relative increase in prolongation of gestation between the metformin ER and placebo arms, corresponding to an absolute increase of 3.5 days in median prolongation under the assumption of a median prolongation of seven days in the placebo arm.

Page 16, lines 18-24, and Page 19, Lines: 1-2

The DSMB is composed of three external members, including an obstetrician, a paediatrician, and a statistician, who meet every 12 months. The DSMB monitors participant safety through periodic review of adverse events and serious adverse events, including masked safety summaries by treatment group, and makes recommendations to the sponsor regarding the continuation, modification, or termination of the trial. The trial may be prematurely terminated upon DSMB recommendation if emerging safety data indicate that continuation of the trial is not in the best interests of participants. No formal interim analyses for efficacy or futility are planned, and no sample size re-estimation will be performed. The DSMB will not review unblinded efficacy data.

Page 18, lines 26-27, and Page 19, Lines 1-2:

Missing outcome data are expected to be limited, as the primary and key secondary outcomes are obtained from medical records. The handling of missing data will be prespecified in the SAP, and sensitivity analyses will be conducted where appropriate. Additional per-protocol analyses based on treatment adherence will also be performed.

Page 22, Lines 15-26, and Page 23, Lines 1-2:
The primary endpoint, prolongation of gestation, was chosen after consensus in the steering committee including input from the Swedish Preeclampsia Patient Association as a clinically relevant and pragmatic outcome. This outcome enables a well-powered trial in this relatively rare condition while being an important determinant of neonatal outcomes. The trial is powered to detect a 50% relative increase in prolongation of gestation, corresponding to an absolute increase of 3.5 days assuming a median prolongation of seven days in the placebo arm. Previous studies demonstrate a continuous improvement in neonatal outcomes with advancing gestational age, suggesting that even modest prolongation of pregnancy of one or a few days may translate into clinically meaningful benefits in terms of survival, neonatal morbidity, and duration of hospitalization. [36 37] *However, as the PI4 trial encompasses a broad range of gestational ages, the magnitude and clinical relevance of these benefits are expected to vary according to gestational age. Therefore, all exploratory outcomes will also be assessed in an individual participant meta-analysis as previously mentioned.*

VERSION 2 - REVIEW

**Reviewer**	**2**
**Name**	**Di Simone , Nicoletta**
**Affiliation**	**Humanitas University**
**Date**	**20-Sep-2026**
**COI**	

I thank the authors for their careful and comprehensive revision of the manuscript. All the comments and concerns raised in the previous round have been adequately addressed. The revised manuscript is substantially improved in terms of clarity, completeness, and overall presentation.
