# Supplementary material for: Can metformin prolong pregnancy in preterm pre-eclampsia? Study protocol for a Nordic multicentre randomised placebo-controlled trial (PI4)
Source: BMJ Open. 2026 Oct 13;16(10):e120993. doi: 10.1136/bmjopen-2026-120993 (PMC13663727; doi:10.1136/bmjopen-2026-120993)
Supplement: Supplementary file 5 [file bmjopen-16-10-s002.docx]

***Indications for delivery in hypertensive disorders during pregnancy^1^***

**Deliver regardless of length of pregnancy**

Poorly controlled blood pressure despite antihypertensive treatment

Eclampsia
Stroke (hemorrhagic or infarct/ischemic)

HELLP^2^

Placental abruption

Heart failure

Maternal pulmonary edema or high suspicion of
Intrauterine Fetal Demise (IUFD)

**Delivery should be considered regardless of the length of pregnancy**

Decreasing thrombocytes, thrombocytopenia

Rising transaminases

Renal failure according to national definitions (*Swedish: Increase in P-Creatinine by >90 μmol/L or increase in P-Creatinine by >50% (relative increase) Finnish: Increase in P-Creatinine > 110 µmol/l or increase in P-Creatinine by >50% (relative increase), Norwegian: Increase in P-Creatinine >90µmol/l)*

Remaining neurological symptoms

Remaining epigastric pain, nausea or vomitting with affected hepatic values

Severe fetal impairment

Severe fetal growth restriction

**Supplemental table 2**

**^1^Guidelines from Swedish Society of Obstetrics and Gynecology, Finnish Medical Society Duodecim and Finnish Society of Obstetrics and Gynaecology, and Norsk gynekologisk forening
^2^ Hemolysis, Elevated Liver enzymes and Low Platelets Syndrome**
